
JOURNAL INFORMATION
==============================
NLM Title Abbreviation: Insights Imaging
Journal ID: Insights Imaging
NLM Journal ID: 101532453

Insights into Imaging

EISSN: 1869-4101
Publisher: Springer

ARTICLE INFORMATION
==============================
PMCID: PMC11128411
DOI: 10.1186/s13244-024-01715-7
Article ID: 1715
Article version: 1
Subjects: Meeting Abstracts

ESGAR 2024 Book of Abstracts

Electronic publication date: 2024 May 27
Publication date: 2024 May
Volume: 15
Issue: Suppl 1
Issue sponsor: Publication costs were funded by the European Society of Gastrointestinal and Abdominal Radiology.
Electronic Location ID: 123
Copyright: © The Author(s) 2024
License: Open Access This article is licensed under a Creative Commons Attribution 4.0 International License, which permits use, sharing, adaptation, distribution and reproduction in any medium or format, as long as you give appropriate credit to the original author(s) and the source, provide a link to the Creative Commons licence, and indicate if changes were made. The images or other third party material in this article are included in the article’s Creative Commons licence, unless indicated otherwise in a credit line to the material. If material is not included in the article’s Creative Commons licence and your intended use is not permitted by statutory regulation or exceeds the permitted use, you will need to obtain permission directly from the copyright holder. To view a copy of this licence, visit http://creativecommons.org/licenses/by/4.0/.
License URL: https://creativecommons.org/licenses/by/4.0/

Conference name: 35th Annual Meeting and Postgraduate Course ESGAR 2024
Conference location: Gothenburg, Sweden
Conference date: 28-31 May 2024
issue-copyright-statement: © European Society of Radiology (ESR) 2024

==============================

EUROPEAN SOCIETY OF RADIOLOGY

European Society ESGAR Gastrointestinal and Abdominal Radiology

### Insights into Imaging

ESGAR 2024 Book of Abstracts

Volume 15 / Supplement 1
May 2024

Springer

ESGAR 2024 / May 28 - 31 / Gothenburg, Sweden
35th Annual Meeting and Postgraduate Course

European Society ESGAR Gastrointestinal and Abdominal Radiology

### ESGAR

### 35th Annual Meeting and Postgraduate Course

### 2024

### May 28-31

GOTHENBURG
SWEDEN

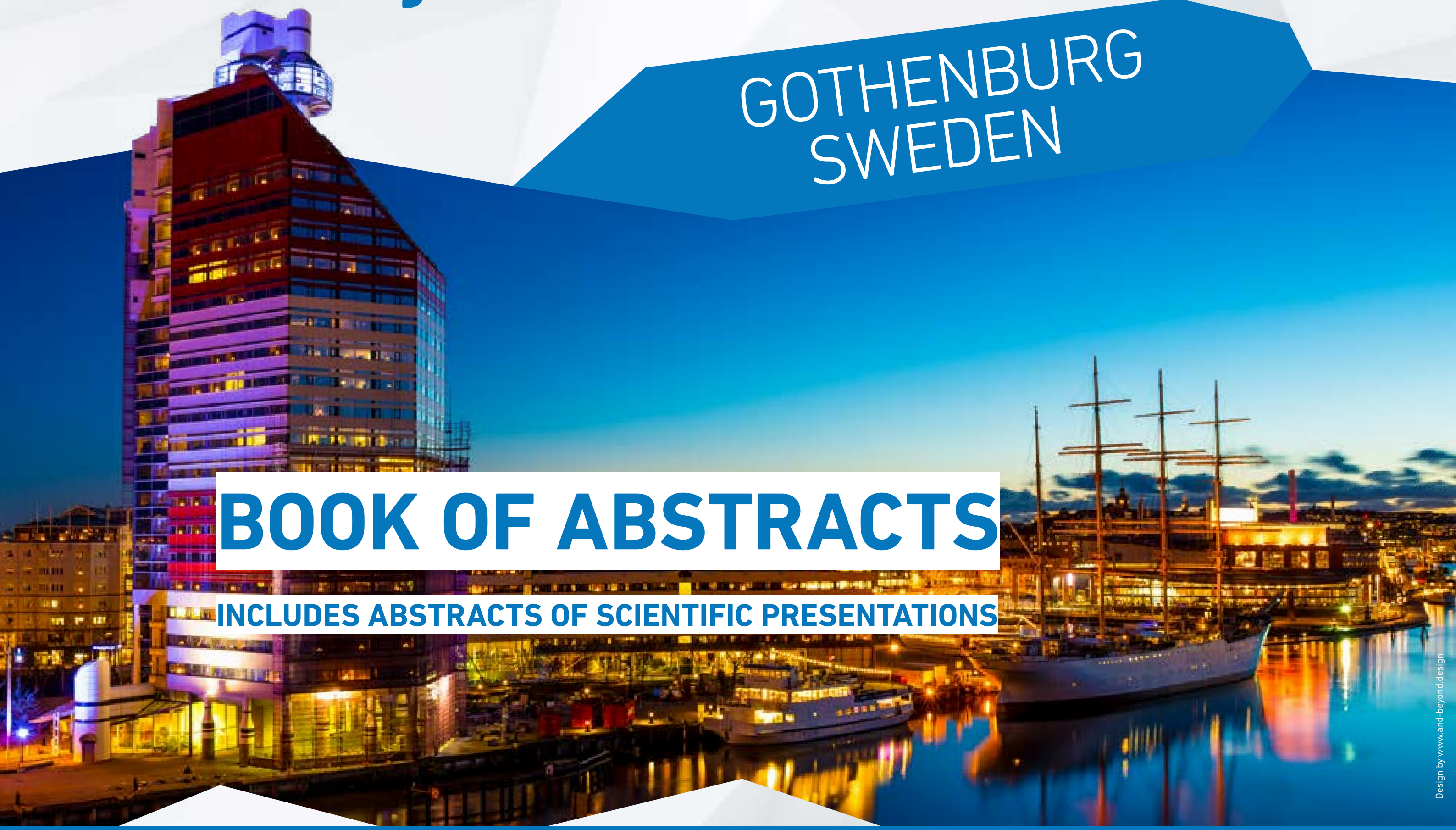

### BOOK OF ABSTRACTS

**INCLUDES ABSTRACTS OF SCIENTIFIC PRESENTATIONS**

Design by www.and-beyond.design

*IMPORTANT ADDRESSES*

**ORGANISING SECRETARIAT**
**Central ESGAR Office**
Esslinggasse 2/3
AT – 1010 Vienna
Phone: +43 1 535 89 27
E-Mail: office@esgar.org www.esgar.org

### CME CREDITS

An application has been made to the EACCME for CME accreditation of this event. The EACCME is an institution of the UEMS (www.uems.eu). The number of credit hours of European external CME credits will be announced on the ESGAR website and in the Final Programme.

### CONTRIBUTING SOCIETIES

Asian Society of Abdominal Radiology (ASAR) – Since 2007

European Society of Urogenital Radiology

### SPONSORS

ESGAR wishes to gratefully acknowledge the support of its Corporate Members:

BAYER

BRACCO – LIFE FROM INSIDE

GE HealthCare

Guerbet – Contrast for Life

The Final Programme of ESGAR 2024 is available on the ESGAR Website www.esgar.org

Date of publishing: May 2024

### *TABLE OF CONTENTS*

| | | |
|---|---|---|
| Scientific Sessions, Wednesday, May 29 | (SS 1.1 – SS 1.10) | 8-10 |
| Scientific Sessions, Wednesday, May 29 | (SS 2.1 – SS 2.9) | 10-12 |
| Scientific Sessions, Wednesday, May 29 | (SS 3.1 – SS 3.10) | 12-15 |
| Scientific Sessions, Wednesday, May 29 | (SS 4.1 – SS 4.9) | 15-17 |
| Scientific Sessions, Wednesday, May 29 | (SS 5.1 – SS 5.10) | 17-20 |
| Scientific Sessions, Thursday, May 30 | (SS 6.1 – SS 6.10) | 21-22 |
| Scientific Sessions, Thursday, May 30 | (SS 7.1 – SS 7.10) | 23-25 |
| Scientific Sessions, Thursday, May 30 | (SS 8.1 – SS 8.10) | 26-28 |
| Scientific Sessions, Thursday, May 30 | (SS 9.1 – SS 9.10) | 28-30 |
| Scientific Sessions, Thursday, May 30 | (SS 10.1 – SS 10.10) | 30-33 |
| Scientific Sessions, Friday, May 31 | (SS 11.1 – SS 11.10) | 34-36 |
| Scientific Sessions, Friday, May 31 | (SS 12.1 – SS 12.10) | 36-38 |
| Scientific Sessions, Friday, May 31 | (SS 13.1 – SS 13.10) | 39-41 |
| Scientific Sessions, Friday, May 31 | (SS 14.1 – SS 14.9) | 41-43 |
| AUTHORS' INDEX | | 45-49 |

European Society of Gastrointestinal and Abdominal Radiology ESGAR

Insights Imaging (2024) Vol 15 (Suppl 1)
https://doi.org/10.1186/s13244-024-01715-7

### ESGAR EXECUTIVE COMMITTEE

PRESIDENT
*M. Zins, Paris/FR*

PRESIDENT-ELECT
*J. Stoker, Amsterdam/NL*

VICE PRESIDENT
*G. Brancatelli, Palermo/IT*

SECRETARY/TREASURER
*V. Vilgrain, Clichy/FR*

PROGRAMME COMMITTEE CHAIR
*J. Stoker, Amsterdam/NL*

EDUCATION COMMITTEE CHAIR
*C.J. Zech, Basel/CH*

MEMBERSHIP COMMITTEE CHAIR
*D. Tolan, Leeds/UK*

RESEARCH COMMITTEE CHAIR
*S.A. Taylor, London/UK*

WORKSHOP COMMITTEE CHAIR
*S. Gourtsoyianni, Athens/GR*

MEETING PRESIDENT 2024
*L. Blomqvist, Stockholm/SE*

MEETING PRESIDENT 2025
*J. Stoker, Amsterdam/NL*

MEMBERS AT LARGE
*M.A. Bali, Brussels/BE*
*M. Karcaaltincaba, Ankara/TR*
*N. Kartalis, Stockholm/SE*
*M. Radzina, Riga/LV*

ESGAR EXECUTIVE DIRECTOR
*B. Lindlbauer, Vienna/AT*

### ESGAR 2024 MEETING PRESIDENT

*Prof. Lennart Blomqvist*
*Karolinska University Hospital*
*Department of Diagnostic Radiology*
*171 76 Stockholm, Sweden*

### ESGAR 2024 PROGRAMME COMMITTEE

CHAIR
*J. Stoker, Amsterdam/NL*

CO-CHAIR
*G. Brancatelli, Palermo/IT*

MEMBERS
*M.A. Bali, Brussels/BE*
*L. Blomqvist, Stockholm/SE*
*S. Gourtsoyianni, Athens/GR*
*M. Karcaaltincaba, Ankara/TR*
*N. Kartalis, Stockholm/SE*
*M. Radzina, Riga/LV*
*S.A. Taylor, London/UK*
*D. Tolan, Leeds/UK*
*V. Vilgrain, Clichy/FR*
*C.J. Zech, Basel/CH*
*M. Zins, Paris/FR*

Young ESGAR
*R. Cannella, Palermo/IT*
*K. De Paepe, Boston, MA/US*
*C. Reiner, Zurich/CH*

### ESGAR 2024 LOCAL ORGANISING COMMITTEE

*J. Brandberg, Gothenburg/SE*
*I. Cetinic, Gothenburg/SE*
*M. Hellström, Gothenburg/SE*
*N. Kartalis, Stockholm/SE*
*P. Leander, Malmö/SE*
*L. Loizou, Stockholm/SE*
*B. Norén, Linköping/SE*
*K. Riklund, Umeå/SE*
*F. Thoren, Gothenburg/SE*
*S. Zackrisson, Malmö/SE*

O. Akhan, Ankara/TR
M.A. Bali, Brussels/BE
T.V. Bartolotta, Palermo/IT
A. Ba-Ssalamah, Vienna/AT
N. Bastati-Huber, Vienna/AT
R.G.H. Beets-Tan, Amsterdam/NL
E. Biscaldi, Genoa/IT
L. Blomqvist, Stockholm/SE
I. Boulay Coletta, Paris/FR
G. Brancatelli, Palermo/IT
R. Cannella, Palermo/IT
L. Crocetti, Pisa/IT
L. Curvo-Semedo, Coimbra/PT
K. De Paepe, Boston, MA/US
M. Dioguardi Burgio, Clichy/FR
A. Dohan, Paris/FR
R.F. Dondelinger, Liège/BE
M. D'Onofrio, Verona/IT
A. Furlan, Pittsburgh, PA/US
M. Galia, Palermo/IT
V. Goh, London/UK
S. Gourtsoyianni, Athens/GR
S. Halligan, London/UK
T. Helmberger, Munich/DE
F. Iafrate, Rome/IT
D. Ippolito, Monza/IT
S. Jackson, Plymouth/UK
M. Karcaaltincaba, Ankara/TR
N. Kartalis, Stockholm/SE
A. Laghi, Rome/IT
D. Lambregts, Amsterdam/NL
J.M. Lee, Seoul/KR
G.F. Lo Re, Palermo/IT
O. Lucidarme, Paris/FR
A.J. Madureira, Porto/PT
T. Mang, Vienna/AT
V. Maniatis, Sønderborg/DK
L. Martí-Bonmatí, Valencia/ES
C. Matos, Lisbon/PT
F. Matteini, Palermo/IT
Y. Menu, Paris/FR
G. Morana, Treviso/IT
S. Mulé, Créteil/FR
P. Paolantonio, Rome/IT
N. Papanikolaou, Lisbon/PT
A. Plumb, Esher/UK
S. Pötter-Lang, Vienna/AT
P. Prassopoulos, Thessaloniki/GR
M. Radzina, Riga/LV
M. Ronot, Clichy/FR
W. Schima, Vienna/AT
S. Schmidt Kobbe, Lausanne/CH
A. Schreyer, Brandenburg an der Havel/DE
O. Seror, Bondy/FR
E. Soloff, Seattle, WA/US
S. Stojanovic, Novi Sad/RS
J. Stoker, Amsterdam/NL
C. Stroszczynski, Regensburg/DE
B. Taouli, New York, NY/US
J.P. Tasu, Poitiers/FR
S.A. Taylor, London/UK
D. Tolan, Leeds/UK
C. Triantopoulou, Athens/GR
V. Valek, Brno-Bohunice/CZ
F. Vernuccio, Palermo/IT
V. Vilgrain, Clichy/FR
M.-P. Vullierme, Epagny Metz-Tessy/FR
M. Wagner, Paris/FR
G. Zamboni, Verona/IT
C.J. Zech, Basel/CH
M. Zins, Paris/FR

European Society
ESGAR
Gastrointestinal and Abdominal Radiology

09:00 - 10:30 Room J1

*Scientific Session SS 1*

### Pancreas: Cysts, IPMN, pancreatic ductal adenocarcinoma

### SS 1.1

### EUS vs MRI for assessing pancreatic cysts - A prospective comparative study

M. Lefere, S. De Vuysere, M. Bronswijk, P. Bossuyt; *Bonheiden/BE*

**Purpose:** Measurements of main pancreatic duct (MPD) diameter and its communication with cystic pancreatic lesions are essential to clinical/radiological management. We compared MRI and EUS MPD diameter measurements and assessed inter-reader reliability (IRR) between 2 MRI readers (R1 and R2) and 1 EUS reader for MPD dilatation and communication with cysts.
**Material and Methods:** Twenty-two patients underwent EUS and MRI within a ≤6-month interval. On EUS, MPD diameter, presence of cysts >5mm and MPD communication were recorded for each patient in pancreatic head, body, and tail. MPD diameter was measured on a predefined single-shot RARE image and presence of cysts on any T2-weighted image. Cyst presence discrepancies were resolved by consensus between R1 and R2. Bland–Altman analysis was used to evaluate MRI vs EUS MPD diameter differences. To assess IRR, Cohen's kappa (κ) was calculated for MPD dilatation (>5mm) and cyst–MPD communication.
**Results:** Mean and 95% limits of agreement (LOA) for MPD diameter difference: 0,24(-2,41/+2,89) for MRI R1 vs EUS; 0,16(-3,59/+3,27) for MRI R2 vs EUS; 0,21(-1,27/+1,69) for MRI R1 vs R2. IRR for MPD dilatation was fair for MRI R1 vs R2 (κ=0,37); moderate for MRI R1 vs EUS (κ=0,49); and slight for MRI R2 vs EUS (κ=0,15). Twenty-three cysts >5 mm were detected. IRR for cyst–MPD communication was moderate for MRI R1 vs R2 (κ=0,5); moderate for MRI R1 vs EUS (κ=0,44) and almost perfect for MRI R2 vs EUS (κ=0,89).
**Conclusion:** EUS vs MRI showed a wide LOA for MPD diameter. Agreement for MPD>5 mm was only poor to moderate. Agreement for MPD connection of cystic lesions was moderate to near perfect.

### SS 1.2

### Non-invasive cyst fluid measurement in patients with pancreatic cystic lesions based on spectral imaging

I. Dudás, B. Lovász, Á. Szűcs, M. Benke, P. Kaposi, A. Szijártó, P. Maurovich-Horvat, B. Budai; *Budapest/HU*

**Purpose:** Differentiation between pancreatic cystic lesions is a challenging task for clinicians. Spectral imaging of photon-counting detector CT scanners allows the reconstruction of virtual monoenergetic images (VMI) enabling the measurement of Hounsfield unit (HU) densities at different keV. We aimed to investigate whether high-risk lesions have different spectral density patterns that could help the diagnostics.
**Material and Methods:** Our study included 58 patients diagnosed with mucinous cystic neoplasms and 20 with non-mucinous cystic pancreatic lesions based on current practice guidelines. The densities were measured on the VMIs of portal venous phase scans by placing 3–3 circular regions of interest in pancreatic cystic lesions, water, and simple kidney cysts. The HUs were plotted against the keV values, and the average densities at 40keV ($HU_{40keV}$), at 70keV ($HU_{70keV}$), and their difference ($HU_{diff(40keV-70keV)}$) were recorded. Kruskal–Wallis test with post hoc Dunn's test was used for comparing the groups. The diagnostic performance was assessed by receiver operating characteristic curves. The interobserver reproducibility was evaluated by intraclass correlation coefficients (ICC).
**Results:** Significant differences were found in $HU_{diff(40keV-70keV)}$ between mucinous and non-mucinous pancreatic lesions (p<0.0001); mucinous pancreatic lesions and simple kidney cysts (p=0.0066); and between water (<0.00001). After train-test split, the $HU_{diff(40keV-70keV)}$ showed great performance in differentiating mucinous vs. non-mucinous pancreatic lesions with AUCs of 0.89 and 0.92; accuracies of 85.5% and 80%; sensitivities of 94.7% and 100%; and specificities of 57.9% and 60%, with a good interobserver reproducibility (ICC=0.82).
**Conclusion:** The measurement of $HU_{diff(40keV-70keV)}$ on VMI reconstructions could be a useful complementary non-invasive method for differentiating between mucinous and non-mucinous pancreatic cystic lesions.

### SS 1.3

### Typical pancreatic serous cystadenomas - Should we recommend surgical consultation for asymptomatic patients with large lesions?

R. Kessner, Y. Katz, R. Tzadok, H. Yashar, S. Lazar, A. Chernomorets, D. Ben-Ami Shor; *Tel Aviv/IL*

**Purpose:** The ACR guidelines recommend surgical consultation for patients with large serous cystadenomas. Our purpose was to assess the relation between the primary size of serous cystadenomas and their enlargement during follow-up.
**Material and Methods:** The records of all the patients that underwent MRCP at our institution between 2011 and 2021 were reviewed for the diagnosis of serous cystadenoma. 35 patients with typical (micro-cystic) serous cystadenomas that had at least two MRCP examinations were included. We collected the clinical data of the patients during the follow-up period, including history, symptoms and laboratory results. We recorded the primary and maximal cyst size diameter on MRCP. We compared the clinical parameters and rates of enlargement on follow-up between the patients with primary lesions of over 4 cm and the patients that had smaller lesions.
**Results:** Our study included 21 females and 14 males with an average age of 65.9. The average follow-up period was 37.4 months. None of our patients developed malignant transformation. Our surgeons preferred the conservative option of MRCP follow-up in most of the patients, except one patient that had biliary obstruction due to mass effect. 14 patients had lesions over 4 cm at presentation and the rest had smaller lesions. 19 lesions (54.3%) grew during follow-up. We found no relation between the lesion size at presentation and the enlargement during follow-up (p>0.05). In addition, we found no correlation between all the clinical parameters and the enlargement during follow-up (p>0.05).
**Conclusion:** Our results raise the question whether the ACR recommendation for asymptomatic patients with large serous cystadenomas should be changed.

### SS 1.4

### The diagnostic value of abbreviated MRI protocol in the surveillance of intraductal papillary mucinous neoplasm

S. Malekzadeh[1], R. Cannella[2], I. Fournier[1], P. Hiroz[1], C. Mottet[1], C. Constantin[1], L. Widmer[3]; [1]*Sion/CH,* [2]*Palermo/IT,* [3]*Villars-sur-Glâne/CH*

**Purpose:** To assess the diagnostic value of abbreviated protocol (AP) MRI to detect the degeneration signs in intraductal papillary mucinous neoplasms (IPMNs) in patients who undergo a routine MRI follow-up.
**Material and Methods:** This retrospective dual-center study recruited all patients with IPMN diagnosed on initial comprehensive protocol (CP) MRI who underwent routine MRI follow-up. CP included axial and coronal T2-weighted images (T2WI), axial T1-weighted images (T1WI) before and after contrast administration, 3D MR cholangiopancreatography (MRCP) and diffusion-weighted images (DWI). Two APs, eliminating dynamic sequences ± DWI, were extracted from CP. Two radiologists evaluated the APs separately for IPMN degeneration signs according to Fukuoka criteria and compared the results to the follow-up CP. In patients who underwent EUS, imaging findings were correlated with pathological results. Per-patient and per-lesion sensitivity, specificity, positive predictive value (PPV), negative predictive value (NPV), and accuracy of APs were calculated.
**Results:** A total number of 114 patients (56.1% women) with 256 lesions were included. The median age of patients was 71 years and the median interval between MRIs was 794 days. Degeneration signs were observed in 24.6% and 12.1%, per-patient and per-lesion, respectively. Regarding APs, the per-patient sensitivity, specificity, PPV, NPV, and accuracy in detecting the degeneration signs were 100%, 93.5%, 83.3%, 100%, and, 95.1%, respectively. No additional role for DWI was detected.
**Conclusion:** AP can confidently replace CP in patients with IPMN with high sensitivity and PPV while offering benefits, such as patient comfort, improved MRI accessibility, and reduced dedicated time for image analysis.

### SS 1.5

### Rate of malignancy of intraductal papillary mucinous neoplasm during MRI follow-up: Preliminary results of a balancing act on data from MRI sections

B. Mascarin, F. Baratta, M. Roman, M. Morra, S. Martínez Rivero, R. De Robertis, M. D'Onofrio*; Verona/IT*

**Purpose:** To evaluate the rate of malignant progression/transformation during MRI follow-up of intraductal papillary mucinous neoplasm (IPMN).
**Material and Methods:** This retrospective analysis included 400 patients (154 males, 246 females) diagnosed with IPMN during a period of 5 years. These patients underwent MRI follow-ups, totaling 1540 contrast-enhanced abdomen MRI. The characteristics of IPMN (number of cysts, location, size), radiological and histological progression data were studied and compared. Chi-square test was used for statistical evaluations.
**Results:** Among the analyzed patients, 20/400 (5%) exhibited radiological transformation as per Revised Fukuoka Guidelines. In this subgroup, 11/400 patients (2.8%) exhibited progression within the initial 3 years of follow-up, with 9/11 presented at the time of the first MRI, while 9/400 (2,25%) patients progressed after 3 years (p value > 0.05). For the 9 patients progressing from the third year, 5/9 exhibited dysplasia (3 low-grade, 1 moderate-grade, 1 high-grade), and 3/9 were diagnosed with adenocarcinoma. Additionally, no statistically significant difference was observed when comparing the progression rate between the group undergoing two MR examinations and the group undergoing more than three MR examinations.
**Conclusion:** While the malignant potential and natural history of IPMN remain unclear, preliminary data suggest that the risk of degeneration or transformation remains consistent regardless of the number of years and MR examinations.

### SS 1.6

### Important radiological and clinicopathological risk factors for recurrence of intraductal papillary mucinous neoplasm after surgical resection

J. Park, J.H. Kim, R.R. Ryu, S. Hwang*; Seoul/KR*

**Purpose:** To assess important radiological and clinicopathological risk factors for post-surgery recurrence in patients with intraductal papillary mucinous neoplasm (IPMN).
**Material and Methods:** A total of 332 patients with IPMN who received surgery from 2011 to 2021 were retrospectively included. Two reviewers assessed the CT findings, including location, type, cyst size, presence and size of enhancing mural nodule (EMN), pancreatic duct diameter, thickened/enhancing cyst wall, abrupt change in pancreatic duct caliber with distal pancreatic atrophy, and lymphadenopathy. Clinicopathological data were collected from medical records and surgical pathologic reports. Univariable Cox regression analysis screened potential risk factors for recurrence, with variables having p < 0.2 subjected to multivariable analysis.
**Results:** Recurrence occurred in 39 patients (11.7%; 8 [2.4%] intrapancreatic and 31 [9.3%] extrapancreatic recurrences) during a median 3.2-year follow-up (range, 0.1–12.3 years). Two- and five-year recurrence rates were 8.8% and 13.6%, respectively. Multivariable Cox regression analysis identified EMN presence (odds ratio [OR] 5.088, 95% confidence interval [CI] 1.549–16.712, p=0.007) and lymphadenopathy (OR 2.837, 95% CI 1.279–6.295, p=0.010) as significant radiological risk factors. Associated invasive carcinoma (OR 25.030, 95% CI 3.036–206.378, p=0.003), lymph node metastasis (within 2 years OR 27.562, 95% CI 8.572–88.621, p<0.001), adjuvant treatment (within 2 years OR 0.203, 95% CI 0.064–0.648, p=0.007), and history of pancreatitis (OR 2.608, 95% CI 1.123–6.053, p=0.026) were also significant. Most imaging features showed moderate to excellent interobserver agreement (ICC=0.77–0.91; κ=0.49–0.69), except for thickened/enhancing cyst wall (κ=0.25).
**Conclusion:** Certain imaging features, including EMN presence and lymphadenopathy, along with several clinicopathologic factors, were identified as significant risk factors for IPMN recurrence.

### SS 1.7

### Loco-regional staging of pancreas cancer: Comparison between computed tomography and magnetic resonance

F. Spoto, E. Bardhi, F. Pasquazzo, M. Todesco, R. De Robertis, M. D'Onofrio*; Verona/IT*

**Purpose:** This study aims to compare the sensitivity of computed tomography (CT) and magnetic resonance imaging (MRI) in the staging of pancreatic ductal adenocarcinoma and the identification of metastases.
**Material and Methods:** CT and/or MRI images of 119 patients were independently reviewed by two radiologists with different expertise in pancreatic imaging. For each subject, the two readers analyzed the degree of arterial and venous vessel infiltration and searched for any thrombosis and metastases, defining the tumor stage based on the resectability criteria of the National Comprehensive Cancer Network. Intra- and inter-observer agreement was statistically calculated using Cohen's kappa coefficient.
**Results:** Inter-observer agreement in CT was strong for the celiac trunk and hepatic artery infiltration (k=0.66) and moderate for superior mesenteric artery (k=0.54). Inter-observer agreement in the determination of venous vascular infiltration in CT and MRI was respectively moderate (k<0.45) and poor (k<0.22); despite this, the patients' therapeutic paths were correctly distinguished. Furthermore, the intra-observer concordance calculated for the patients for whom both CT and MRI were available highlighted how experience plays an important role in the ability to similarly quantify the degree of vascular infiltration with the two methods. Finally, MRI has proven to be more sensitive than CT in identifying liver metastases.
**Conclusion:** Inter-observer variability in defining the degree of vascular infiltration had a limited impact in determining the resectability of the neoplasm. The different intra-observer concordance in the CT–MRI comparison can be attributed to the different experience. MRI is more sensitive in recognizing liver metastases.

### SS 1.8

### Refining the Classroom: The self-learning professor model for optimized segmentation of locally advanced pancreatic ductal adenocarcinoma

J. Bereska, S. Palic, L. Bereska, E. Gavves, Y. Nio, M. Kop, F. Struik, M. Besselink, H. Marquering, J. Stoker*; Amsterdam/NL*

**Purpose:** Segmenting locally advanced pancreatic ductal adenocarcinoma (LAPC) is challenging due to its complex characteristics, leading to limited annotations and frequent exclusion from segmentation models. Self-supervised learning, which typically involves generating pseudo-annotations through a 'teacher' model to enhance the training of a 'student' model, can improve performance despite limited data. However, for LAPC, these pseudo-annotations are too noisy to allow the 'student' model to learn. We introduce a professor model designed to refine these pseudo-annotations.
**Material and Methods:** We developed a pancreatic ductal adenocarcinoma (PDAC) segmentation model using a 3D UNet cascade and a subset of manually annotated data. We subsequently used this 'teacher' model to generate pseudo-annotations for the entire dataset for which manual annotations were unavailable. We trained a second model, the 'professor' model, to identify incorrectly segmented pixels in the pseudo-segmentations using a 3D full-resolution UNet and a set of manual and pseudo-annotations. We compared the pseudo-annotations to the corrected pseudo-annotations using the Dice similarity coefficient (DSC) and intersection over union (IoU).
**Results:** We used 517 CTs from 186 patients with PDAC and 195 control patients, along with 106 CTS from 106 patients with PDAC, to develop the teacher and professor model. We evaluated the (corrected) pseudo-annotations using a test set with 10 CTs from 10 patients with LAPC. The pseudo-annotations achieved a DSC of 0.64 and an IoU of 0.48 compared to 0.74 and 0.59 for the corrected pseudo-annotations.
**Conclusion:** The 'professor' model effectively refines the 'teacher' model's pseudo-annotations for LAPC segmentation, improving Dice similarity coefficient and intersection over union scores.

### SS 1.9

### The multiparametric evaluation of pancreatic ductal adenocarcinoma on dual-layer CT

R. Bárta, T. Rohan, M. Eid, A. Šprláková-Puková, T. Andrašina; *Brno/CZ*

**Purpose:** The aim of this pilot study was to evaluate whether the multiparametric evaluation of segmented and histologically verified pancreatic ductal adenocarcinoma (PDAC) on dual-layer CT (DLCT) correlates with volume of the tumour, presence of liver metastases, age, CA19-9 level and grade.

**Material and Methods:** The retrospective study included 17 patients with histologically verified PDAC, who underwent multiphase contrast-enhanced DLCT with monoenergetic and iodine density maps (85 datasets). 3D segmentation of the tumour was based on 45 keV monoenergetic images in the pancreatic phase 5 times by one radiologist and intrareader variability was calculated (intraclass correlation coefficient and ANOVA test). Mean values of volume, density in Hounsfield units and iodine density extracted from the 3D segmented image of the PDAC were used for correlation with the presence of liver metastases on CT, age, CA19-9 level and grade of the PDAC (Pearson correlation coefficient).

**Results:** There was significant correlation (p=0,002) between mean iodine density of the whole tumour and the presence of liver metastases (lower iodine density in tumours with liver metastases). The area under the curve (AUC) of mean iodine density in distinguishing tumours with liver metastases from the other tumours was 0,775 (95% CI 0,477-1,073) with a sensitivity of 91,7% and specificity of 80% for cut-off value 0,66 mg/ml. There was no statistically significant correlation with other monitored parameters. Intraclass correlation of segmented volume and iodine densities of PDAC showed perfect agreement (0,974 and 0,960).

**Conclusion:** These pilot data indicate the potential importance of iodine densities in selecting patients with more aggressive PDAC.

### SS 1.10

### Post-surgical outcomes according to National Comprehensive Cancer Network criteria for resection following neoadjuvant therapy in patients with localized pancreatic cancer

J.K. Jang, J.H. Byun, S.J. Choi; *Seoul/KR*

**Purpose:** To assess the prognostic value of the National Comprehensive Cancer Network (NCCN) criteria for resection following neoadjuvant therapy in patients with localized pancreatic ductal adenocarcinoma (PDAC).

**Material and Methods:** This retrospective single-center study assessed 193 consecutive patients with localized PDAC (male:female, 104:89; mean age ± standard deviation, 61.1 ± 9.4 years) who underwent neoadjuvant therapy followed by surgery between January 2010 and March 2021. Combined resectability and carbohydrate antigen (CA 19-9) evaluation before and after neoadjuvant therapy was used to determine whether patients were eligible for resection according to NCCN criteria. Post-surgical overall survival (OS), recurrence-free survival (RFS), and pathologic results were evaluated and compared between patients considered eligible according to the NCCN criteria and those considered ineligible. Preoperative factors associated with better OS and RFS were also investigated.

**Results:** Of 193 patients, 168 (87.0%) were eligible for resection according to the NCCN criteria. Patients eligible according to the NCCN criteria showed marginally longer OS than those considered ineligible (p=.056). After adjustment of variables, meeting the NCCN criteria for resection was an independent predictor of better OS (HR, 0.57; 95% CI, 0.34–0.96, p=.034). RFS was similar between the two groups. Lower T-staging (T2 or less) and less lymphovascular invasion and peri-neural invasion were noted in patients meeting the NCCN criteria (p≤.045).

**Conclusion:** Patients eligible for resection according to the NCCN criteria showed prolonged overall survival and favorable pathological results compared with patients considered ineligible for resection.

**09:00 - 10:30** **Room J2**

*Scientific Session SS 2*

### Interventional Radiology

### SS 2.1

### Multicenter retrospective study of major bleeding in percutaneous hepatic procedures in cirrhotic and non-cirrhotic patients: Relationship with needle gauge and number of needle passes

M.J. Moreno-Rojas, C. Pérez-Serrano, E. Rivas, A. Radosevic, S. Ruiz-Osuna, A. Blasi, Á. García-Criado; *Barcelona/ES*

**Purpose:** The objective of this study is to investigate the frequency of major bleeding in patients with and without cirrhosis who have undergone percutaneous liver procedures (PLP). Additionally, the study aims to explore the relationship between the occurrence of major bleeding and both the gauge of the needle used and the number of needle insertions.

**Material and Methods:** This retrospective study included patients who had scheduled percutaneous liver biopsies (PLB) or percutaneous liver tumor ablations (PLTA) at three centers in Spain. Patients with antithrombotic medications were excluded. Before the procedure, blood tests, including standard coagulation assays, were performed. We recorded demographic, clinical, and technical procedure data. Patients with and without cirrhosis were analyzed separately. The primary outcome was major bleeding.

**Results:** 1797 patients [(1481 without cirrhosis (82%) and 316 with cirrhosis (18%)] that underwent PLP were included. A total of 14 patients (0.8%) experienced major bleeding after the procedure, with no differences between patients with/without cirrhosis; 2 (0.6%) vs 12 (0.8%), respectively (p=1). All bleedings in non-cirrhotic patients were caused by PLB, whereas in cirrhotic patients one case was caused by PLB and another by PLTA. Bleeding was unrelated to the needle gauge in both non-cirrhotic (p = 0.67) and cirrhotic patients (p = 0.48). Similarly, the number of attempts showed no association with major bleeding in either group (p = 1).

**Conclusion:** In this cohort study, major bleeding after a PLP was less than 1%, with no significant difference between cirrhotic and non-cirrhotic patients. Neither the number of passes nor the needle gauge was associated with increased bleeding.

### SS 2.2

### Involution dynamics following microwave ablation of liver tumours

W. Weston[1], O. White[1], N. Goldberg[2], J. Shur[1], E. Johnston[1]; *[1]London/UK, [2]Jerusalem/IL*

**Purpose:** To characterise involution of ablation zone (AZ) volumes over time following microwave liver ablation (MWA), and to assess for a relationship with local tumour progression (LTP).

**Material and Methods:** This retrospective observational study included 55 patients (33M, median age 62 years, range 34–84) who underwent MWA of 88 liver tumours. Median follow-up was 304 days (range 21–741). 498 manual ablation zone segmentations were performed by a single radiologist using Mint Lesion™ (Heidelberg, DE) on intra-procedural contrast-enhanced CT (CECT) (baseline) scans and subsequently on follow-up diagnostic scans at all available time points (median 5, range 1–9) or until LTP occurred. LTP was defined as new tumour contacting the AZ as determined independently by three radiologists. AZ volume change versus baseline was calculated and plotted over time. Non-linear regression methods, including exponential models, were computed using MATLAB (Mathworks, Natick, USA) to describe AZ involution. Fit equations for two groups (LTP vs no LTP) were compared using an F test.

**Results:** Ablation zone involution was best modelled by mono-exponential decay (adjusted R-square= 0.84, p<0.0005). Ablation zones shrank to approximately 30% of original volume with a 151 day 'half-life' (95% confidence interval: 132–172 days), i.e., >90% of involution occurred by 1 year. No statistically significant difference in volume change was found with or without LTP (F=0.93, p=0.78).

**Conclusion:** Body clearance of microwave liver ablation coagulation zones is independent of LTP and closely follows a mono-exponential decay lasting approximately 1 year. These data might improve 3D image registration-based ablation confirmation algorithms at delayed time points.

### SS 2.3

### Outcome mapping study of trials of transarterial chemoembolization for treating hepatocellular carcinoma: A few concepts measured in too many ways

J. Grégory[1], I. Boutron[2], P. Créquit[2], V. Vilgrain[1], M. Ronot[1]; *[1]Clichy/FR, [2]Paris/FR*

**Purpose:** Randomized controlled trials (RCTs) are critical for identifying the best treatment strategy. This study aimed to identify and map outcomes reported in registered and published RCTs of transarterial chemoembolization (TACE) for HCC.
**Material and Methods:** A systematic search was conducted in January 2022 on MEDLINE, EMBASE, and online registries to identify all published or registered RCTs evaluating the efficacy and/or safety of TACE for HCC. Data were collected on the frequency and characteristics of the reported outcome domains and measures.
**Results:** A total of 221 RCTs were identified, including 106 unpublished trials, with a median of 4 [interquartile range, 3–8] outcomes per trial. The number of unique outcome definitions (exclusive of time frame) was 357, distributed among 11 different outcome domains. The most frequently reported domains were survival (78%, 278/357), tumor response (74%, 264/357), and adverse events (68%, 243/357). Among the 357 outcomes, “overall survival” appeared most frequently (47%, 168/357), while 30% of outcomes were evaluated in only one trial. Heterogeneity was found in the definition of outcomes, with 122 measures concerning tumor response, 61 for progression, and 25 for recurrence. Only 4% of outcomes (14/357) were related to quality of life.
**Conclusion:** The study identified a broad range of outcomes reported in RCTs of TACE for HCC. These findings highlight the need for standardization of outcome reporting in HCC trials to facilitate comparison and synthesis of evidence across studies.

### SS 2.4

### Beyond the LEGACY study: Yttrium-90 radioembolization for solitary HCC over 8 cm. A retrospective single center analysis

G. Gullo[1], A. Vit[1], J. Franklin[2], D. Bitetto[1], V. Ferrara[1], M. Sponza[1]; *[1]Udine/IT, [2]Bournemouth/UK*

**Purpose:** Yttrium-90 (Y90) transarterial radioembolization (TARE) is a safe and effective treatment for patients with solitary, unresectable HCC. Following the LEGACY study, TARE is offered for patients with HCC <8cm, but there are limited data in similar cohorts with larger HCC. We assessed the safety and efficacy of TARE in HCC treated in our institution, including patients with HCC >8cm.
**Material and Methods:** This was a retrospective single-center cohort study of all patients with solitary HCC treated with TARE in the period 2005–2022, Child–Pugh A and ECOG performance status 0–1. Primary outcome measures were the objective response rate (ORR) and duration of response (DoR) based on modified Response Evaluation Criteria in Solid Tumors (mRECIST) evaluation. Secondarily, we analyzed the overall survival (OS) and progression-free survival (PFS). We performed a subgroup comparison of lesions <8cm and >8cm**.**
**Results:** 59 patients were included in the study cohort, 50/59 (84.7%) male, with median age of 71. 34/59 (57.6%) HCC were >8cm (median 9 cm) and 25/59 (42.4%) were <8cm (median 5.6cm). No major adverse events were reported. DoR and ORR were 4.4 months (2.0–9.9) and 44.1% (27.2–62.9) for HCC >8cm vs 13.9 months (2.9–20.0) and 73.9% (51.6–89.8) for HCC <8cm (both p=0.032). There were no significant differences demonstrated in OS or PFS.
**Conclusion:** Although treatment responses for patients with tumors >8cm were lower than that for smaller lesions in our cohort and in the LEGACY cohort (median HCC size 2.7cm), a significant proportion of larger HCC still demonstrated a durable treatment response. The benefit of Y90 TARE in larger HCC should be evaluated in prospective trials.

### SS 2.6

### Long-term oncological results of percutaneous radiofrequency ablation for intrahepatic cholangiocarcinoma: A multicentric retrospective study

O. Sutter[1], C. Alitti[1], A. Rode[2], H. Trillaud[3], P. Merle[2], J.-F. Blanc[3], L. Blaise[1], A. Demory[1], G. N’Kontchou[1], V. Grando[1], M. Ziol[1], P. Nahon[1], N. Ganne-Carrié[1], A. Petit[1], O. Seror[1], J.-C. Nault[1]; *[1]Bobigny/FR, [2]Lyon/FR, [3]Bordeaux/FR*

**Purpose:** The effectiveness of radiofrequency ablation (RFA) in intrahepatic cholangiocarcinomas (iCCA) remains insufficiently studied.
**Material and Methods:** Patients with histologically proven iCCA within Milan criteria treated by percutaneous RFA between 2000 and 2022 in three tertiary centers were included. Primary outcome was overall survival in treatment-naive patients and secondary outcomes included ablation completeness, adverse events, local and distant recurrence. 494 patients with HCC on cirrhosis treated by RFA were included as a comparison group.
**Results:** 71 patients, mostly cirrhotic (80%) with a median size of the main tumor of 24mm were included. Complete ablation was achieved in 93% of cases. Local recurrence was 45% at 5 years, lower in multibipolar- versus monopolar-RFA (22% vs. 55%). Both tumor size (P=0.01) and monopolar RFA (P=0.03) were independently associated with a higher local tumor recurrence. In treatment-naive patients (n=45), median overall and recurrence-free survivals were 26 and 11 months. Tumor size (P=0.01) and Child–Pugh B (P=0.001) were associated with death. The rate of distant recurrences was 59% at 5 years, significantly lower for single tumors <2cm (P=0.002) or <3cm (P=0.02). In cirrhotic patients naïve of previous treatments (n=40), overall survival was shorter in iCCA than in HCC (26 vs. 68 months), with more local recurrences (P<0.0001). Among distant recurrences, 50% were extrahepatic metastases compared to 12% in HCC (P<0.001).
**Conclusion:** Multibipolar-RFA provides better results in terms of local tumor control than monopolar-RFA and could be used to treat small iCCA (<3cm). Adjuvant chemotherapy should be discussed due to the frequent extrahepatic metastasis at recurrence.

### SS 2.7

### Percutaneous cryoablation to control hepatic oligoprogression of advanced pancreatic neuroendocrine tumors

R. De Robertis, M. Todesco, E. Bardhi, S. Cingarlini, M. Davì, L. Landoni, M. D’Onofrio; *Verona/IT*

**Purpose:** To describe early experience with percutaneous cryoablation to control hepatic oligoprogression in patients with advanced pancreatic neuroendocrine tumors (pNETs).
**Material and Methods:** Patients with hepatic oligoprogression and otherwise stable disease who underwent percutaneous cryoablation between September 2022 and September 2023 were included. The procedures were performed under local anesthesia; lesion targeting was performed under US guidance; CT was used for procedure monitoring. Treatment response was assessed with 1-month contrast-enhanced CT; further follow-up was performed with contrast-enhanced MRI. The following endpoints were evaluated: technical success, defined as tumor coverage assessed by intraprocedural CT; procedure effectiveness at 1 month; follow-up findings; complications; and mortality.
**Results:** Thirteen lesions with a mean size of 15±6 mm were treated in 10 patients (8 men, 2 women; mean age 53±8 years). The mean follow-up length was 4±3.8 months. Technical success was obtained in all cases. The 1-month effectiveness rate was 69.2%. No local tumor progression was found over a follow-up of 2.3±2.5 months among completely ablated lesions (9/13). Among incompletely ablated lesions (4/13), tumor debulking was obtained in all cases (mean tumor size reduction, 26±11%); over a follow-up of 7,9±3,5 months, local tumor progression occurred in 3 lesions (tumor size compared to baseline, +29±4%), while 1 lesion had sustained tumor reduction compared to baseline (tumor size, −29%). One patient had post-procedural bleeding that resolved with transcatheter embolization. No deaths were found during follow-up.
**Conclusion:** Percutaneous cryoablation is a feasible, safe, and promising procedure to control hepatic oligoprogression of advanced pNETs; longer follow-up time is necessary to establish the effectiveness of this procedure.

### SS 2.8

### Evaluation of tissue effects of radiofrequency ablation on solid tumor: 7T MRI with histological analysis on murine model

M. Todesco, E. Bardhi, F. Spoto, F. Pasquazzo, R. De Robertis, M. D'Onofrio; *Verona/IT*

**Purpose:** To analyze neoplastic tissue adjacent to radiofrequency thermal ablation (RFA) areas to demonstrate early perfusion anomalies and to suggest the synergic role of ablative techniques with chemotherapy treatments.

**Material and Methods:** In this prospective study, a total of 10 mice were subjected to a colon-carcinoma cell implantation and after the tumor colonization, the RFA procedure was performed. All mice underwent a 7T MRI including 2D single-echo single-shot dynamic-contrast-enhanced (DCE) sequences with intravenous injection of gadolinium-based contrast agent (CA), before and after 72 hours from RFA treatment. Data-driven segmentation of the tumor mass enabled the identification of three ROIs with peculiar pharmacokinetic profiles of CA uptake: (i) VTA, viable tumor area; (ii) PNA, partial necrotic area; (iii) CNA, complete necrotic area. Within these ROIs, permeability (K-trans and extravascular-extracellular volume fraction, Ve) and perfusion (time to peak, TTP) indices were evaluated with non-parametric paired samples Wilcoxon tests.

**Results:** Robust increase of Ve (PNA and VTA, p=0.014, p<0.001, respectively) and TTP (VTA, p<0.01) is consistent with severe cellular and vascular damage induced by RFA.

**Conclusion:** Perfusion MRI enables robust detection of significant modulation of the microvasculature within 72 hours from radiofrequency-induced ablation in a murine model of colon carcinoma. This opens a potentially new time window for the evaluation of therapeutic effects of RFA-enhanced chemotherapy.

### SS 2.9

### Adding short to left gastric arterial embolization for the treatment of obesity: Safety and effectiveness

R. Di Giuseppe[1], B. Hansel[2], J. Puyraimond-Zemmour[2], V. Vilgrain[1], M. Ronot[1], L. Garzelli[1]; [1]*Clichy/FR,* [2]*Paris/FR*

**Purpose:** Left gastric artery (LGA) embolization has been found to be an effective and safe minimally invasive procedure to achieve sustainable weight loss in trials. This study assessed the safety and effectiveness of a technical modification of LGA that involves adding short gastric artery (SGA) embolization to LGA embolization.

**Material and Methods:** This retrospective single-center study analyzed twenty obese subjects (median age of 53.5 (30–73)) who were not eligible for bariatric surgery and underwent bariatric embolization with 300–500 μm microspheres in addition to participating in a lifestyle counseling program between March 2021 and July 2022. Eight patients had LGA+SGA embolization, and twelve had LGA embolization alone. The primary endpoint was total body weight loss (TBWL) at 6 months in the SGA+LGA and the LGA-only cohorts. Safety was assessed, defined as the 30-day adverse events rate according to the SIR classification.

**Results:** The mean 6-month post-embolization TBWL in the SGA+LGA cohort was 7.3 kg (95%CI 2.1–12.4; p=.01). The mean 6-month TBWL in the LGA-only cohort was 4.1 kg (mean difference −3.1 kg ± 2.8; 95%CI (−9.1–2.8); p=.28). The mean 6-month post-embolization TBWL in the entire cohort was 5.3 kg (95%CI 2.2–8.3; p<.01). There was no difference in the percentage of 30-day adverse events or severe adverse events (n=4; one splenic infarction in the SGA+LGA cohort; two edematous acute pancreatitis and one splenic infarction in the LGA cohort). No gastric ulcers were diagnosed.

**Conclusion:** Combined SGA and LGA embolization is a safe and effective technique to treat obesity.

**11:00 - 12:30** **Room J1**

*Scientific Session SS 3*

### Rectal cancer staging

### SS 3.1

### Development and validation of MRI radiomics for predicting lateral pelvic lymph node metastasis in patients with rectal cancer: Comparison with radiologists' performance

S.H. Kim, J. Yoo, W. Chang, B.Y. Hur, J.H. Kim; *Seoul/KR*

**Purpose:** To develop and externally validate an MRI radiomics-based model for predicting lateral pelvic lymph node (LPLN) metastasis in rectal cancer patients who underwent LPLN dissection and to compare with radiologists' diagnostic performance.

**Material and Methods:** A total of 336 patients who underwent rectal cancer surgery including LPLN dissection were recruited from three institutions. Among them, 190 patients comprised the development cohort (DC) and 146 comprised the validation cohort (VC). Radiomics features of LPLN were extracted from T2-weighted images (T2WI) of preoperative MRI. Two radiologists independently reviewed T2W MRI and scored the possibility of metastatic LPLN using a 5-point confidence scale in the DC. The least absolute shrinkage and selection operator regression analysis was conducted to select predictive radiomics and clinical features in the DC. Radiomics and clinical-radiomics models were constructed and externally validated using the VC, and their diagnostic performance was compared with the radiologists' assessments using receiver operating characteristic curve analysis.

**Results:** Metastatic LPLN was histopathologically proven in 35.4% (65/190) and 32.9% (48/146) in the DC and VC, respectively. The radiomics model's performance (area under the curve [AUC]: 0.875) and the clinical-radiomics model's performance (AUC: 0.873) were significantly better than that of the radiologists (AUCs: 0.620 and 0.676) in predicting metastatic LPLN in the DC (Ps<0.05). In both DC and VC, there was no significant difference in AUCs between the radiomics (0.875 and 0.821) and clinical-radiomics models (0.873 and 0.829) (P>0.05).

**Conclusion:** The MRI-based radiomics model outperforms experienced radiologists in predicting LPLN metastasis in rectal cancer patients, offering a non-invasive biomarker for personalized surgical treatment.

### SS 3.2

### Evaluation of MRI characterisation of histopathologically matched lymph nodes and other mesorectal nodal structures in rectal cancer

M. Rutegård[1], L. Blomqvist[2], M. Rutegård[1], J. Axelsson[1], M. Båtsman[1], W. Wu[1], I. Ljuslinder[1], J. Rutegård[1], R. Palmqvist[1], F. Brännström[1], K. Riklund[1]; [1]Umeå/SE, [2]Stockholm/SE

**Purpose:** To evaluate MRI-based morphological and size criteria for malignancy in mesorectal lymph nodes in rectal cancer, including the 2016 ESGAR consensus criteria.

**Material and Methods:** A prospective observational study of rectal cancer patients whose individual lymph nodes were anatomically matched to histopathology. Included were nodes from patients not receiving neoadjuvant treatment and those which remained malignant after such treatment. Using histopathological nodal status as the gold standard, irregular margin, round shape, heterogeneous signal, and nodal short axis size, as well as the ESGAR consensus criteria, were evaluated using logistic regression models.

**Results:** The study included 46 patients (19 with neoadjuvant treatment), comprising 465 matched mesorectal lymph nodes. The strongest associations for prediction of nodal involvement in the univariable model were size ≥5 mm (OR 17.17; 95% CI 3.51–83.96) and a central or speckled heterogeneous signal (OR 27.92; 95% CI 3.22–242.11). Positive associations could still be demonstrated when using the factors included in the ESGAR consensus criteria, although only size ≥5 mm remained statistically significant (multivariable OR 10.13; 95% CI 1.78–57.54). When applying the ESGAR consensus criteria to create a binary variable, the OR of malignant outcome for lesions with a positive ESGAR status was 8.39 (95% CI 2.21–31.9).

**Conclusion:** In this combined cohort of patients undergoing primary surgery and neoadjuvant treatment, the size criterion in the baseline setting still seems to be the most important factor when predicting nodal positivity in rectal cancer staging, whereas a heterogenous signal, in addition, might prove to be influential.

### SS 3.3

### Sigmoid take-off to define recto-sigmoid junction and its impact on tumour classification, staging and management

A. Augustine, A. Chandramohan, A. Lakhani, H. Kanamathareddy; Vellore/IN

**Purpose:** Primary objective of this study was to determine the clinical impact of using sigmoid take off (STO) in the management of rectal cancer. Secondary objective was to evaluate the inter-observer reliability in the identification of STO.

**Material and Methods:** In this IRB-approved retrospective study, staging MRI of patients with low and mid-rectal cancers performed between January 2019 and December 2022 was reviewed. The location of the tumour was reclassified based on STO as defined by D'Souza et al. (2018) and compared with location determined based on distance from anal verge (ESGAR). The interobserver agreement for the location of STO and the location of tumour with respect to STO was studied among four radiologists of varying experience (2–15 years).

**Results:** Out of 134 rectal cancer patients included, STO-based assessment resulted in reclassification of 13.4% (n=18) cases into sigmoid cancer. There was, however, no change in the stage of cancer. Among these 18 patients, there would have been a change in management in 5 patients had the initial assessment been a sigmoid cancer. There was excellent agreement among the radiologists for both, measuring the distance of STO from the anal verge (ICC = 0.883, p<0.001) and for determining location of the tumour based on STO (K=0.82, p <0.001).

**Conclusion:** Use of STO changed location of tumour in 13.4% of high and mid-rectal cancers with likely change in management in one fourths of them. There was excellent agreement among radiologists for determining STO and for identifying tumour location based on STO. STO can be easily adopted in clinical practice.

### SS 3.4

### Influence of experience in preoperative staging of rectal cancer

G. Sussan[1], C. D'Alessandro[1], C. Dal Magro[1], P. Santini[1], J. Zambon Bertoja[1], A. Micelli[1], F. Vernuccio[2], E. Quaia[1], F. Crimì[1]; [1]Padua/IT, [2]Palermo/IT

**Purpose:** Our aim was to determine the diagnostic accuracy of preoperative MRI in the evaluation of pathological staging in rectal cancer.

**Material and Methods:** We retrospectively included 109 patients operated for rectal cancer who underwent preoperative MRI from 2014 to 2020. We analyzed rectal staging on MRI and at pathology. MRI staging included the staging performed by the reporting radiologist and an independent assessment performed by one radiology resident. Diagnostic accuracy of the radiologist and the resident were assessed and compared.

**Results:** Accuracy values of T and N staging and EMVI were similar for the resident and the reporting radiologist, with an inter-reader agreement of 67% for T-staging, 63% for N-staging and 86% from EMVI Overstaging of T-status more commonly occurred for high rectal cancer compared to medium-low rectal cancer for both the resident (0% vs 46%; P = 0.0180) and the radiologist (0% vs 48%; P = 0.0140), while understaging for high rectal cancer vs medium-low rectal cancer was significantly more common for the resident (43% vs 8%; p value: P = 0.0035), but not for the radiologist (14% vs 12%; P = 0.8586). The resident more commonly underestimated the precise N stage compared to the radiologist, while the radiologist more commonly underestimated EMVI status (55% vs 67%).

**Conclusion:** Accuracy of TN staging and EMVI assessment of rectal cancer on MRI is still moderate. Expertise in rectal MRI allows to reduce both overstaging of T-status for high-rectal cancer and understaging of N-status, but did not improve EMVI assessment.

### SS 3.5

### AI-accelerated T2-weighted turbo spin echo imaging of the rectum, image quality and acquisition time compared to standard T2-weighted turbo spin echo imaging

J. Shur[1], O. White[1], F. Castagnoli[1], G. Hopkinson[1], E. Scurr[1], B. Whitcher[1], G. Charles-Edwards[1], J. Winfield[1], D.-M. Koh[2]; [1]London/UK, [2]Sutton/UK

**Purpose:** To compare AI-accelerated T2 turbo spin echo (TSE) image quality and acquisition time with standard TSE for rectal imaging.

**Material and Methods:** Prospective study of 50 patients (median age 63 years, range 33-–89, 29M) with anorectal malignancy and rectum *in situ* underwent AI-accelerated T2-weighted TSE imaging (Deep Resolve, Siemens Healthcare) in addition to standard-of-care T2-weighted TSE. Patients were imaged on MAGNETOM Sola (21/50) and MAGNETOM (29/50) Vida scanners. Sagittal and small-field-of-view images were acquired perpendicular to the rectum with matched slice positions. Two radiologists blinded to acquisition type qualitatively scored 4 features using a 4-point Likert scale: signal-to-noise (SNR), rectal wall sharpness, rectal wall layer conspicuity and overall image quality. Differences between median scores for the AI- and standard series were evaluated using a Wilcoxon-test with significance level p<0.05. Time for image acquisition was measured and image artefacts were recorded.

**Results:** Median time savings of 58% (2:25 min) and 71% (4:11 min) for the sagittal and axial series with AI-accelerated reconstructions. AI-accelerated series exhibited better rectal wall sharpness (Likert 4 versus Likert 3, p<0.05) and overall image quality than the standard series (Likert 4 versus Likert 3, p<0.05). No significant difference in the median cohort scores for SNR, or in the median scores of the 1.5T and 3T datasets for any feature was observed. Artefacts identified in 8/100 (8%) AI-accelerated series and 3/100 (3%) standard series, but these did not impact on rectal visualisation.

**Conclusion:** AI-accelerated T2 TSE imaging of the rectum demonstrates equal or better rectal wall definition and better image quality whilst reducing acquisition time by 58–71%.

### SS 3.6

### Defining the tumor location in rectal cancer - Main variations in practice and their potential impact

F. Goedegebuure, F. Arico, M. Lahaye, M. Maas, G. Beets, R. Beets-Tan, D. Lambregts; *Amsterdam/NL*

**Purpose:** This review summarizes differences in definitions and modalities used in (inter)national clinical and radiological rectal cancer guidelines to define rectal tumor location and how these impact treatment stratification.
**Material and Methods:** Clinical and radiological rectal cancer guidelines from the last 10 years were searched and queried for definitions, landmarks and modalities used to classify tumor location (or 'height'), and its impact on treatment stratification.
**Results:** 18 clinical and six radiological guidelines were included (3 American, 3 European, 18 national). Of the clinical guidelines, 28% recommend a measurement from the anal verge to categorize tumors as low (typically 0–5 cm), middle (5–10 cm), or high (10–15 cm); all recommend MRI and/or endoscopy to define location. Alternative landmarks include the anal margin (recommended by 11%), anorectal junction (recommended by 6% specifically for MRI) and anterior peritoneal reflection (recommended by 11%). In 50% of clinical guidelines, tumor location impacts neoadjuvant treatment stratification (and in 72% surgical planning). In 17% of clinical guidelines, tumor location is not defined or included in the recommendations. 100% of radiological guidelines recommend to measure tumor height from the anorectal junction; 83% recommend the anal verge as an additional (or alternative) landmark. Only 33% of radiological guidelines provide thresholds to classify tumors as low/middle/high.
**Conclusion:** This review shows that there is substantial variation in modalities, definitions and landmarks used to classify rectal tumor location. Considering its impact on treatment planning, this highlights the need for more standardized methods that are better aligned between clinical and radiological guidelines.

### SS 3.7

### Impact of endorectal filling on rectal cancer staging in magnetic resonance imaging (MRI)

G. Sussan[1], C. D'Alessandro[1], A. Micelli[1], P. Santini[1], C. Dal Magro[1], J. Zambon Bertoja[1], F. Crimì[1], E. Quaia[1], F. Vernuccio[2]; [1]*Padua/IT*, [2]*Palermo/IT*

**Purpose:** The use of endorectal filling (EF) for MRI staging of rectal cancer is debated. About one third of the panelists of the 2018 ESGAR guidelines for clinical management of rectal cancer recommend rectal filling, while the majority do not. The aim of our study was to compare the diagnostic accuracy of MRI with and without EF for TN-staging and assessment of extramural vascular invasion (EMVI).
**Material and Methods:** We retrospectively included 210 patients operated for rectal cancer from 2007 and 2020 who underwent a clinical MRI of the rectum for preoperative staging. The study cohort was divided into patients who received EF before the pre-operative MRI (EF-MRI group) and those who did not (non-EF-MRI group). Using pathology as reference standard, we compared the accuracy of preoperative MRI, with and without the EF, for T-staging, N-staging and EMVI.
**Results:** The accuracy of MRI for T- and N-staging was similar in the EF-MRI group and the non-EF-MRI group (99/210 patients, 47.1%; AUC 0.43 for T- and AUC: 0.53 for N-staging vs 111/210 patients, 52.9%; AUC 0.44 for T- and 0.52 for N-staging, respectively). With regard to EMVI, both EF-MRI and non-EF-MRI groups achieved high sensitivity (0.99 vs 1), but the proportion of correctly classified patients was higher in the EF-MRI group compared to non-EF-MRI (0.84 [95%CI: 0.75–0.90] vs 0.71 [95%CI: 0.62–0.79]).
**Conclusion:** The use of endorectal filling improves the assessment of extramural vascular invasion in rectal cancer staging.

### SS 3.8

### Accuracy of endorectal ultrasound in preoperative staging of rectal cancer in a large colorectal cancer centre

S. Kamaledeen, M. Dinsey, J. Cowen, C. Ball, R. Beable; *Portsmouth/UK*

**Purpose:** Correct imaging staging is essential in determining treatment strategy for rectal cancer. We aimed to assess the preoperative staging accuracy of endorectal US (ERUS) in rectal cancers operated on at our large colorectal surgery centre.
**Material and Methods:** Retrospective review of all ERUS performed between 2016 and 2022 yielded 136 patients. Included were all those diagnosed with rectal cancer, who were preoperatively staged with both MRI and ERUS and did not require neoadjuvant chemoradiotherapy prior to open or endoscopic surgical excision (n=26). We analysed MRI and ERUS preoperative T stage, circumferential resection margin (CRM) status and histopathology. Sensitivity and positive predictive value (PPV) were calculated with over-staging considered a false positive, and under-staging a false negative.
**Results:** There was no statistical significance in concordance of MRI and ERUS with postoperative histopathology (61.5% vs 58.3%) (p=1.00, CI −25.71% to 17.38%). Sensitivity (64% vs 61%) and PPV (94% vs 93%) for correct T staging was slightly higher with ERUS. There was no significant difference between ERUS and MRI (83.3% vs 75%) (p=0.25, CI −0.55% to 33.88%) in assessment of CRM status. MRI had higher sensitivity for CRM assessment (95% vs 88%) but PPV of accurate CRM assessment was higher in ERUS (94% vs 82%).
**Conclusion:** There was no significant difference between MRI and ERUS in correct T staging of rectal tumours with comparable sensitivity and PPV. MRI may pose less risk of under-staging but greater risk of over-staging of the CRM status compared to ERUS. No significant difference was seen with correct assessment of CRM between the two modalities.

### SS 3.9

### A subregion histogram analysis based on amide proton transfer-weighted MRI for predicting tumor budding grade in rectal adenocarcinoma

P. Xie, Q. Huang, L. Zheng, X. Meng; *Guangzhou/CN*

**Purpose:** To explore the feasibility of predicting the tumor budding (TB) grade in patients with rectal adenocarcinoma based on the heterogeneous subregional histogram features obtained from amide proton transfer-weighted (APTw) MRI.
**Material and Methods:** Patients diagnosed with rectal adenocarcinoma between July 2022 and March 2023 were included in this study. All subjects underwent multi-parameter MRI examination before surgery, including APTw MRI and diffusion-weighted imaging (DWI). Hematoxylin–eosin (H&E) staining was used for TB counting and scoring. Sub-regions were obtained by performing K-means clustering on APTw and apparent diffusion coefficient (ADC) images, and the sub-region histogram features were extracted and filtered using stepwise regression.
**Results:** A total of 74 patients, 49 males and 25 females, with a mean age of 58 years ± 8 years, were included in this study. When K was 5, the prediction model for estimating the TB degree showed the best performance at all aspects compared to K=4 or K=6; When K=5, there were statistical differences in multiple features between Bd1+2 group and Bd3 group in the two sub-regions (sub-regions 3 and 4) with higher APT values. Receiver operating characteristic analysis and internal validation further suggested that the prediction model was the highest when K was 5, with AUC, sensitivity, specificity, and kappa values of 0.92, 93%, 71%, 87%, and 0.65, respectively. There was no significant difference in the histogram features of each sub-region in ADC images.
**Conclusion:** Heterogeneous sub-regional histogram features based on APTw MRI images help predict the TB grade of rectal cancer and are more effective than ADC maps.

### SS 3.10

### It takes two to target: A collaborative effort between a radiologist and a radiation oncologist in delineating gross tumour volumes in rectal cancer

I. Stratulat, L. Blomqvist, A. Valdman*; Stockholm/SE*

**Purpose:** To assess interobserver agreement in gross tumour volume (GTV) delineation between radiologist and radiation oncologist for quality assurance purposes and to compare tumour length assessed at baseline diagnostic (dMRI) and pre-treatment planning (pMRI).

**Material and Methods:** The first 30 patients included in a randomized clinical trial PRORECT (NCT04525989) were selected for the study. GTV was prospectively delineated by a senior radiation oncologist and retrospectively delineated by a radiologist (GTVr). Tumour length measurements were performed retrospectively on dMRI and pMRI by a radiologist and compared with paired samples T test. Interobserver agreement was calculated with intraclass correlation coefficient (ICC), Dice similarity index, Jaccard coefficient and average Hausdorff distance.

**Results:** Patients underwent dMRI and pMRI with medial interval of 21 (15–23) days. The mean GTV and GTVr volumes were 49.3 (±30.1) $cm^3$ and 35.7 (±26.4) $cm^3$, respectively. GTV and GTVr were highly correlated (r=0.926, p<0.001) and the mean volume difference of 13.6 $cm^3$ (±11.4) was significant (p<0.001). Mean Dice score was 0.7 (±0.2). An excellent degree of reliability was found between two readers (average ICC 0.904 (0.22–0.86), F=23.5, p<.001). Tumour length measurements at two time points were perfectly correlated (r=0.96, p<0.001) and the mean difference of 1.77 (±5.15) mm was significant (paired samples test, p<0.035).

**Conclusion:** Overall, interobserver agreement in GTV delineation between a radiologist and a radiation oncologist was good, thus confirming the quality of volume definitions and tumour length estimations within the study. There was a small but significant increase in tumor length during the period between dMRI and pMRI.

**11:00 - 12:30** **Room J2**

*Scientific Session SS 4*

### Hepatocellular carcinoma: Detection and assessment / Gallbladder cancer

### SS 4.1

### Real-world application of noncontrast-abbreviated magnetic resonance imaging in hepatocellular carcinoma surveillance: A retrospective observational study of 1853 at-risk patients

H.K. Yang, S. Lee, M.Y. Lee*; Seoul/KR*

**Purpose:** To assess the applicability of noncontrast-abbreviated MRI (NC-AMRI) as a screening tool for HCC focusing on early-stage detection and false referral in a large real-world surveillance population.

**Material and Methods:** We included consecutive Child–Pugh class A or B adults with chronic hepatitis B or any-cause-cirrhosis under HCC surveillance who underwent at least one NC-AMRI screening between December 2018 and August 2022 at a tertiary referral hospital. A positive result of NC-AMRI was defined as an observation ≥ 10 mm showing either diffusion restriction, mild-to-moderate T2 hyperintensity, or suspicion of a tumor in vein. Early and very early-stage detection, false referral, and receipt of curative treatment were evaluated.

**Results:** A total of 1853 patients (mean age: 58.8 years; 56.4% male) were investigated. Prior to NC-AMRI, 94.4% (1750/1853) of the patients had undergone US screening. 26.5% (463/1750), 52.1% (912/1750), and 21.4% (375/1750) were scored with US liver imaging reporting and data system visualization scores A, B, and C, respectively. 3.3% (61/1853) of the patients developed a total of 68 HCCs. Using NC-AMRI, early- and very early-stage HCC detection rates were 3.1% (58/1853) and 2.3% (43/1853), respectively. The proportions of early- and very early-stage detection were 95.1% (58/61) and 70.5% (43/61), respectively. The false referral rate was 0.5% (9/1853). The proportion of false referrals was 12.9% (9/70). Receipt of curative treatment was 67.2% (41/61).

**Conclusion:** In this real-world surveillance, where US visualization was suboptimal for a majority of the population, NC-AMRI effectively enabled early- and very early-stage HCC detection and subsequent curative treatment.

### SS 4.2

### A multi-site study on the prediction of hepatocellular carcinoma in patients with chronic hepatitis B using heterogeneous data: Expanding the preliminary findings

J.-M. Kim, D. Lee, H.-J. Chung, D. Lee, J.-H. Lee, S. Park*; Seoul/KR*

**Purpose:** To investigate whether CT-derived quantitative factors with deep learning technology is appropriate to improve the performance of HCC prediction in patient with chronic hepatitis B.

**Material and Methods:** We developed HCC prediction model using gradient-boosting machine algorithm in chronic hepatitis B patients. We developed our models by utilizing the 10 parameters employed in the PLAN-B model (cirrhosis, age, platelet count, ETV/TDF, sex, serum ALT, HBV DNA, albumin and bilirubin levels, and HbeAg status), along with CT-derived quantitative factors, specifically liver and spleen volumes, using the commercial body composition analyzer software DeepFore v1.x (Medical IP, South Korea). For the expanded multi-site study, the training dataset comprised 3,379 patients from two institutions, while the external validation set included 4,421 patients from four different institutions. Model performance was compared using the area under the receiver operating characteristics (AUROC).

**Results:** For the expanded multi-site study, DeepFore demonstrated a concordance index (c-index) of 0.82, compared to 0.79 for PLAN-B and 0.76 for PAGE-B.

**Conclusion:** In this study, we developed and evaluated two HCC prediction models. The external validation across multiple sites demonstrated that the model achieved a level of performance suitable for practical clinical application. This robust validation underscores the potential of the model for widespread use in the clinical management of chronic hepatitis B patients, providing a valuable tool for early detection and intervention in HCC.

### SS 4.3

### A multi-centre comparative analysis: Optimal contrast phase acquisitions on CT liver and MRI liver in the assessment of hepatocellular carcinoma (HCC)

N. Davendralingam; *London/UK*

**Purpose:** Objective: to review quality of contrast phase acquisitions on CT liver and MRI liver for the assessment of HCC. Standard: LI-RADS technical requirements for CT, MRI and contrast-enhanced US. Target: 100% compliance.
**Material and Methods:** Random selection of 5 CT liver and MRI liver, respectively, for the assessment of HCC is obtained from multiple different institutions throughout the United Kingdom retrieved from the PACS system of a teleradiology platform. Standards: For CT, there should be minimum 50HU enhancement (liver parenchyma). For both CT and MRI, should have adequate late arterial phase. For CT, delayed phase is mandatory. For MRI, subtraction imaging is recommended.
**Results:** Preliminary assessment of 8 institutions suggest poor compliance (less than 60%) to expected standards of expected contrast phase acquisition in both CT and MRI liver in which there is poor arterial timing (too early) and missing delayed phase, particularly on CT. Study is ongoing with final results expected in April.
**Conclusion:** There is poor compliance particularly in regional centres to optimise contract phase acquisition in CT and MRI liver, which can result in missed HCCs which is a serious breach of patient care. Mandatory national guidelines should be created with regular local audits expected of local hospitals to ensure adherence to expected standards.

### SS 4.5

### Added value of a combination of positron emission tomography with 18F-FDG and 18 F-fluorocholine for staging optimization and treatment modification in patients with hepatocellular carcinoma: The prospective multicentric PET HCC01 study

J.-C. Nault[1], M. Boubaya[1], M. Wartski[1], A. Dohan[2], S. Pol[2], G. Pop[1], M. Soussan[1], O. Seror[1], C. Costentin[3], J. Roux[3], C. Sengel[3], M. Lequoy[2], F. Montravers[2], Y. Menu[2], G.-P. Pageaux[4], D. Mariano Goulart[4], B. Guiu[4], A. Luciani[5], M. Dioguardi Burgio[6], M. Wagner[2], P. Maksud[2], S. Mule[1], M. Allaire[2], S. Sidali[6], A. Coilly[7], F. Besson[8], M. Lewin[7], H. Regnault[5], C. Hollande[6], G. Amaddeo[5], M. Ronot[6], N. Ganne-Carrié[1], V. Levy[1], R. Lebtahi[6], J. Chalaye[5], M. Bouattour[2]; *[1]Bobigny/FR, [2]Paris/FR, [3]Grenoble/FR, [4]Montpellier/FR, [5]Créteil/FR, [6]Clichy/FR, [7]Villejuif/FR, [8]Bicetre/FR*

**Purpose:** The role of positron emission tomography (PET)–CT with 18F-fluorodeoxyglucose (FDG) and 18F-fluorocholine in staging of HCC has never been prospectively assessed.
**Material and Methods:** We conducted a prospective study in nine centers, including patients with a first diagnosis of HCC BCLC A to C (without metastasis) (NCT04391348). Patients underwent 18F-FDG-PET–CT and 18F-fluorocholine-PET–CT, liver MRI, and chest/hepatic CT within 4 weeks. A first tumor staging and treatment decision were performed in tumor board using morphological imaging, blind to the results of the PET–CTs. The results of the PET–CTs were revealed and a second tumor staging and treatment decision recorded. The primary endpoint was the proportion of modification of treatment planned due to the PET–CT.
**Results:** A total of 230 patients were included, 215 were analyzable. The median age was 65.7, 89.8% male, and 72% cirrhotic. Using morphological imaging, HCC was classified BCLC stage A (65.1%), stage B (22.8%), and stage C (12.1%). New lesions were identified in 20 patients (9.3%) by PET–CTs (16 extrahepatic lesions and five intrahepatic lesions: eight identified by both tracers, six by 18F-FDG, and seven by 18F-fluorocholine). The PET–CTs modified the staging in 10 patients (4.7%): from BCLC A to B (n=2), BCLC A to C (n=2), BCLC B to C (n=2), and BCLC C without metastasis to BCLC C with metastasis (n=4). Modification of the planned treatment was met in four patients (2.9%): selective internal radiation therapy (SIRT)+atezolizumab/bevacizumab to atezolizumab/bevacizumab only (n=1), resection to atezolizumab/bevacizumab (n=1) or to SIRT (n=1), and TACE to atezolizumab/bevacizumab (n=1).
**Conclusion:** 18F-FDG and 18F-fluorocholine-PET–CTs have a limited value for staging in patients with a first diagnosis of HCC.

### SS 4.6

### Evaluating the diagnostic efficacy of simultaneous multi-slice-accelerated intravoxel incoherent motion and diffusion kurtosis imaging in HCC: Predicting microvascular invasion, histologic grade, and early recurrence risk

Y. Wu, Z. Ye, B. Song; *Chengdu/CN*

**Purpose:** To evaluate the diagnostic efficacy of simultaneous multi-slice (SMS)-accelerated intravoxel incoherent motion (IVIM) and diffusion kurtosis imaging (DKI) compared to conventional models in predicting microvascular invasion (MVI) and histologic grade in HCC.
**Material and Methods:** Approved by the Institutional Review Board, this study enrolled 40 HCC patients from June 2021 to November 2022. Preoperative MRI included DKI and IVIM protocols. Diffusion parameters such as apparent diffusion coefficient (ADC), true diffusion coefficient (D), perfusion-related diffusion (D*), perfusion fraction (f), mean kurtosis (MK), and mean diffusivity (MD) were analyzed. Receiver operating characteristic curve analyses evaluated diagnostic efficiency for MVI detection and high-grade HCC prediction. Logistic regressions assessed clinical, laboratory, and diffusion factors for early recurrence risk (≤1 year).
**Results:** The acquisition times for SMS-DWI, SMS-DKI, and SMS-IVIM were reduced by 37%, 44%, and 31%, respectively, compared to conventional sequences. In distinguishing MVI-positive from MVI-negative HCC, SMS-D exhibited an area under curve (AUC) of 0.84, while CON-D achieved a sensitivity of 0.97. Significant differences were observed in D and MD values from both diffusion-weighted sequences and in MK values from SMS sequences between high-grade and low-grade HCCs (P=0.00–0.04), demonstrating moderate diagnostic efficacy (AUC=0.71–0.80). Higher MK (OR =4.514, P=0.002) and lower D (OR =5.483, P=0.007) were identified as independent risk factors for early recurrence in HCC.
**Conclusion:** SMS significantly reduced MRI scan times while maintaining reliable diffusion metrics. IVIM-derived D and DKI-derived MK outperformed ADC in predicting MVI and the histologic grade of HCC, and are associated with an increased risk of early tumor recurrence.

### SS 4.7

### Prognostic implications of MRI-assessed intratumoral fat in hepatocellular carcinoma: An international dual-center study

H. Jiang[1], R. Cannella[2], Z. Wu[1], A. Beaufrère[3], M. Dioguardi Burgio[3], R. Sartoris[3], Y. Wang[1], Y. Qin[1], J. Chen[1], Y. Chen[1], W. Chen[1], Y. Shi[1], B. Song[1], M. Ronot[3]; *[1]Chengdu/CN, [2]Palermo/IT, [3]Clichy/FR*

**Purpose:** Intratumoral fat is frequently detected in HCC with reported prognostic value. Therefore, we aimed to investigate the clinical–pathological–radiological correlations and prognostic implications of MRI-assessed intratumoral fat.
**Material and Methods:** This international retrospective cohort study included consecutive adult patients who underwent resection for solitary HCC and preoperative contrast-enhanced MRI from two tertiary-care hospitals in East Asia and Western Europe. MRIs were independently evaluated by three blinded radiologists at each hospital. Based on LI-RADS v2018, intratumoral fat was defined as “fat in mass more than adjacent liver”, and the homogenous subtype as intratumoral fat “in absence of mosaic and nodule-in-nodule architecture”. Recurrence-free survival (RFS) and overall survival (OS) were estimated by the Kaplan–Meier method, and prognostic factors were identified by Cox regression analyses.
**Results:** 933 patients (Asia, $n$=736; Europe, $n$=207) were included. MRI-assessed intratumoral fat was detected in 29.6% and 30.6% of patients from Asia and Europe ($P$=0.719). In both cohorts, the steatohepatitic subtype, non-peripheral “washout”, enhancing “capsule”, and mosaic architecture were more frequently observed in tumors with intratumoral fat ($P$ range, <0.001–0.041). Intratumoral fat in general was non-prognostic ($P$ range, 0.481–0.973). However, in the Asian cohort, homogenous intratumoral fat was associated with longer RFS ($P$<0.001) and OS ($P$<0.001) and remained protective of RFS (HR, 0.60; $P$=0.010) and OS (HR, 0.33; $P$<0.001) at the multivariable Cox analyses.
**Conclusion:** MRI-assessed intratumoral fat was more frequent in steatohepatitic HCC and in general non-prognostic. However, homogenous intratumoral fat was an independent protective factor of both RFS and OS in the Asian cohort.

### SS 4.8

### Comparison of the diagnostic performance of European (ESGAR) vs North American (SRU) 2022 ultrasound gallbladder polyp management guidelines for ≥ 7mm polyps

B.P. Nanda[1], B. Moloney[2], A. Gershon[1], H.-J. Jang[1], K. Elbanna[1], X. Liu[1], K. Nowak[1], K. Khalili[1]; *[1]Toronto, ON/CA, [2]Salthill/IE*

**Purpose:** To compare the diagnostic performance of 2022 ESGAR/EAES/EFISDS/ESGE vs Society of Radiologists in Ultrasound's (SRU) 2022 gallbladder polyp (GBP) management guidelines for polyps of ≥7mm.
**Material and Methods:** All patients who with ≥7mm polyp reported on available US scans at a hepatobiliary center with eventual cholecystectomy over a 20-year period were included. 2–5 images from the earliest available scan were selected for review by 4 blinded abdominal radiologists (3–23 years' experience). Shape (thin stalk/thick stalk/sessile), wall thickening and size were used to classify imaging features and along with relevant clinical features (age, ethnicity, PSC) formed the ESGAR and SRU binary management categories of no follow-up/follow vs refer-to-surgeon. Pathology reports were classified according to WHO 2017 by a staff pathologist, with outcome for management defined as polyps requiring resection (PRR, all neoplasms).
**Results:** 135 patients (mean age 53.3, range 20–86, 59 female (42.8%)) with median polyp size 10mm (range 7–45) formed the study cohort. 27/138 (20.0%) of patients had PRR (5 pyloric gland adenoma, 8 intracholecystic papillary neoplasm, 12 carcinoma in situ/carcinoma, 2 Mets). Intra-observer variability (SRU-risk) kappa values for 4 readers were 0.50/0.52/0.60/0.95 (moderate–almost perfect). Mean kappa for interobserver agreement was 0.37 (range 0.28–0.48, fair–moderate). Mean accuracy/sensitivity/specificity (range) for the detection of polyp requiring resection were ESGAR 50%(47–53%)/90%(85–96)/40%(38–42) and SRU 85%(84–87)/61%(59–63)/90%(89–93). The differences between the mean accuracy/sensitivity/specificity of the two guidelines were significant (p= 0.03, <0.001, <0.001, respectively).
**Conclusion:** Regarding the detection of PRR at presentation, ESGAR management guidelines are significantly more accurate with high sensitivity, while SRU's are more specific resulting in fewer unnecessary recommendations for surgery but more follow-up of PRR.

### SS 4.9

### The significance of contrast enhancement ratio in differentiation of gallbladder cancers from complicated cholecystitis

F. Kulali; *Istanbul/TR*

**Purpose:** Patients with gallbladder cancers have poor prognosis. They can mimic complicated cholecystitis. Pre-operative early and accurate diagnosis is essential. Therefore, our aim was to investigate the significance of contrast enhancement ratio (arterial phase/pre-contrast) on MRI in differential diagnosis of cancer and complicated cholecystitis.
**Material and Methods:** A total of 76 patients who had complicated cholecystitis with a suspicion of gallbladder cancer in pre-operative abdominal MRI reports during a retrospective 48-month period were included in this study. All patients had histopathological results. Imaging features (size, wall thickness, wall irregularity/discontinuity, signal intensities, contrast enhancement ratio and restriction of diffusion) were evaluated. The relationship between MRI and histopathological findings was statistically analyzed.
**Results:** Of 76, 36 (a mean age of 50±13 years, 18 men and 18 women) had only complicated cholecystitis whereas 40 patients (a mean age of 66±14 years, 17 men and 23 women) had complicated cholecystitis with gallbladder cancers. There was no significant difference of wall thickness between groups ($p > 0.05$). Asymmetrical and focal wall thickness was prevalent in malignant group ($p < 0.05$). The contrast enhancement ratio with a cut-off > 1.6 (sensitivity of 81.1%, specificity of 76.2%, accuracy of 79.3%, AUC: 0.939) and apparent diffusion coefficient (ADC) value with a cut-off value of $1 \times 10^{-3}$ $mm^2/s$ (specificity of 85.2%, sensitivity of 74.4%, accuracy of 78.8%, AUC: 0.959) were found significant for malignant group ($p < 0.05$).
**Conclusion:** Detailed MRI evaluation with awareness of high arterial contrast enhancement ratio and lower ADC value which indicate malignancy is important especially in patients with complicated cholecystitis.

**14:30 - 16:00** **Room J2**

*Scientific Session SS 5*

### Crohn's disease

### SS 5.1

### METRIC-EF: Magnetic resonance enterography to predict disabling disease in newly diagnosed Crohn's disease - A multicentre, non-randomised, single arm, prospective study

S. Kumar[1], A. Plumb[1], S. Mallett[1], C. Clarke[1], T. Parry[1], J. Weng[1], G. Bhatnagar[2], S. Bloom[1], J. Hamlin[3], A. Hart[1], S. Travis[4], R. Vega[1], M. Hameed[1], A. Bhagwanani[2], R. Greenhalgh[1], E. Helbren[5], J. Stephenson[6], I. Zealley[7], V.N. Eze[8], J. Franklin[9], A. Corr[1], A. Gupta[1], D. Tolan[3], W. Hogg[3], A. Higginson[10], T. Raine[11], L. Lee[6], R. Pollok[1], J. Patel[1], S. Halligan[1], S.A. Taylor[1], METRIC-EF Study Investigators[1]; *[1]London/UK, [2]Frimley/UK, [3]Leeds/UK, [4]Oxford/UK, [5]Hull/UK, [6]Leicester/UK, [7]Dundee/UK, [8]Kent/UK, [9]Bournemouth/UK, [10]Portsmouth/UK, [11]Cambridge/UK*

**Purpose:** To develop and internally evaluate a multivariable prediction model comprising both clinical predictors and predictors based on MRE to improve prediction of disabling Crohn's disease (CD) within 5 years of diagnosis.
**Material and Methods:** In this multicentre, prospective study across 9 UK hospitals, we assessed the comparative predictive ability of prognostic models incorporating magnetic resonance enterography (MRE) scores [MRE Global Score (MEGS), simplified MAgnetic Resonance Index of Activity (sMaRIA) and Lémann Index] versus models using clinical characteristics alone, e.g. age, smoking, disease location, blood/stool markers, to predict disabling (severe) CD (modified Beaugerie definition) within 5 years of diagnosis.
**Results:** We studied 194 newly diagnosed patients, median age 29, IQR 22 to 44 years, 48% male. Within 5 years from diagnosis, 42% (81/194) of participants developed disabling disease. In the univariable analysis, initial requirement for steroid therapy was associated with a higher risk of developing disabling disease [HR 2.08 (95% CI 1.35–3.23)]. The baseline clinical model had 52% (41–62) sensitivity and 69% (59–76) specificity for predicting the top 40% of patients with the greatest risk of developing disabling disease, and 86% (77–92) sensitivity and 35% (27–45) specificity for predicting patients with an absolute risk of developing disabling disease of greater than or equal to 10%. There was no difference in sensitivity between models incorporating MEGS, sMaRIA and LI compared to the baseline clinical models.
**Conclusion:** Addition of MRE activity/bowel damage scores to a multivariable model comprising existing clinical predictors did not improve prediction of disabling CD.

### SS 5.2

### Quantified motility and structured anatomical scoring in clinical practice - A real world investigation of emerging markers of small bowel Crohn's disease activity

G. Bhatnagar, A. Bhagwanani, A. Menys, I. Naim, T. Shepherd, S. Rahman; *London/UK*

**Purpose:** Quantified small bowel motility has transitioned from being a research tool into the early stages of clinical practice for assessing small bowel Crohn's Disease activity. We compare findings with simplified MAgnetic Resonance Index of Activity (sMARIA) in a group of patients undergoing MR enterography.
**Material and Methods:** A subset of patients with a diagnosis of Crohn's undergoing therapy who had MRE with motility imaging at two interval time points were selected. Motility index derived from 'cine' images using commercially available software (GIQuant®, Motilent, London, UK) was acquired at the site of most severe disease. This was correlated with radiological features used to assess disease severity in Crohn's (sMaRIA). The change in the motility index between interval studies was correlated with the radiological findings categorised as response, stable disease and progression. Partial response and was documented where there was no change in sMaRIA but improved radiological appearances.
**Results:** 23 patients (61% male) median (range) age 32 (14–65) years were identified with a median interval of 469 (133–699) days between MRE scans. There was no correlation between sMaRIA score and motility index at baseline (p=0.81) but a negative correlation between absolute and delta values at follow-up (both p<0.01). There was no significant change in the motility index in patients with clinically stable disease. Patients with stable sMaRIA scores but worsening or improved radiological appearances showed correlating motility indices.
**Conclusion:** Quantified small bowel motility correlates with the radiological features used to assess Crohn's severity. Findings do not entirely agree with structural features (sMARIA) but appear complementary and agree with overall radiological impression.

### SS 5.3

### Getting to the score faster: Impact of region of interest type on quantitative scores for small bowel motility in the clinical setting

G. Bhatnagar[1], I. Naim[2], A. Menys[2]; [1]*Frimley/UK*, [2]*London/UK*

**Purpose:** Quantified small bowel motility, a biomarker for Crohn's Disease (CD), now has clinical applications. Traditionally, GIQuant scores require drawing a polygon region of interest (ROI) around a lesion and transposing it to the motility map. However, many picture archiving and communication systems (PACS) lack this polygon tool, necessitating data transfer to separate readers slowing the process. This study explores using the 'circle' ROI tool, commonly available in PACS, to assess if it yields comparable GIQuant scores.
**Material and Methods:** In 8 CD patients, a radiologist and imaging scientist placed 32 ROIs (one in each quadrant) using both polygon and circular ROIs. This process was repeated at the same place with a circular ROI which touched the serosa either side of the bowel. Summary statistics were collected along with evidence of associations including i) polygon vs circle GIQuant score and ii) impact of ROI size on difference in GIQuant scores.
**Results:** The average polygon GIQuant score was 269.0 (66 to 612) and circle was 269.9 (range 63.6 to 707), difference −0.84, P=0.9. Average polygon area was larger than circles (5.7cm$^3$ vs 4.3cm$^3$) but non-significantly so, difference −1.38, P=0.33. There were no significant correlations between difference in GIQuant score and difference in ROI area. There was a weak, non-significant correlation (R = −0.27) between circle GIQuant scores and size of circle ROI.
**Conclusion:** Circle ROI produced statistically and visually similar GIQuant scores to the previously published polygon ROI score. The circle ROI is widely available on hospital PACS allowing GIQuant measurements to be made in <30s compared to 5–10 minutes where results must be exported to third-party viewers.

### SS 5.4

### Assessing creeping fat in Crohn's disease through MRE: Interobserver agreement and correlation with disease severity

N. Chaniotaki[1], M. Karachaliou[1], A. Karatzas[1], C. Triantos[1], C. Kalogeropoulou[1], S. Gourtsoyianni[2]; [1]*Patras/GR*, [2]*Athens/GR*

**Purpose:** The aim of this retrospective study was to visually assess the volume and distribution of creeping fat (CF), a potential independent factor during Crohn's disease (CD), in MR Enterographies (MREs) and correlate it with simplified MAgnetic Resonance Index of Activity (MARIAs) score.
**Material and Methods:** Reports from 308 (234 conducted in an academic institution from 2019 to 2023 +74 in a private practice from 2014 to 2023) MREs performed in patients with endoscopically suspected or confirmed CD, reviewed by experienced GI Radiologists, were included. Twenty-five exams (23 patients) with reported creeping fat were identified and independently assessed by one experienced radiologist and one in training. CF score (1: mild, 2: moderate, 3: severe) was attributed to each exam by visual assessment. Fat wrapping distribution (FWD) was graded according to proportion of intestinal circumference affected (1: minimal up to 90°, 2: moderate up to 180°, 3: significant up to 360°). Evaluation was based on balanced steady-state gradient echo sequences. Inter-rater reliability was assessed and correlated with MARIAs score.
**Results:** Good interobserver agreement for both CF volume (71%) and fat wrapping distribution (74%) was observed. MARIAs score 2 patients had mostly CF score 1, whereas FWD was mostly significant. With MARIAs score 5, moderate FWD was most prevalent combined with CF score 3. No statistically significant association was found between CF score or FWD and MARIAs score>2 (p=0.155 and p=0.573, respectively).
**Conclusion:** Lack of significant correlation between CF and MARIAs score >2 suggests that CF alone may not act as a definite indicator of degree of CD severity.

### SS 5.5

### Volumetric measurement of terminal ileal Crohn's disease by MR enterography: A feasibility study

S. Kumar[1], N. Rao[2], A. Bhagwanani[3], T. Parry[1], M. Hameed[1], S. Rahman[1], H. Fitzke[1], J. Holmes[1], B. Barrow[1], A. Bard[1], A. Menys[1], D. Bennett[4], S. Mallett[1], S.A. Taylor[1]; [1]*London/UK*, [2]*Coventry/UK*, [3]*Frimley/UK*, [4]*Cambridge, MA/US*

**Purpose:** MR enterography (MRE) interpretation of Crohn's disease (CD) is subjective and uses 2D analysis. We evaluated the feasibility of volumetric measurement of terminal ileal (TI) CD on MRE compared to endoscopy and simplified MAgnetic Resonance Index of Activity (sMARIA), and the responsiveness of volume to biologics.
**Material and Methods:** CD patients with MRE and contemporaneous CD endoscopic index of severity-scored ileocolonoscopy were included. A centre line was placed through the TI lumen defining the diseased bowel length on the T2-weighted non-fat saturated sequence, used by two radiologists to independently segment the bowel wall to measure volume. In phase 2, we measured disease volume in patients treated with biologics, who had undergone pre- and post-treatment MRE, with treatment response classified via global physician assessment.
**Results:** Phase 1 comprised 30 patients {median age 29 [interquartile range (IQR) 24–34] years}. Phase 2 included 12 patients [25 years (22–38) years]. In phase 1, the mean of the radiologist-measured volumes was used for analysis. The median disease volume in those with endoscopically active CD was 20.9cm$^3$ (IQR 11.3–44.0) compared to 5.7cm$^3$ (2.9–9.8) with normal endoscopy. The mean difference in disease volume between the radiologists was 3.0cm$^3$ (limits of agreement −21.8–15.9). The median disease volume of patients with active CD by sMARIA was 15.0cm$^3$ (8.7–44.0) compared to 2.85cm$^3$ (2.6–3.1) for those with inactive CD. Pre- and post-treatment median disease volumes were 28.5cm$^3$ (26.4–31.2) and 11cm$^3$ (4.8–16.6), respectively, in biologic responders versus 26.8cm$^3$ (12.3–48.7) and 40.1cm$^3$ (10–56.7) in non-responders.
**Conclusion:** Volumetric measurement of TI CD by MRE is feasible, related to endoscopy and sMARIA activity, and responsive to biologics.

### SS 5.6

### Diagnostic accuracy of MRI enterocolonography, intestinal US and panenteric capsule endoscopy for assessing treatment response in known Crohn's disease

J. Brodersen[1], S. Rafaelsen[2], M.A. Juel[2], T. Knudsen[1], J. Kjeldsen[3], M.D. Jensen[2]; [1]Esbjerg/DK, [2]Vejle/DK, [3]Odense/DK

**Purpose:** Methods for monitoring Crohn's disease (CD) have improved. Modalities are MRI enterocolonography (MREC), intestinal US (IUS), and panenteric capsule endoscopy (PCE). The aim of this study was to evaluate MREC, IUS and PCE for determining response to treatment.
**Material and Methods:** Patients with active CD were included in this prospective, blinded, multicenter study. Each patient was scheduled for an IC, MREC, IUS, and PCE before and after medical treatment. IC served as gold standard, and endoscopic response was defined as a > 50% reduction of the Simple Endoscopic Score for CD (SES-CD). The definitions of treatment response with MREC were 1) a > 25% reduction of the global Magnetic Resonance Index of Activity (MaRIA), 2) a MaRIA segmental score < 11 in segments with a baseline score > 11, and 3) a > 25% reduction of bowel wall thickness. IUS treatment response was defined as a > 25% reduction of bowel wall thickness.
**Results:** 50 patients completed all examinations. Response rate was 50%. Patients had a mean SES-CD of 12.5 and 7.1 before and after medical treatment (P < 0.001). The MaRIA score decreased from the SUS-CD from 6.2 to 4.0 (P < 0.001), and SES-CD with PCE from 13.2 to 7.8 (P < 0.001). The sensitivity/specificity of MREC, IUS and PCE for detection of response was 65.2%/91.3%, 71.4%/76.5%, and 87.5%/86.7%, respectively.
**Conclusion:** In patients with CD, cross-sectional imaging is inferior to PCE for determining treatment response.

### SS 5.7

### Diagnostic value of MR and CT enterography in post-operative recurrence of Crohn's disease: A systematic review and meta-analysis

M. Chavoshi, S. Zamani, S. Kolahdoozan, A. Radmard; Tehran/IR

**Purpose:** To assess the diagnostic yield of enterography techniques in post-operative recurrence (POR) of Crohn's disease (CD).
**Material and Methods:** A systematic electronic bibliographic databases search was conducted. The inclusion criteria of published original articles were utilized MR enterography (MRE) or CT enterography (CTE) after an ileocolonic resection; documented POR by ileo-colonoscopy; provided crude data of diagnostic performance. The POR was defined as Rutgeerts' score≥i2. A random effect model was used for the analysis of diagnostic performance. The relative risk and diagnostic value of each imaging parameter were calculated.
**Results:** Ten studies (11 populations and 291 patients) were included (4 CTE and 7 MRE with 112 and 168 patients, respectively). The pooled sensitivity and specificity of the enterography were 91% (95% CI: 0.85–0.95) and 75% (95% CI: 0.56–0.87), respectively. CTE's pooled sensitivity and specificity were 93% (95% CI: 0.87–0.96) and 67% (95% CI: 0.35–0.90), respectively. MRE revealed pooled sensitivity and specificity of 90% (95% CI: 0.78–0.96) and 78% (95% CI: 0.57–0.90), respectively. The inter-study heterogeneity was low for sensitivity ($I^2$=29%, p value=0.17) and high for specificity ($I^2$=85%, p value<0.01). Wall enhancement, anastomosis wall thickening, anastomosis stenosis, pre-anastomotic dilatation, penetrating lesion, comb sign, and perivisceral edema were significantly higher in POR patients. While wall thickening was the most sensitive sign (81%), penetrating lesions were the most specific finding (97%).
**Conclusion:** MRE and CTE exhibit high sensitivity and acceptable specificity (especially MRE) for detection of POR in CD which makes them an effective initial screening tool and reserves ileo-colonoscopy for those patients with inconclusive enterography results.

### SS 5.8

### Motility at cine-MRI in stricturing Crohn's disease patients to evaluate stricture composition

K. Beek[1], K. van Rijn[1], C. de Jonge[1], F. de Voogd[1], C. Buskens[1], A. Mookhoek[2], E. Neefjes-Borst[1], K. Horsthuis[1], J. Tielbeek[3], G. D'Haens[1], K. Gecse[1], J. Stoker[1]; [1]Amsterdam/NL, [2]Bern/CH, [3]Haarlem/NL

**Purpose:** Is motility, measured with cine-MRI, capable of distinguishing chronic from inflammatory strictures in Crohn's disease (CD).
**Material and Methods:** Adult CD patients with a small bowel stricture, scheduled for surgical resection, were included. All patients underwent coronal dynamic 2D-balanced fast field echo MRI scans of the (pre-)stricture (dilatation) after fasting and 1.9%-mannitol-preparation. Motility was assessed with a validated displacement mapping technique (GIQuant). Strictures (wall thickening>3mm and ≥50% luminal reduction) and pre-stricture dilatations (luminal diameter>3cm) were delineated to obtain a motility score (arbitrary units=AU). Tissue blocks of the most strictured area were scored by two blinded expert pathologists as [1] inflammatory (significant neutrophilic or lymphocytic/plasmacytic infiltrate), [2] chronic (fibrosis, adipose tissue and/or muscular hypertrophy) or [3] mixed phenotype (inflammatory and chronic aspects). Motility scores of strictures and pre-stricture dilatations are presented as median [interquartile range (IQR)]. Kruskal–Wallis and Mann–Whitney U test were used.
**Results:** 32 strictures (motility 69.8AU [62.4–89.4]) and 10 pre-stricture dilatations (motility 174.0AU [101.9–291.6]) were analyzed (30 patients). Motility in pre-stricture dilatations was higher in chronic (n=5; 254.6AU [174.0–326.2]) compared to inflammatory and mixed strictures (n=5; 108.1AU [71.9–208.1]; p=0.095). No difference was found in motility between inflammatory (n=4; 70.8AU [46.9–80.3]), mixed (n=17; 69.8AU [65.7–88.1]) and chronic strictures (n=12; 69.1AU [61.2–152.7]). Strictures<5 cm (which were all mixed or chronic) had a higher motility in pre-stricture dilatation (n=5; 289.5AU [186.4–330.4]) compared to pre-stricture dilatation of strictures>5 cm (n=5; 108.1AU [72.0–174.0]; p=0.03).
**Conclusion:** Motility in pre-stricture dilatations is higher in both chronic strictures and strictures <5cm. An explanation for this phenomenon could be the increased wall stiffness in strictures with chronicity. Motility in pre-stricture dilatations could serve as a biomarker of stricture composition.

### SS 5.9

### MRI-based average T2 signal as a non-invasive measure of fibrosis in enteric Crohn's disease

I. Naim[1], C. Hoad[2], C. Clarke[2], A. Mukherjee[2], N. Jinnah[2], P. Gowland[2], O. Mougin[2], A. Bard[1], A. Menys[1], G.W. Moran[2]; [1]London/UK, [2]Nottingham/UK

**Purpose:** In Crohn's disease (CD), 10% of patients present with fibrostenosis with a further 10% progressing from inflammatory to a fibrostenotic disease behaviour over 7 years. 40% of fibrostenotic CD patients need surgery at 5 years compared to ~10% with inflammatory disease. T1, T2, diffusion-weighted imaging, motility and bowel volumes have shown promise in the non-invasive measure of fibrosis. This study investigates the potential of MRI measures as non-invasive biomarkers for fibrosis in CD.
**Material and Methods:** CD patients in need of surgical resection attended a one-hour 3T-MRI scanning session within 12 weeks before their intestinal resection surgery. Histological samples were scored by a specialized GI pathologist for inflammation, fibrosis, and muscular hypertrophy. A radiologist with the help of the histopathologist located the strictures on MRI scans. GI-Seg (Motilent, London, UK) is an image analysis tool which was used on T2W scans to measure bowel wall volume and average signal intensity of each stricture.
**Results:** A total of 10 CD patients (20 strictures) were included. Average T2 signal had a significant correlation with global fibrosis scores (r(20) = .563, p = .010) and total inflammation scores (r(20)=.451, p=.046). Additionally, stricture volume showed a fair correlation with chronic inflammation component scores (r(20)=.453, p=.045). Notably, quantified T1 values correlated with total inflammation scores (r(17)=.493, p=.045).
**Conclusion:** The correlations between T2 signal, stricture volume, and inflammation scores suggest MRI measures, particularly those from GI-Seg, as promising biomarkers for fibrosis and inflammation in CD. However, further research is needed to identify markers specifically indicative of fibrosis to differentiate it from inflammation.

### *SS 5.10*

**MRI of complex perianal fistula interpreted with the aid of a rapid 3D interactive model: Initial experience**

G. Bhatnagar, L. Lindsay, L. Carter, I. Naim, K. Williams, J. Huddy*; London/UK*

**Purpose:** Perianal fistulas vary in complexity and MRI may fail to communicate important information owing to the nature of a written report. In this study, we explore a new, rapid markup tool for creating a 3D model of perineal fistula from routine perianal MRI.

**Material and Methods:** Three patients undergoing MRI for perianal fistula were selected for this study to capture different and challenging aspects of fistulising disease. Patient 1 has a trans-sphincteric tract with two internal openings in the upper anal canal. Patient 2 demonstrated an intersphincteric fistula in the left posterior quadrant and a trans-sphincteric fistula that opens in the right posterior quadrant. Patient 3 has an internal opening that gives rise to an anterior midline large volume trans-sphincteric tract. There is a secondary tract extending cranially abutting the levator plate that is in close proximity to the urethra. A standard written report along with a 3D model was presented to 17 colorectal surgeons via a web-form displaying the written report followed by the model. Each participant was asked to evaluate their understanding of i) anatomy and ii) clinical decision-making on a score of 1 (low) to 9 (high).

**Results:** Average confidence of 3D model informing understanding of fistula anatomy increased from 6.4 to 7.9, +1.5 (P <0.01). Average confidence around 3D model informing treatment management increased from 6.4 to 7.8, +1.3 (P <0.01).

**Conclusion:** 3D model of fistula anatomy improved clinical understanding of anatomy and clinical decision-making. Interestingly, the high quality of written report was frequently commented on by the surgical reviewers.

09:00 - 10:30 Room J1

*Scientific Session SS 6*

### Hepatocellular carcinoma: Response prediction and assessment

### SS 6.1

### Clinical and radiologic predictors of response to atezolizumab–bevacizumab in advanced HCC

S. Choi, S. Chung, J. Choi, K. Kim, H.-D. Kim, C. Yoo, B.-Y. Ryoo, S.S. Lee, W.-M. Choi, S.H. Choi*; Seoul/KR*

**Purpose:** This study aimed to identify clinical and radiologic characteristics that could predict response to atezolizumab–bevacizumab combination therapy in patients with liver-dominant advanced HCC.
**Material and Methods:** This single-center retrospective study included 112 patients with liver-dominant advanced HCC who were treated with atezolizumab–bevacizumab. Predictive factors associated with progressive disease (PD) at the best response based on Response Evaluation Criteria in Solid Tumors, Version 1.1 were evaluated using logistic regression analysis. Progression-free survival (PFS) was estimated by the Kaplan–Meier method and compared with the log-rank test.
**Results:** Of 112 patients with a median PFS of 15 weeks, 41 (36.7%) had PD during treatment. Factors associated with PD included the presence of extrahepatic metastases (adjusted odds ratio [aOR], 5.79; 95% confidence interval [CI], 1.65–20.25; p=0.006), the infiltrative appearance of the tumor (aOR, 3.95; 95% CI, 1.34–11.68; p=0.01), and the absence of arterial phase hyperenhancement (APHE) (aOR, 4.73; 95% CI, 1.61–13.94; p=0.005). Patients with two or more of these factors had a PD of 67.5% and a median PFS of 8 weeks, indicating a significantly worse outcome compared to the patients with one or fewer of these factors.
**Conclusion:** In patients with liver-dominant advanced HCC treated with atezolizumab–bevacizumab treatment, the absence of APHE, infiltrative appearance of the tumor, and presence of extrahepatic metastases were associated with poor response and survival. Evaluation of early response may be necessary in patients with these factors.

### SS 6.2

### Prediction of major pathologic response in HCC following downstaging with locoregional and systemic combination therapy

C. Yang, Y. Chen, H. Jiang, B. Song*; Chengdu/CN*

**Purpose:** To investigate the correlation between radiologic and pathologic responses following locoregional and systemic combination therapy, evaluate their prognostic values, and establish a prediction system for pathologic response in patients with unresectable HCC.
**Material and Methods:** This retrospective study included 112 consecutive patients with unresectable HCC who received combination therapy followed by liver resection or transplantation. Radiologic response was assessed with Response Evaluation Criteria in Solid Tumors (RECIST) 1.1 and modified RECIST (mRECIST). Performance in predicting pathologic response was assessed with the area under the receiver operator characteristic curve (AUC).
**Results:** Among radiologic and pathologic response criteria, major pathologic response (MPR, ≤10% residual viable tumors) was the only independent predictor of post-resection recurrence-free survival (RFS) (adjusted hazard ratio 0.34, 95%CI 0.16–0.72, p=0.004). Besides, mRECIST showed stronger correlation with pathologic response than RECIST 1.1 (Spearman r: 0.76 vs. 0.42, p<0.001). A prediction system for MPR was developed that included a combination of mRECIST response (i.e., >70% decrease of viable target lesions) with either >90% decrease in alpha-fetoprotein (AFP) (for AFP-positive group) or >80% decrease in protein induced by vitamin K absence-II (PIVKA-II) (for AFP-negative group), which yielded a respective AUC of 0.905 and 0.887. Moreover, patients with dual-positive response showed improved median RFS (not reached) than those with dual-negative response (7.1 months for the AFP-positive group and 13.3 months for the AFP-negative groups).
**Conclusion:** mRECIST was more indicative of pathologic response after combination therapy than RECIST 1.1. Integration of mRECIST with AFP or PIVKA-II responses allowed for accurate prediction of MPR and could support decision-making on subsequent curative-intent treatment.

### SS 6.4

### Comparison of LI-RADS treatment response algorithm and imaging features in triple-arterial MR acquisition in patients with HCC after locoregional therapies

A. Blandino[1], F. Khorasanizadeh[2], G. Bini[1], F. Matteini[1], R. Cannella[1], G. Brancatelli[1]*; [1]Palermo/IT, [2]Tehran/IR*

**Purpose:** To compare the liver imaging reporting and data system (LI-RADS) treatment response algorithm and imaging features in MRI acquired with a triple-arterial phase protocol in patients with treated HCC.
**Material and Methods:** A retrospective study was conducted on 71 MRIs of the liver in patients with history of HCC who underwent local–regional treatment (resection, transarterial chemoembolization and ablation). MRI examinations were acquired on a 1.5T (56.3%) or a 3T (43.7%) scanner using an extracellular (8.5%) or a hepatobiliary (91.5%) contrast agent with a triple-arterial phase imaging protocol. Images were analyzed by two radiologists who attributed, for each arterial phase (AP), a LR-TR category, evaluated the imaging features (APHE, treatment-specific enhancement pattern, washout appearance), and presence of artifacts based on a 5-point scale. McNemar and Wilcoxon signed-rank test were used to compare differences in imaging features and artifact scores among the triple-AP, respectively.
**Results:** A total of 54 (38 males, mean age: 72.2 years) patients with 71 post-treatment MRIs were included. APHE was significantly more on the second AP compared to the third AP (14.1% vs 5.6%, p=0.031). The second AP showed more commonly treatment specific enhancement pattern (8.5%, p values 0.250–1.000) and LR-TR viable category (16.9%, p value 0.063–0.727), without statistically significant differences. No significant differences were observed regarding the presence of artifacts (median 1 [interquartile range (IQR) 1–2] vs 1 [IQR 1–2] vs 2 [IQR 1–2]; p value 0.721–0.897).
**Conclusion:** Although the three arterial phases acquired with a triple-arterial phase MRI protocol show similar frequency of LR-TR features, the second AP seemed to convey more information and it can detect more commonly APHE.

### SS 6.5

### MRI-based preoperative prediction of early recurrence or drop-out from resection after neoadjuvant therapy in resectable HCC beyond Milan criteria

T. Zheng[1], L. Sheng[1], Y. Wang[1], M. Ronot[2], B. Song[1], H. Jiang[1]*; [1]Chengdu/CN, [2]Clichy/FR*

**Purpose:** To develop an MRI-based model for predicting early recurrence (≤2 years) or drop-out from surgical resection (SR) after neoadjuvant therapy in patients with resectable HCC beyond Milan criteria.
**Material and Methods:** This single-center retrospective study (October 2011 to March 2023) included consecutive patients with resectable Barcelona clinic liver cancer (BCLC) A/B HCC beyond Milan criteria undergoing upfront SR or neoadjuvant therapy. All images were independently evaluated by three blinded radiologists. In patients receiving upfront SR, an MRI-based Early Recurrence Outside Milan (EROM) score to predict early recurrence-free survival was developed via Cox regression analyses and compared with the BCLC and China Liver Cancer (CNLC) staging systems. Among patients treated with neoadjuvant therapy, the rates of drop-out from SR were compared between EROM-predicted high- and low-risk groups.
**Results:** 281 patients (median 56 years; 238 men) were included, among whom 220 (78.3%) underwent upfront SR and 61 (21.7%) received neoadjuvant therapy. Serum α-fetoprotein>20 ng/mL (HR, 2.03; *P*=0.007), largest tumor size (HR, 1.10; *P*=0.016), <50% arterial phase hyperenhancement (HR, 1.74; *P*=0.023), and infiltrative appearance (HR, 2.20; *P*=0.032) formed the EROM score, with higher testing dataset C-index (0.69) than the BCLC (0.52, *P*<0.001) and CNLC (0.53, *P*<0.001) systems. Among patients treated with neoadjuvant therapy, the drop-out rate was higher in the EROM-predicted high-risk group than in the low-risk one (44.2% vs. 11.1%, *P*=0.014).
**Conclusion:** In patients with resectable BCLC A/B HCC beyond Milan criteria, EROM allowed preoperative prediction of early recurrence after SR and informed neoadjuvant therapy drop-out risks.

### SS 6.6

### MRI-based peritumoral features could complement microvascular invasion for better postoperative early recurrence risk stratification in HCC

X. Jia, Y. Wang, B. Song, H. Jiang; *Chengdu/CN*

**Purpose:** Microvascular invasion (MVI) is an established adverse prognostic factor in HCC, but its assessment is subject to sampling errors and might be complemented by imaging. Therefore, we aimed to evaluate the incremental prognostic values of MRI-based peritumoral features to pathologically assessed MVI.
**Material and Methods:** This single-center retrospective study (April 2011 to May 2023) included consecutive patients who underwent curative resection for solitary BCLC 0/A HCC and preoperative MRI. Three blinded radiologists independently evaluated the MRIs for five peritumoral features. Cox regression analyses were conducted to identify predictive factors of early (≤2 years) recurrence-free survival (RFS), and an MRI criterion (MRIC) was established.
**Results:** 645 patients (median 53 years; 549 men) were included, 239 (37.1%) with MVI. At multivariable Cox analysis, MVI (HR, 2.45; *P*<0.001), portal venous phase peritumoral hypoenhancement (HR, 2.05; *P*<0.001) and T1 peritumoral hypointensity (HR, 1.51; *P*=0.03) were associated with early RFS. Therefore, the MRIC was considered positive if ≥one of these peritumoral MRI features were present. Patients with MVI-positive but MRIC-negative status showed similar cumulative 2-year RFS rate to those with MVI-negative but MRIC-positive status (55.9% vs. 60.4%, *P*=0.709), thus were grouped together as “either positive”. According to MRIC and MVI, patients were stratified into low-risk (both negative, *n*=360; 2-year RFS, 80.5%), intermediate-risk (either positive, *n*=192; 2-year RFS, 57.0%; *P*<0.001 against low-risk) and high-risk (both positive, *n*=93; 2-year RFS, 34.6%; *P*<0.001 against intermediate-risk) groups.
**Conclusion:** MRI-based peritumoral features could complement pathologically assessed MVI for more effective early recurrence risk stratification in patients with solitary HCC.

### SS 6.7

### Contrast-enhanced CT texture signature for programmed cell death protein ligand-1expression prediction and prognosis analysis in HCC patients receiving sorafenib treatment after surgery

F. Che, B. Song; *Chengdu/CN*

**Purpose:** Overexpression of programmed cell death protein ligand-1 (PD-L1) could cause tumor resistance to sorafenib in HCC patients. Our study aimed to predict the expression of PD-L1 and prognostic outcomes of HCC patients who received sorafenib treatment after surgery using contrast-enhanced CT (CECT) texture signature.
**Material and Methods:** This retrospective study included 88 HCC patients who received sorafenib treatment after surgical resection. Immunohistochemistry was used to assess the expression of PD-L1. Texture features were extracted based on CECT. The related significant features of PD-L1 expression were determined by the logistic regression analysis. The diagnostic performance of texture features was assessed by the area under the curve (AUC). Cox proportional-hazards model and Kaplan–Meier analysis were employed to select predictive features associated with recurrence-free survival (RFS) and overall survival (OS).
**Results:** The PD-L1+ HCC patients were found in 42 patients. Four wavelet features were proved associated with the PD-L1 expression. The wavelet_glszm_wavelet-LHL-ZoneEntropy feature achieved the best discriminative performance of the AUC as 0.708 [95% confidence interval (CI)= 0.595–0.821]. PD-L1+ was significantly related to worse RFS and OS (both P<0.05). The wavelet_glszm_wavelet-HLH-LowGrayLevelZoneEmphasis feature was associated with the risk of HCC recurrence (hazard ratio [HR], 0.43; 95% CI, 0.27–0.68; P><0.001) and overall patient mortality (HR, 0.34; 95% CI, 0.15–0.79; P=0.012).
**Conclusion:** The CECT texture signature may be an efficient technique for discriminating PD-L1 expression and predicting the survival of HCC patients receiving sorafenib treatment after surgery.

### SS 6.9

### Transplants for HCC: Do radiologists and pathologists agree?

J. Vassallo, D. Wu, C. Worsley, C. Bellamy, A. Adair, J. Gordon Smith; *Edinburgh/UK*

**Purpose:** Imaging plays a crucial role in selection criteria for liver transplantation in early-stage HCC associated with cirrhosis. We sought to establish the accuracy of pre-transplant imaging by comparing imaging reports to explant histopathology following liver transplantation for HCC as well as determine the impact on survival.
**Material and Methods:** All liver explant histopathology reports containing HCCs from January 2015 to December 2020 were cross-referenced with pre-transplant imaging reports (multiphase CT and MR liver with Primovist). The number and size of HCCs were extracted and compared and other lesions not identified or mischaracterised were also noted. Two-year survival outcomes were analysed using Kaplan–Meier estimates and the log-rank test.
**Results:** Out of 140 patients, 98 (70.7%) were within criteria, 35 (25%) were outwith criteria, 1 (0.7%) had an unclassifiable lesion and in 5 (3.6%) no malignancy was identified. 19 (14%) livers where found to have >5 HCCs. Of the 203 HCCs identified on explant pathology, 122 (60%) were definitively identified, 28 (14%) were indeterminate and 53 (26%) were not identified. The per-lesion sensitivity of pre-transplant imaging for HCC was 74% (including indeterminate lesions) and 18.5% for lesions <10mm. 2-year survival was 88.8%. Patients outwith transplant criteria with >5 HCCs (n=18, 13.4%) had the poorest survival at 77.8% (p= 0.009).
**Conclusion:** A significant number of transplanted patients are outwith transplant criteria. The per-lesion sensitivity for HCC was very poor for lesions <10mm. This resulted in 19 explanted livers having multiple small HCCs with these patients having worse outcomes. There is a need for accurately identifying this cohort of patients to optimise outcomes in liver transplantation.

### SS 6.10

### Factors predicting post-transplant HCC recurrence

F. Salahshour, M. Nassiri-Toosi, M. Taher, A. Jafarian, N. Ayoobi yazdi, F. Jamil, M. Taherzadeh, M. Safaei, F. Azmoudeh-Ardalan, F. Ghavidel; *Tehran/IR*

**Purpose:** Liver transplantation is a definite treatment for both HCC and end-stage liver disease. Organ shortage and risk of post-transplant HCC recurrence necessitate adherence to some eligibility criteria. In this study, we aimed to evaluate factors related to post-transplant HCC recurrence.
**Material and Methods:** All patients who underwent liver transplantation with the diagnosis of HCC from 2012 to 2023 and had follow-up surveys for more than 3 months were included. Demographic, clinical, laboratory, imaging, and histopathologic variables including alpha-fetoprotein level, and several eligibility criteria were extracted to evaluate factors related to post-transplant HCC recurrence.
**Results:** Among 122 patients who underwent liver transplantation between 2012 and 2023 with HCC-cirrhosis diagnosis, 94 patients met the inclusion criteria. Nine (9.5%) patients strike tumor recurrence. The mean recurrence time was 22 months (6–52 months) and mostly in the form of distant metastasis. All recurrent patients succumbed to death with a mean time interval of 7.5 months (1–21 months). Among study variables, just 4 histopathologic variables including the sum of tumor diameters (OR: 1.02, p value: 0.011, CI: 1.00 to 1.043), the sum of viable component diameters (OR: 1.02, p value: 0.006, CI: 1.007 to 1.044), outstanding UCSF criteria in the histopathologic exam (OR: 0.179, p value: 0.034, CI: 0.036 to 0.877), and microvascular invasion (OR: 5.3, p value: 0.045, CI: 0.85–4.06) were statistically significant for post-transplant HCC recurrence. None of the other variables such as the number of tumors or the size of the largest mass, alpha-fetoprotein level, nor LI-RADS classification was statistically significant.
**Conclusion:** Histopathology variables are the most important ones related to post-transplant HCC recurrence.

**09:00 - 10:30** **Room J2**

*Scientific Session SS 7*
### Primary sclerosing cholangitis and cholangiocarcinoma

### SS 7.1
### Disease severity prognostication in patients with primary sclerosing cholangitis using gadoxetic acid-enhanced MRI: A validation of the ANALI scores and comparison with the potential functional stricture

A. Kristic[1], S. Poetter-Lang[1], A. Messner[1], N. Bastati-Huber[1], R. Ambros[1], J. Kittinger[1], S. Pochepnia[1], S. Venkatesh[2], N. Kartalis[3], A. Ba-Ssalamah[1]; *[1]Vienna/AT, [2]Rochester, MN/US, [3]Stockholm/SE*

**Purpose:** To validate ANALI scores with and without gadolinium ($ANALI_{Gd}$ and $ANALI_{NoGd}$) and to compare their prognostic ability with the recently proposed potential functional stricture (PFS), all derived from unenhanced and gadoxetic acid-enhanced MRI (GA-MRI) in primary sclerosing cholangitis (PSC) patients.
**Material and Methods:** Five readers scored intrahepatic bile duct change severity, hepatic dysmorphia, liver parenchymal heterogeneity, and portal hypertension on GA-MRI, including 3D-T2-MRCP to generate $ANALI_{Gd}$ and $ANALI_{NoGd}$. They also evaluated 20-minute hepatobiliary-phase (HBP) images for PFS, i.e., absent contrast excretion in first-order bile ducts [i.e., left hepatic duct (LHD)/right hepatic duct (RHD)/common hepatic duct (CHD)/common bile duct (CBD)] or none at all vs normal biliary excretion, i.e., no functional stricture (NFS). Inter- and intrareader agreements were assessed and Kaplan–Meier curves were generated for survival analysis. Cox regression analyses were performed to evaluate association between $ANALI_{NoGd}$, $ANALI_{Gd}$, PFS and clinical scores, labs and outcomes.
**Results:** For 123 patients, mean age 40.5 years, Fleiss' kappa agreement was almost perfect-($\varkappa$=0.81) for PFS, but only moderate-($\varkappa$=0.55) for binary $ANALI_{NoGd}$. For binary $ANALI_{Gd}$, the agreement was slightly better on HBP (substantial-$\varkappa$ =0.64) than arterial phase (AP) (moderate-$\varkappa$ =0.53). Univariate Cox regression showed that hazard ratio (HR) for decompensated cirrhosis, orthotopic liver transplantation (OLT) or death was 3.15 for PFS (p<0.001) vs 6.42, (p<0.001) for $ANALI_{NoGd}$ vs 3.66, (p<0.001) for $ANALI_{Gd}$HBP and 3.79, (p<0.001) for $ANALI_{Gd}$AP. The multivariate analysis identified the PFS, three ANALI scores, and Revised Mayo Risk Score as independent risk factors for outcomes (HR 3.12, p<0.001; 6.12, p<0.001; 3.56, p<0.001; 3.59, p<0.001; and 4.13, p<0.001, respectively).
**Conclusion:** $ANALI_{NoGd}$ and GA-MRI-derived ANALI scores and PFS can non-invasively predict outcomes in PSC patients.

### SS 7.2
### An audit of the adequacy of gallbladder surveillance in primary sclerosing cholangitis at our institution: Are we following the guidelines and what were our findings?

S. Buelu, S. Curran, D. Gleeson, B. Rea; *Sheffield/UK*

**Purpose:** All guidelines recommend that patients with primary sclerosing cholangitis (PSC) undergo annual US surveillance for gallbladder cancer. This study's purpose was to audit the surveillance of our PSC patients and to review the findings and histology of any cholecystectomies.
**Material and Methods:** 138 patients were identified from our PSC database. Their US surveillance rate between 2012 and 2022 was assessed against our standard that all patients should have an annual US of the gallbladder (100%). Cross-sectional imaging including MRCP was deemed unsuitable for gallbladder surveillance and was excluded. The presence of polyps and their size was noted, and any cholecystectomies and subsequent histology reports were recorded.
**Results:** Of the 138 patients 9.4% (n=13) had regular and complete annual surveillance (one US per calendar year if eligible). The percentage of the population scanned showed a positive trend over the 10-year timespan with an improvement from 27.6% in 2012 to 83.3% in 2022 (mean=66.1%, median=68.4% (27.6%–90.2%)). Overall, 41.3% of the eligible scans were performed over the 10-year time span. 7 patients (5%) with polyps were identified via US surveillance (age 27–74 years, 5 male, 2 female). 4 patients underwent cholecystectomy and all harboured adenocarcinoma (n=3) or high-grade dysplasia (n=1) (mean=17.3mm, median=14.5mm (10–30mm)). 3 polyps (mean=4.7mm, median=5mm (4–7mm)) have not been excised. 25 cholecystectomies were performed for benign pathology and no incidental polyps or dysplasia was found.
**Conclusion:** Gallbladder polyp surveillance in patients with PSC has improved but remains suboptimal. Over 10 years, 7 patients (5%) developed gallbladder polyps, and of the four removed, all showed malignancy.

### SS 7.3
### Radiomics features for risk stratification in primary sclerosing cholangitis: A proof-of-concept study

C. Maino[1], L. Cristoferi[1], P. Franco[2], E. De Bernardi[1], M. Carbone[1], D. Ippolito[2]; *[1]Milan/IT, [2]Monza/IT*

**Purpose:** To identify the radiomics features, semi-automatically extracted from MRI-MRCP images, useful to identify patients at higher risk of clinical outcome development.
**Material and Methods:** Fifty-eight primary sclerosing cholangitis (PSC) patients with an MRI-MRCP study acquired with a standardized protocol were prospectively enrolled from Jan-2020 to Dec-2021. Blood tests and liver stiffness measurement (LSM) were collected close to the MRI-MRCP. Patients were classified into high risk or low risk for disease progression using the Mayo risk score (MRS) and LSM. Radiomics features have been extracted using PyRadiomics in each of the five MRI-MRCP sequences analyzed.
**Results:** Among the 58 patients, 15 (25.0%) and 17 (30.0%) were considered at high-risk using MRS and LSM, respectively. 107 radiomics features have been extracted from each MRI-MRCP sequence analyzed. The selection process individuated two features associated with high MRS: neighborhood gray-tone difference matrix (NGTDM)-busyness in the apparent diffusion coefficient (ADC) and gray-level run-length matrix (GLRLM)-run entropy in T2-spectral presaturation with inversion recovery (T2spir) showing both a mean cross-validated area under the curve (AUC) of 80%. The multivariable model, including both features, showed an AUC of 87% (SD 11%). When considering LSM (>9.6Kpa) as a stratifier of disease severity, gray level co-occurrence matrix (GLCM)-cluster shade in T1-weighted hepatobiliary phase (T1W HBP phase), GLCM-maximal correlation coefficient in T1W arterial phase, gray-level difference method (GLDM)-large dependence low gray level emphasis in ADC, and GLRLM-run entropy in T2spir showed an AUC of 85%, 83%, 85%, and 92%, respectively. The most accurate multivariable model included three variables: GLDM-large dependence low gray level emphasis in ADC, GLRLM-run entropy in T2spir and GLCM-cluster shade in T1W HBP phase with a median AUC of 96%.
**Conclusion:** This proof-of-concept study demonstrates the predictive value of the radiomics features in PSC and their potential role in risk stratification.

### SS 7.4

### Validating the additional value of quantitative MRI metrics for prediction of long-term survival in primary sclerosing cholangitis

T. Middelburg[1], B. Mol[1], L. Young[2], C. Ferreira[2], T. Davis[2], K. Horsthuis[1], Y. de Boer[1], A. van der Meer[3], A. de Vries[3], R. Dwarkasing[3], J. Bogaards[1], A.J. Nederveen[1], J. Stoker[1], C. Ponsioen[1]; *[1]Amsterdam/NL, [2]Oxford/UK, [3]Rotterdam/NL*

**Purpose:** MRCP is used for diagnosis and monitoring of primary sclerosing cholangitis (PSC). Unfortunately, current qualitative assessments and the potential predictive value of MRCP for long-term survival are limited by inter-observer variability. Quantitative assessment could overcome this limitation. This study aimed to validate the additional prognostic value of quantitative MRCP metrics in PSC.
**Material and Methods:** Retrospective clinical and imaging data were collected from the Dutch EpiPSC2 registry. MRCP data were modelled and quantified by artificial-intelligence-driven software (MRCP+, Perspectum Ltd). Data were randomized at 50:50 ratio into a derivation and validation set. Cox regression analysis was used to develop a binary risk classifier and assess the predictive performance for transplant-free survival. Subsequently, the derived risk classifier was applied on the validation set.
**Results:** 207 patients were included. Median transplant-free survival from MRCP onwards was 6.3 (IQR 3.5–8.8) years and 65 patients experienced an event. Total number of candidate strictures, time from diagnosis to MRCP, proportion of ducts with 3–5mm diameter and center of inclusion were associated with transplant-free survival. The derived risk classifier showed good predictive performance (C-statistic = 0.74) for identifying high-risk patients (HR = 5.4, 95% CI 2.3–12.4, p < 0.001) in the derivation set and remained consistent in 5-fold cross-validation (C-statistic = 0.71). The derived classifier showed comparable predictive performance (C-statistic = 0.69) for risk assessment in the validation set (HR = 3.5, 95% CI 1.7–6.9, p < 0.001).
**Conclusion:** This study confirms the additional predictive performance of MRCP+ metrics on long-term transplant-free survival in PSC patients.

### SS 7.5

### Cholangiocarcinoma in primary sclerosing cholangitis and the importance of MRI: Is our imaging good enough?

B. Rea, C. Wright, M. Sukhanenko, D. Gleeson; *Sheffield/UK*

**Purpose:** MRCP is considered the imaging standard for diagnosis and follow-up of patients with primary sclerosing cholangitis (PSC). Guidance from the International PSC study group (IPSCSG) highlighted the importance of MRCP technique and outlined a system for grading their quality. In 2017, a “minimum” or “more complete” MR protocol was outlined, and in 2021, a preference for using contrast-enhanced MRI was described. This study’s purpose was to review our PSC patients who developed cholangiocarcinoma and assess the quality of their imaging.
**Material and Methods:** Patients with PSC who developed cholangiocarcinoma from 2014 were identified. Their MRCP protocol and imaging quality for 3 years up to diagnosis were reviewed. Protocols selected were first compared with the 2017 recommendations and classified as “below minimum standard”, “minimum standard”, or “more complete standard”; and then with the 2021 reporting standards and classified as “preferred protocol” or not. MRCP quality was graded as per IPSCSG reporting standards, with studies graded as good or excellent grouped as “acceptable”.
**Results:** 13 patients with PSC developed cholangiocarcinoma within our 9-year timeframe and 36 MR studies met our inclusion criteria. 69.4% of the studies met the 2017 minimum standard protocol, with 22.2% below minimum standard; however, 91.7% did not meet the 2021 preferred standard protocol. Only 39.4% of the MRCPs performed 3 years prior to diagnosis were of “acceptable” quality, with 53.8% of the studies being “acceptable” 1 year prior.
**Conclusion:** MRCP/MRI is central to the diagnosis of cholangiocarcinoma in PSC. However, we frequently found a “minimum protocol” was employed, and our imaging quality was suboptimal, potentially impairing our ability to make an early diagnosis.

### SS 7.6

### Quantitative and qualitative imaging features to differentiate between intrahepatic cholangiocarcinoma and the benign mimicker sclerosing hepatic hemangioma

F. Willemssen, R. Boxhoorn, D. Bos, R. Dwarkasing; *Rotterdam/NL*

**Purpose:** Sclerosing hepatic hemangiomas (HHs) are hepatic hemangiomas with atypical imaging features which can mimic malignant liver lesions on MRI, especially intrahepatic cholangiocarcinoma (CCA). The purpose of this study was to differentiate these two entities based on MRI findings, using quantitative and qualitative imaging features.
**Material and Methods:** Between 2010 and 2022, all patients with a solitary liver lesion on MRI in which malignancy could not be excluded were selected. Patients with histopathological proven sclerosing HH and CCA were included. Qualitative imaging analysis of 4 important features was performed by 2 readers in consensus: perilesional arterial enhancement, bile duct dilatation posterior of the lesion, capsular retraction and targetoid appearance. Quantitative analysis of mean apparent diffusion coefficient (ADC) values was also performed.
**Results:** Sixteen patients (10♀) with HH and 53 patients (39♀) with intrahepatic CCA were selected. Perilesional enhancement was present in 5/16 HH and 10/53 CCA (p=0,3136), bile duct dilatation in 0/16 HH and 31/53 CCA (p=0,0001), capsular retraction in 1/16 HH and 23/53 CCA (p=0,0065) and targetoid appearance in 5/16 HH and 42/53 CCA (p=0,0006). Mean ADC values were, respectively, 2,16 x $10^{-3}$ $mm^2$ in HH and 1,37 x $10^{-3}$ $mm^2$ in CCA.
**Conclusion:** Sclerosing HH can mimic malignant liver lesions, especially intrahepatic CCA. Using both quantitative and qualitative imaging features on MRI, these lesions can be adequately differentiated.

### SS 7.7

### Diffusion-weighted fusion MRI in the study of cholangiocarcinoma: Preliminary results

E. Bardhi, F. Spoto, M. Todesco, B. Mascarin, F. Baratta, R. De Robertis, M. D’Onofrio; *Verona/IT*

**Purpose:** The purpose of this study is to evaluate how the identification of the lesion, its extension and vascular involvement can be improved using advanced imaging techniques, through the fusion of diffusion-weighted imaging (DWI) sequences with both T2-weighted sequences and post-contrast T1-weighted sequences acquired in the arterial and venous phases.
**Material and Methods:** A dataset of 48 images, obtained with 1.5 T MRI equipment, was analyzed by two radiologists with different experience. The major axis and vascular involvement were evaluated. For the quantitative variables, the statistical analysis was performed using the pairwise T-test, while for the qualitative variables, the intra-observer and inter-observer agreement was calculated using the Cohen’s Kappa test.
**Results:** From the quantitative analysis, significant differences between the readers were observed in T2-dependent sequences (p=0.04), but not in the fusion images (p=0.4), probably related to a better identification of the lesion in the fusion images and their different experience in abdominal imaging. In the qualitative analysis, intra-observer and inter-observer agreement was found to be minimal for venous vascular invasion for both the readers (K=0.25 and K=0.34), and consequently, image fusion could lead to a better identification of venous infiltration.
**Conclusion:** MR fusion imaging facilitates the evaluation of the extension of perihilar cholangiocarcinoma and vascular infiltration allowing a more precise staging and a better pre-surgical assessment improving therapeutic options.

### *SS 7.8*

### Imaging findings of perineural invasion in biliary adenocarcinoma

M.G. Lubner, S. Polanski, S.M. Daggett, R. Sappenfield, S.J. Lubner, L. Mao, P.J. Pickhardt*; Madison, WI/US*

**Purpose:** To evaluate cross-sectional imaging findings of pathology-proven perineural invasion (PNI) in patients with biliary adenocarcinoma.
**Material and Methods:** A retrospective review of biopsy and pathology databases from 2002 to 2022 yielded 81 patients (47 M/34 F, mean age 63±12 yrs) who had surgically resected adenocarcinoma of biliary origin (78 cholangiocarcinoma). Of these, 81 had the presence or absence of PNI described in the pathology report and imaging prior to surgery (median 26 days). Surgical and pathologic data were collected. Using CT/MRI nearest to surgery, imaging features suggestive of perineural invasion including peritumoral stranding, soft tissue stranding in the porta hepatis, enhancement greater than 2/3 of tumor volume on delayed hepatobiliary phase imaging, perivascular stranding/soft tissue and the presence of lymphadenopathy were assessed by two blinded readers. Univariate and receiver operating characteristic (ROC) analyses were performed.
**Results:** 25/81 patients (31%) had PNI noted at the time of surgical resection. Soft tissue stranding in the porta hepatis [odds ratio (OR) 1.9, p=0.07, area under the curve (AUC) 0.69] and higher tumor grade showed a positive association with PNI. Delayed enhancement of more than 2/3 of the tumor [hepato-pancreato-biliary (HPB) phase], suggesting a more fibrous tumor, was also associated with PNI, OR 2.4, p=0.2, AUC 0.72. Peritumoral and perivascular soft tissue stranding showed some association with PNI (OR 1.8, 1.5) although not statistically significant (p=0.1, 0.2). Mortality was not significantly different between the groups with or without PNI.
**Conclusion:** Peritumoral stranding, soft tissue stranding in the porta hepatis, and higher tumor grade are more likely to be associated with PNI in biliary adenocarcinoma.

### *SS 7.9*

### Preoperative prediction of perineural invasion in intrahepatic cholangiocarcinoma based on gadoxetic acid-enhanced MRI and MRI-derived habitats

X. Zhou, S.-T. Feng*; Guangzhou/CN*

**Purpose:** Habitat imaging allows for the quantification and visualization of various subregions within the tumor. We aim to develop an approach using gadoxetic acid (Gd-EOB-DTPA)-enhanced MRI and MRI-derived habitat imaging for preoperatively predicting the perineural invasion (PNI) of intrahepatic cholangiocarcinoma (ICC).
**Material and Methods:** Ninety-four patients were prospectively included at two institutions (training set, n=58; validation set, n=26; external test set, n=10, respectively). Preoperative clinical and MRI features were qualitatively and quantitatively reviewed. Tumor segmentation was based on pre- and post-enhanced T1 mapping images. Habitat characteristics were extracted for each tumor and 5 habitat regions were divided based on k-means clustering. All features were subjected to univariate analysis and comprehensively screened to establish a nomogram model. The diagnostic accuracy was evaluated using the area under the receiver operating characteristic curves (AUCs).
**Results:** PNI-positive ICC exhibited significantly higher serum tumor marker level, lower international normalized ratio (INR), more intrahepatic bile duct dilatation, vascular invasion, peritumoral arterial enhancement, and less likely to present with hepatic steatosis ($p < 0.05$). Habitat 1 was found a risk factor for PNI and was included in the nomogram model combined with CA199, INR, liver fat and intrahepatic bile duct dilation. The model demonstrated good predictive performance in the training, validation, and testing sets, with AUCs of 0.875, 0.842, and 0.750, respectively. The model could be used to stratify relapse-free survival (hazard ratio, 2.241; 95% confidence interval, 1.077–4.665; P=0.0309).
**Conclusion:** MRI and habitat imaging shows clinical potential for noninvasively and preoperatively determining PNI of ICC with high accuracy.

### *SS 7.10*

### Intra-arterial prostate-specific membrane antigen injection using hepatic arterial infusion pump in intrahepatic cholangiocarcinoma, a proof-of-concept study

M.M. Veenstra, E. Vegt, M. Segbers, S. Franssen, B. Groot Koerkamp, F. Verburg, M. Thomeer*; Rotterdam/NL*

**Purpose:** Prostate-specific membrane antigen (PSMA)-targeted tracers show increased uptake in several malignancies, indicating a potential for peptide radioligand therapy. Intra-arterial injection of radiotracers could increase the therapeutic window. This study aimed to evaluate the feasibility of intra-arterial injection of $^{68}$Ga-PSMA-11 for intrahepatic cholangiocarcinoma (ICC) and compare tracer uptake after intrahepatic arterial injection and intravenous injection.
**Material and Methods:** Patients with pathologically proven inoperable ICC received $^{68}$Ga-PSMA-11 (1,5 MBq/kg body weight) through a hepatic arterial infusion pump, followed by positron emission tomography (PET)/CT. Some days later, patients underwent PET/CT after intravenous $^{68}$Ga-PSMA-11 injection using the same protocols.
**Results:** Three patients were included. All tumors showed higher uptake on the intra-arterial scan compared with the intravenous scan (intra-arterial/intravenous standardized uptake value normalized by lean body mass (SUL) ratios 1,40–1,54). Uptake in normal liver tissues was similar between intra-arterial and intravenous scans (SUL ratios 0,86–1,13). In addition, $^{68}$Ga-PSMA-11 PET/CT showed diffusely increased uptake in large parts of the liver in one patient that seemed more extensive compared with the recent contrast-enhanced CT. CT three months post-PET/CT showed tumor progression in these exact segments.
**Conclusion:** Local intra-arterial PSMA injection is feasible in patients with ICC. The increased therapeutic window of intra-arterial injection compared with intravenous injection could be an interesting incentive to explore the possibility of PSMA-targeted peptide radioligand therapy for a subset of ICC patients.

11:00 - 12:30 Room J1

*Scientific Session SS 8*

### Advances in HPB imaging

### SS 8.1

### Development and external evaluation of a self-learning auto-segmentation model for colorectal cancer liver metastases assessment (coala)

J. Bereska[1], M. Zeeuw[1], L. Wagenaar[1], H. Jenssen[2], N. Wesdorp[1], D. van der Meulen[1], L. Bereska[1], E. Gavves[1], B. Janssen[1], M. Besselink[1], H. Marquering[1], J.-H. van Waesberghe[1], D. Aghayan[1], E. Pelanis[1], J. van den Bergh[1], I. Nota[1], S. Moos[1], G. Kemmerich[2], T. Syversveen[2], K. Kolrud[2], J. Huiskens[1], R.-J. Swijnenburg[1], C. Punt[1], J. Stoker[1], B. Edwin[2], Å. Fretland[2], G. Kazemier[1], I. Verpalen[1]; *[1]Amsterdam/NL, [2]Oslo/NO*

**Purpose:** Total tumor volume (TTV) is associated with overall and recurrence-free survival in patients with colorectal cancer liver metastases (CRLM). However, the labor-intensive nature of such manual assessments has hampered the clinical adoption of TTV as an imaging biomarker. This study aimed to develop and externally evaluate a CRLM auto-segmentation model on contrast-enhanced portal venous phase CT scans to facilitate the clinical adoption of TTV.
**Material and Methods:** We developed an auto-segmentation model to segment CRLM using 760 portal venous phase CTs (CT-PVP) of 363 patients. We used a self-learning setup whereby we first trained a teacher model on 99 manually segmented CRLM by three radiologists. The teacher model was then used to segment CRLM in the remaining 661 CT-PVPs for training the student model. We used the DICE score and the intraclass correlation coefficient (ICC) to compare the student model's segmentations and the TTV obtained from these segmentations to those obtained from the merged segmentations from three radiologists. We evaluated the student model in an external test set of 50 CT-PVPs from 35 patients with 72 CRLM from the Oslo University Hospital.
**Results:** The model reached a DICE score of 0.83 compared to an inter-rater DICE of 0.85. The ICC between the segmented volumes from the student model and from the merged segmentations was 0.97.
**Conclusion:** The developed colorectal cancer liver metastases auto-segmentation model achieved a high DICE score and near-perfect agreement (ICC) for assessing TTV.

### SS 8.2

### Should the term portovenous phase still be used when scanning the liver with Primovist?

S. Mokhles, K. Pieterman, M.M. Veenstra, M. Kuijpers, Z. Van Os, H. Muharam, T. Terkivatan, M. Doukas, D. Bos, M. Thomeer; *Rotterdam/NL*

**Purpose:** The aim is to compare head-to-head washout (WO) after administration of gadoxetate disodium and nonspecific contrast, and to investigate whether liver porta intensity difference (LPID) can be used to determine until when WO can still be assessed.
**Material and Methods:** The database included proven liver tumours from 2010 to 2021 and liver tumors without proof from 2019 to 2021. All MRIs with gadoxetate disodium were scanned according to a specific contrast protocol (bolus tracking) and predefined timepoints (PV1, PV2, TR1, and TR2), and were additionally scanned with nonspecific contrast (reference standard) (PV1= start arterial phase (SAP)+45 seconds; PV2= SAP+120 seconds; TR1= SAP+180 seconds and TR2= SAP+240 seconds).
**Results:** The study included 43 lesions (median 5.5 cm, min: 2 cm, max: 12 cm) with pathological confirmation and 51 without (median 4.9 cm, min: 1, max 12 cm). WO during PV1 had a sensitivity and specificity of 36% and 91%. In PV2, this was 27% and 75%. For TR1, this was 27% and 69%. For TR2, this was 36% and 67%. WO at scantimes before LPID=0 had a sensitivity and specificity of 57% and 67%. For WO at scantimes with LPID>100: 33% and 83%. For WO at scantimes with LPID>200: 25% and 87%.
**Conclusion:** Only at earliest timepoint PV1 or scantimes with LPID>100 or >200, pseudo-WO (false positives) was minimally present. Missed-WOs (false negatives) were extensively present at all scantimes. We suggest to use the term 'transitional phase' for all portovenous and late phases after gadoxetate disodium contrast injection.

### SS 8.3

### Efficacy of gadopiclenol in contrast-enhanced MRI of the liver: A post-hoc analysis

V. Vilgrain[1], C. Aubé[2], A. Paisant[2]; *[1]Clichy/FR, [2]Angers/FR*

**Purpose:** To evaluate the efficacy of gadopiclenol (Elucirem™, Guerbet), a high relaxivity macrocyclic gadolinium-based contrast agent (GBCA), at the dose of 0.05 mmol/kg in contrast-enhanced MRI of the liver.
**Material and Methods:** This is a post-hoc analysis of data from the subgroup of patients with liver lesions (N= 66) included in the prospective multicenter imaging study for evaluation of chest pain (PROMISE) phase III study, where 277 patients underwent two separate MRI examinations of different body regions, one with gadopiclenol (0.05 mmol/kg) and one with gadobutrol (0.1 mmol/kg). Lesion visualization parameters (border delineation, internal morphology, and contrast enhancement) were assessed by 3 independent blinded readers, through a qualitative scoring (1 to 4) of up to 3 most representative lesions in each patient. Percentage of enhancement (E%) and lesion to background ratio (LBR) were measured. An overall diagnostic preference was assessed in a global matched-pairs fashion by 3 additional blinded readers.
**Results:** For all readers, and all lesion visualization parameters, the difference in mean of scores showed the non-inferiority of gadopiclenol to gadobutrol (lower limit of 95%CI between −0.23 and −0.13, above the non-inferiority margin [−0.35]). There were no significant differences in LBR between the two GBCAs for all 3 readers (p≥0.1). E% was higher with gadopiclenol for 1 reader (p <0.0001) and not significantly different for the 2 others. The readers reported no preference between the images with gadopiclenol and those with gadobutrol in the majority of cases (69.7% to 87.9% of the evaluations).
**Conclusion:** Gadopiclenol at 0.05 mmol/kg is non-inferior to gadobutrol at 0.1 mmol/kg for contrast-enhanced MRI of the liver.

### SS 8.4

### Repeatability of MR-based muscle measurements - interim results from a US liver transplant waitlist natural history study

M.F. Forsgren[1], S. Lee[2], D. Kirkman[2], V. Patel[2], G. Roche[2], H. Kamal[2], O. Dahlqvist Leinhard[1], M.S. Siddiqui[2]; *[1]Linköping/SE, [2]Richmond, VA/US*

**Purpose:** Sarcopenia links to outcomes in liver transplantation and is typically assessed by imaging-based muscle measurements, e.g. L3 skeletal muscle index (L3-SMI). An MRI-based assessment (muscle assessment score [MAsS]) combining thigh muscle fat infiltration (MFI) and volume z-score (MVZ) has been developed to describe muscle health. The aim was to assess the repeatability of thigh and L3 muscle measurements in liver transplant candidates with liver cirrhosis.
**Material and Methods:** Fat-free muscle volume (FFMV), L3 skeletal muscle area (L3-SMA), MFI, L3-MFI, and MAsS were measured (AMRA® Researcher) based on an 8-min whole-body (WB)-MRI. Patients stood up and were re-positioned in the scanner in-between the repeated MRIs. Within-subject standard deviation (sw), repeatability coefficient (rc), and coefficient of variation (cv) were calculated.
**Results:** The first 35 patients (26 males; 29.4±6.9 kg/m$^2$; 55±11 y.o.; mainly MASH or alcohol-related liver cirrhosis) were included. The repeatability (sw, rc, and cv) was 0.06 L, 0.18 L, and 0.72% for FFMV; 3.45 cm$^2$, 9.55 cm$^2$, and 2.64% for L3-SMA; 0.21 pp, 0.57 pp, and 2.81% for MFI; and 0.54 pp, 1.49 pp, and 3.84% for L3-MFI. The repeatability (sw and rc) for MVZ was 0.07 SDs and 0.19 SDs.
**Conclusion:** Thigh volume repeatability was superior to the L3 skeletal muscle areas, and L3-SMA had 3.7x higher cv. Thigh MFI repeatability was better than for L3-MFI, meaning that smaller muscle changes may be detected in the thighs than in the L3 skeletal muscles. MVZ is distributed around 0; therefore, cv is not suitable. Thigh repeatability is high for patients with liver cirrhosis and in agreement with the literature for healthy volunteers.

### SS 8.5

### Comparison between conventional abdominal diffusion-weighted imaging and diffusion-weighted imaging based on deep learning reconstruction: Qualitative and quantitative evaluation

O. Ménez, M. Bali, N. Coquelet, J. Absil, V. Denolin, T. Benkert, E. Weiland, T. Metens; *Brussels/BE*

**Purpose:** This retrospective study aims to evaluate the non-inferiority of accelerated diffusion-weighted imaging (DWI) reconstructed with an experimental deep learning algorithm (DLR-DWI) compared to our standard upper abdomen DWI (S-DWI), in terms of image quality, lesion detection and characterization.
**Material and Methods:** Patients with at least one hepatic or pancreatic lesion were included (waived consent IRB-approved). DWI sequences were acquired in respiratory triggering at 3T (Siemens Vida) with identical parameters (b: 150/800 s/mm$^2$ acquisition pixel size 2.3x2.3x4mm, Grappa3), except for the number of repetitions leading to a 36% duration reduction and a super-resolution reconstruction algorithm for DLR-DWI. Liver and pancreas sharpness, the presence of noise and artefacts were qualitatively scored (five-grade scale); mean apparent diffusion coefficient (ADC) and standard deviation (SD-ADC) were measured in left and right liver and in the pancreas. Size and ADC were measured in the lesions. Comparisons were assessed with non-parametric tests.
**Results:** 55 patients with 81 lesions were included (26 F, 29 M; age: 64±14 years). On all images and ADC maps, DLR-DWI qualitative scores were significantly superior to DWI scores, besides the artefact score on b-800 images that was not different. In both liver lobes, DLR-DWI ADC values were significantly higher with lower standard deviations (all P<0.008), while not different in the pancreas: left/right liver/pancreas DLR-ADC 1038±169/962±153/1198±193; S-ADC 992±196/932±186/1168±216 $10^{-6}$ mm$^2$/s. Lesion size, mean ADC and SD-ADC did not differ between the sequences.
**Conclusion:** Compared with S-DWI, DLR-DWI is faster and qualitatively superior. Due to lower noise, ADC values are more elevated in the normal liver parenchyma, while in restricted diffusion lesions, the sequences are equivalent.

### SS 8.6

### Quantitative T2 mapping for the assessment of acute pancreatitis: A prospective study

F. Poroes[1], N. Vietti Violi[2], E. Uldry[2], T. Hilbert[2], F. Schütz[2], S. Schmidt Kobbe[2]; [1]*Fribourg/CH,* [2]*Lausanne/CH*

**Purpose:** To evaluate MR-derived pancreatic T2 mapping values in acute pancreatitis (AP) and correlate them with parameters of disease severity.
**Material and Methods:** In a single-center study, we prospectively included 76 consecutive patients with suspected AP between December 2020 and November 2022. The severity of AP was assessed clinically, biologically, and by contrast-enhanced CT (CECT) performed 48–72 h after symptom onset. MRI was also performed at ≤24h after CT. Two radiologists blinded to any clinical information independently evaluated the T2 values by placing three regions of interest inside the pancreatic head, body, and tail on the T2 mapping MR sequence. Results were compared with corresponding CECT images as the gold standard: patient parameters, such as age, gender, and body mass index (BMI); and clinical severity parameters. Inter-reader reliability was determined by calculating the interclass coefficient (ICC).
**Results:** Seventy-six patients (mean age, 52 years ± 18, 39 women) were evaluated. T2 values significantly correlated with the length of hospital stay (P = 0.01), CT severity index (P < 0.001), ICU admission (P < 0.05) and presence of organ failure (P < 0.05). T2 values did not depend on age, gender, BMI, anatomical location (pancreatic head, body, and tail) or main pancreatic duct dilatation. Interreader agreement was good (ICC = 0.85, 95% confidence interval: 0.77–0.90).
**Conclusion:** T2 mapping is useful for assessing the severity of AP enabling differentiation between mild and moderate/severe cases, and, thus, predicting patients at risk of complications and long hospital stays. Compared with current gold standards, T2-mapping offers a non-invasive, non-ionizing, contrast-free alternative.

### SS 8.7

### Chronic pancreatitis MRI score: A new proposal for diagnosis and disease severity

T. Tirkes[1], D. Yadav[2], D.L. Conwell[3], K. Jennings[4], L. Li[4], E. Fogel[1]; [1]*Indianapolis, IN/US,* [2]*Pittburgh, PA/US,* [3]*Lexington, KY/US,* [4]*Houston, TX/US*

**Purpose:** Generate a new diagnostic scoring system for chronic pancreatitis (CP) using MRI parameters in the well-phenotyped prospective evaluation of chronic pancreatitis for epidemiologic and translational studies (PROCEED) population. We aimed to generate a semi-quantitative composite score that would fulfill the need for a more accurate and early diagnosis of CP.
**Material and Methods:** The magnetic resonance imaging as a non-invasive method for the assessment of pancreatic fibrosis (MINIMAP) study prospectively imaged and analyzed 46 control, 45 suspected, and 46 definite CP patients enrolled at seven clinical centers in the USA from February 2019 to May 2021. Suspected and definite CP diagnoses were established based on imaging findings, symptomatology, and clinical presentation. MRI was performed using a standard imaging protocol on 1.5T Siemens and GE scanners. Logistic regression analysis generated multiparametric CP-MRI score, which included fat fraction (FF), arterial-to-venous enhancement ratio (AVR), and pancreatic tail diameter (PTD). If secretin-enhanced MRCP was performed, pancreatic ductal elasticity (PDE) was added to generate the CP-SMRI score.
**Results:** All MRI and MRCP parameters were significantly different between the control and definite CP cohorts: FF ($p$<0.001), AVR ($p$<0.01), PTD ($p$<0.001) and loss of PDE ($p$<0.001). Using a multiparametric score yielded better diagnostic performance than the individual parameters. CP-MRI and CP-SMRI had cross-validated area under the curves (AUCs) of 0.84 and 0.86, respectively. The score of 2.6 was 87% sensitive, 68% specific using CP-SMRI and 89% sensitive, 67% specific using CP-MRI for the diagnosis of CP.
**Conclusion:** The multiparametric approach yields higher diagnostic performance to diagnose CP than individual parameters. We propose two comprehensive semi-quantitative criteria that combine three parenchymal MRI features and an optional dynamic secretin-enhanced MRCP feature. Larger population studies with multiple observers and longitudinal analyses are warranted.

### SS 8.8

### Quantitative analysis of the pancreas with 3D multi-echo Dixon MRI and US point shear wave elastography: Correlations with endocrine and exocrine functions

F. Spoto, R. De Robertis, A. Garofano, C. Licata, M. D'Onofrio; *Verona/IT*

**Purpose:** To evaluate the correlations between proton density fat fraction (PDFF), R2*, volume, and stiffness of the pancreatic parenchyma with endocrine and exocrine pancreatic functions.
**Material and Methods:** On the same day, 117 subjects underwent 3D multi-echo Dixon MRI and transabdominal US point shear wave elastography (pSWE). Whole-gland volumes of interest (VOIs) were segmented on MR images to extract parenchymal volumes and median PDFF and R2* values; nine pSWE measurements were performed, and median values were calculated. The results were compared between the patient's groups (preserved function vs. pancreatic insufficiency and preserved function vs. endocrine insufficiency vs. exocrine insufficiency vs. endocrine and exocrine insufficiency) using the Mann–Whitney test and the Kruskal–Wallis. P values <0.05 were statistically significant.
**Results:** Ninety-three patients were included (50 men and 43 women, mean age 61 years; age range, 18–85 years). 28 patients had pancreatic insufficiency (19 endocrine, 4 exocrine, and 5 endocrine and exocrine). PDFF was the only parameter with a significant difference between the groups, being significantly higher in patients with pancreatic insufficiency than in those with preserved pancreatic function (median 7% vs 3,1%, p=0,008).
**Conclusion:** Pancreatic fat content measured by MRI-PDFF is associated with pancreatic insufficiency.

### SS 8.9

### Reappraising imaging features of pancreatic acinar cystic transformation: A real-life single-centre study of 64 patients

L. Aguilera, C. Boros, F. Bonvalet, L. de Mestier, F. Maire, P. Levy, J. Cros, V. Rebours, M. Ronot; *Clichy/FR*

**Purpose:** Imaging features of pancreatic acinar cystic transformation (ACT) have been published. We aimed to describe the clinical and radiological characteristics of patients with a presumed pancreatic ACT diagnosis, reappraising the value of these imaging criteria.
**Material and Methods:** This is a single-centre retrospective study (2003–2021) of consecutive patients with a presumed diagnosis of ACT as suggested by the local expert multidisciplinary case review board. Patients without available imaging (CT or MRI) for review were excluded. Imaging was reviewed by two radiologists. Patients were classified into "certain" ACT (if ≥2 imaging criteria and no differential diagnosis) or "uncertain" ACT (if ≥1 imaging criteria and suggested differential diagnoses). Groups were compared with Chi-2 and Mann–Whitney U tests.
**Results:** 64 patients (35 males, [55%]) were included. ACT was considered "certain" for 34 patients (53%) and "uncertain" for 30 patients (47%). The number of ACT criteria did not differ between the groups, with 91.2% of the patients with ≥3 ACT imaging criteria in the "certain" group *vs.* 93.3% in the "uncertain" group (p=0.88). In the "uncertain" group, the principal suggested differentials were branch-duct (BD) IPMN (18/30 patients, 60%), calcifying chronic pancreatitis (CCP, 8/30 patients, 27%), both (three patients, 10%), and serous cystadenoma (one patient, 3%). Calcifications were significantly more frequent in the "uncertain" group (89% *versus* 63% in the "certain" group, p=0.02).
**Conclusion:** Published ACT imaging criteria are frequently associated with features suggesting differential diagnoses. They appear insufficient to reach a final diagnosis in a subset of patients.

### SS 8.10

### Correlation between visceral fat volume, sarcopenia and post-operative complications after major pancreatectomy

L. Fortuna, M. Bariani, P. Sbeghen, F. Omboni, I. Taama, G. Mansueto, G.A. Zamboni; *Verona/IT*

**Purpose:** Our purpose was to analyze the correlations between visceral obesity, sarcopenia and post-operative complications after major pancreatic resections.
**Material and Methods:** Informed consent for the utilization of clinical and radiologic data was provided by all patients (PAD-R registry, n1101CESC). We reviewed 255 patients who underwent major pancreatectomy at the Verona Pancreas Institute. We used a commercially available software (Syngo.via, Siemens) to segment visceral fat and paraspinal and psoas muscles at the umbilical level. For each, we logged volume ($cm^3$) and mean density values (HU). We evaluated the presence of post-operative pancreatic fistula (POPF) and post-operative hyperamylasemia (POH). Quantitative variables were described as mean and compared using Student's t-test. p values <0.05 were considered statistically significant.
**Results:** We enrolled 255 patients, 164 M and 91 F, mean age 64 years. Patients who developed a clinically significant POPF (n=106) had a higher visceral fat volume than patients without a clinically significant POPF (n=149) (p<0.0001). Patients with POPF had a higher muscle volume than patients without a significant POPF (p= 0,0106). No difference was observed in muscle density between POPF and non-POPF patients. No difference was observed in visceral fat or muscle volume between patients who developed post-operative hyperamylasemia or not.
**Conclusion:** In our series, visceral fat was correlated with the onset of post-operative pancreatic fistula, especially clinically significant fistula.

**11:00 - 12:30** **Room J2**

*Scientific Session SS 9*

### Rectal cancer response prediction and assessment

### SS 9.1

### Texture analysis and prediction of response to neoadjuvant treatment in patients with locally advanced rectal cancer

I. Mariani[1], C. Maino[2], P. Franco[1], T. Giandola[1], S. Drago[1], D. Ippolito[1]; [1]*Monza/IT,* [2]*Milan/IT*

**Purpose:** To retrospectively collect radiomic data from preoperative rectal MR and determine their possible relationship with response to neoadjuvant treatment.
**Material and Methods:** 88 patients with biopsy-proven advanced rectal adenocarcinoma and staging MR were enrolled. After neoadjuvant therapy and rectal anterior resection, tumour regression grade (TRG) was collected: TRG 1–2 were classified as responders, while TRG 3–5 as non-responders. Texture analysis was conducted by LIFex software on T2-weighted para-axial MR sequences, and a region-of-interest was manually drawn on a single slice. Features with a Spearman correlation index >0.5 have been discarded and a LASSO feature selection has been applied. Selected features were trained using *bootstrapping*.
**Results:** 49 patients (55.8%) were considered responders, while 39 (44.2) as non-responders. *GLCM_Homogeneity* and *Discretized Histo Entropy log 2* were associated with responders' classes. Regarding *GLCM_Homogeneity*, the area under the receiver operating characteristic curve (AUC), sensitivity (sens), specificity (spec), positive predictive value (PPV), and negative predictive value (NPV) were 0.779 (95% CIs = 0.771–0.816), 86% (80–90), 67% (60–71%), 81% (76–84), and 88% (84–90), respectively. Regarding *Discretized Histo Entropy log 2*, diagnostic values were as follows: AUC = 0.775 (0.700–0.801), sens = 80% (74–83), spec = 63% (58–69%), PPV = 77% (70–81), and NPV = 82% (80–85). By combing both radiomics features, diagnostic accuracy increased (AUC = 0.844, p<0.05). Finally, the AUC of 1000 bootstraps was 0.810.
**Conclusion:** Texture analysis can be considered an advanced tool to determine a possible correlation between pre-surgical MR data and response to neoadjuvant therapy. Further studies with a larger cohort of patients should aim to validate these preliminary data.

### SS 9.5

### The comparison of conventional diffusion-weighted imaging versus diffusion kurtosis imaging and amide proton transfer-weighted imaging in evaluating response to neoadjuvant therapy in rectal cancer: A prospective study

X. Gong[1], X. Zhang[2], B. Song[1]; [1]*Chengdu/CN,* [2]*Wuhan/CN*

**Purpose:** To assess the efficacy of diffusion kurtosis imaging (DKI) and amide proton transfer (APT)-weighted imaging in distinguishing pathological complete response (pCR) following neoadjuvant therapy in patients with rectal cancer (RC), compared to conventional diffusion-weighted imaging (DWI).
**Material and Methods:** A total of 83 patients with biopsy-proven RC treated with neoadjuvant therapy were prospectively recruited. All individuals underwent preoperative MR scans, including T2-weighted and the above three sequences. According to the pathology, participants were grouped into pCR and non-pCR groups. DKI parameters, including mean diffusivity (MD) and mean kurtosis, APT-weighted signal intensity, and mono-exponential apparent diffusion coefficient (ADC) values were calculated and analyzed between the groups.
**Results:** 76 patients were successfully included, including 15 with pCR and 61 with non-pCR. The pCR group exhibited significantly higher ADC and MD values than the non-pCR group (1.528 ±0.166 vs. 1.155 $\pm 0.177 \times 10^{-3}$ $mm^2/s$, p < 0.001 and 1.493 ±0.265 vs. 1.290 $\pm 0.201 \times 10^{-3}$ $mm^2/s$, p = 0.002, respectively). No significant differences were observed in mean kurtosis or APT signal intensity between the groups. The diagnostic accuracy was 85.5% (95% CI: 85.2, 85.8; 65 of 76) for the ADC value and 72.4% (95% CI: 71.9, 72.9; 55 of 76) for the MD value.
**Conclusion:** Preoperative ADC value may serve as a more valuable imaging biomarker for non-invasively predicting pCR in RC patients undergoing neoadjuvant therapy than DKI and APT-weighted imaging metrics.

### SS 9.6

### Accuracy of post-neoadjuvant therapy MRI of involvement of anal sphincter in rectal cancer: Comparison with pathology

M. El Homsi, L.J. Fuqua, T.H. Kim, M. Fernandes, L. Fernandes De Paula Tito Jorge, J. Shia, C. White, M. Capanu, I. Petkovska; *New York, NY/US*

**Purpose:** To assess the accuracy of post-neoadjuvant therapy (NAT) MRI for involvement of the anal sphincter in comparison with pathology in rectal cancer.
**Material and Methods:** This is a retrospective study including 49 patients with the involvement of anal sphincter by rectal cancer at baseline MRI undergoing NAT followed by post-NAT MRI. Two GI specialized radiologists reader 1 (R1) with 20 years and reader 2 (R2) with 1 year of experience reviewed both MRIs and assessed the involvement of anal sphincter by tumor at baseline and by tumor and scar after NAT. 32 (65%) patients had surgical resection after NAT, and pathology was reviewed by a GI specialized pathologist to assess involvement of anal sphincter. Rater agreement between the radiologists, and pathologist, was assessed using Cohen's kappa.
**Results:** There was moderate agreement between readers for involvement of anal sphincter at baseline (kappa 0.53) and fair agreement at post-NAT MRI (kappa 0.31). There was moderate agreement between R1 and pathologist (kappa 0.55) and between R2 and pathologist (kappa 0.53). When comparing agreement between R1, R2 and pathologist for type of involvement of anal sphincter, there was slight agreement for the presence of scar (kappa 0.12 and 0.09), fair agreement for the presence of tumor for R1 (kappa 0.25) and no agreement for R2 (kappa –0.32).
**Conclusion:** Both radiologists had good agreement for involvement of anal canal post-NAT in rectal tumor compared to the pathology with slightly better accuracy for tumor involvement by the most experienced reader which may reflect years of training.

### SS 9.7

### Creating machine-learning response-assessment models with robust ground-truth labels through innovative fusion of rectal MRI and whole-mount histopathologic specimens

N. Horvat[1], J.M. Santos[1], J. Heiselman[1], C. Firat[1], T.H. Kim[1], J. Chakraborty[1], A. Assuncao[2], J. Shia[1], J. Garcia-Aguilar[1], M.J. Gollub[1]; *[1]New York, NY/US, [2]Sao Paulo/BR*

**Purpose:** MRI-based radiomics holds promise as an objective tool for predicting treatment response in the context of rectal cancer. However, its current clinical utility remains limited by its generalizability. Specifically, radiomic models have yet to be trained using whole-mount histology (WMH) as the ground truth, even though WMH is considered the gold-standard reference method for point-by-point comparison. This study assessed a rigid point-based registration method to evaluate the WMH and MRI fusion accuracy.
**Material and Methods:** 18 consecutive patients with RC who underwent neoadjuvant therapy followed by total mesorectal excision from 2018 to 2021 were included. A multimodal radiology–pathology–image-registration workflow was developed. 1) Tumor bed, internal and external borders of the rectum, and eight corresponding landmarks were manually delineated on MR and WMH images by a radiologist and a pathologist, respectively. 2) Rigid point-based registration of images with automated rescaling was computed via the delineated landmarks. 3) Biomechanically constrained plane strain elastic deformable registration was computed from the initial rigid alignment between the annotated contours to account for MR and WMH rectal distension differences. 4) We performed image registration using a combination of in-house rigid registration, active contours, and finite-element software. 5) Outputs from the multimodal image fusion system were rendered in 3D Slicer.
**Results:** Dice overlap and modified Hausdorff distance of the delineated MR and pathology images showed a significantly good correlation between external and internal border segmentations (P values<.05, comparing the mean values of each case across the mean values from each of the three levels per case).
**Conclusion:** Deformable registration significantly improves the internal and external contour agreements over rigid point-based registration.

### SS 9.8

### Enhancing rectal cancer re-staging accuracy: Integration of radiomics score with radiologist subjective analysis

J.M. Santos, M. El Homsi, M.C. Fernandes De Paula Tito Jorge, N. Horvat; *New York, NY/US*

**Purpose:** External validation and evaluation of the efficacy of integrating a newly developed radiomics score (radscore) with MR tumor regression grade (mrTRG) in improving the accuracy and consistency of rectal cancer re-staging.
**Material and Methods:** Radscore was developed from pre- and post-treatment MRI scans of 180 patients, achieving accuracy comparable to mrTRG. The study then focused on the influence of radscore on radiologists' subjective evaluations during rectal cancer re-staging. We retrospectively analyzed 60 patients (20 with pCR and 40 without) in a two-phase approach: initially using only mrTRG, followed by a combined assessment with mrTRG and radscore. This involved two junior and two senior radiologists, examining intra/inter-observer variability and the accuracy of pCR prediction.
**Results:** The integration of radscore with mrTRG led to a significant improvement in the accuracy of pCR prediction, increasing from 75% with mrTRG alone to 88% when combined with radscore (p<.01, 95%CI [83%, 93%]); reduced both intra-/inter-observer variability, with intra-observer variability decreasing from 20% to 10% (p<.05, 95%CI [5%, 15%]), and inter-observer variability from 25% to 12% (p<.05, 95%CI [7%, 17%]). The combined method demonstrated superior performance in differentiating patients with pCR from those without, evidenced by an increase in sensitivity from 70% to 85% (95%CI [78%, 92%]) and specificity from 80% to 90% (95%CI [85%, 95%]), both with p-values<0.05.
**Conclusion:** Integrating radscore with mrTRG represented a significant advancement in the re-staging of rectal cancer. This approach not only improves the accuracy of pCR prediction but also ensures greater consistency among radiologists, suggesting that advanced imaging analytics like radscore can substantially enhance clinical decision-making in rectal cancer treatment.

### SS 9.9

### Rectal cancer magnetic resonance imaging proforma implementation: A Q-methodology study of relative importance of barriers to adoption

S. Alderson, C. Muthoo, H. Rossington, P. Quirke, D. Tolan; *Leeds/UK*

**Purpose:** MRI of rectal cancers (rcMRI) provides the most accurate staging for treatment decisions. Guidelines advocate the use of structured reporting templates to reduce the variability of reporting and optimise treatment decisions, with missing information potentially influencing disease-free survival. We aimed to understand the relative importance of different barriers to rcMRIs use by radiologists. Q-methodology combines qualitative and quantitative research methods to identify shared viewpoints about a particular subject and has potential value in predicting and responding to challenges in implementing interventions.
**Material and Methods:** Statements (the Q-set) on barriers and facilitators to proforma use were developed from a previous interview study. Radiologists across a regional bowel cancer improvement programme (population 5.7 million) were invited. Participants electronically sorted the statements in a forced normal distribution according to their beliefs (q-sort). Factor arrays were created from the data analysis to identify shared viewpoints.
**Results:** Twenty-six radiologists completed a q-sort representing nine hospital trusts. Three distinct viewpoints were identified, explaining 50% of variance. (1) Confident users: this group found rcMRI proformas helpful, easy to use, with no significant barriers. (2) Dislike proformas: this group prefers free-text narratives with barriers related to individual preferences and proforma characteristics. (3) Struggling champions: this group understood the evidence for rcMRI proformas but found organisational barriers that prevented regular use.
**Conclusion:** Q-methodology has defined the barriers and their relative importance for rcMRI proforma reporting. Targeted implementation strategies are required to increase the use and quality of rcMRI reports in a clinical practice.

### SS 9.10

**SPAR rectal MRI technique sub-study, with technique and reporting survey in Australia and New Zealand**

N. Chai[1], G. Malalagama[1], M. Jameson[2], S. Ackland[3], K. Gormly[1]; [1]Adelaide/AU, [2]Hamilton/NZ, [3]Lake Macquarie/AU

**Purpose:** To assess improvement in rectal MRI technique and reporting in Australia and New Zealand (ANZ) since PETACC-6 MRI audit of 2011, showing 36% high resolution (HR) T2 <1.3 mm$^3$.

**Material and Methods:** A subset of 53/135 patients from all 15 sites in the AGITG-sponsored SPAR trial had pre- and post-treatment scans analysed for MRI sequences and HR T2 parameters. A survey was sent to radiologists in ANZ assessing reporting templates, reporting elements, MRI sequences and HR T2 parameters.

**Results:** 100 scans were reviewed across 29 magnets from 3 vendors at 1.5 and 3T between 2018 and 2023. HR T2 voxel size <1.2mm$^3$ was obtained on 14/17 magnets (post-treatment trial MRIs) and 19/29 magnets (pre- and/or post-treatment MRIs). HR T2 voxel <1.2mm$^3$ across the three vendors was 15/19, 3/5 and 1/5. 100% of the scans included sagittal T2 and HR T2 axial oblique (ax obl) sequences. 92% diffusion (42% ax obl alignment), 19% fat sat T2, 4% T2 volume and 2% post-contrast. 44 survey responses were obtained across all the states of Australia and New Zealand. 20 reported themselves a subspecialist. Sequences performed: 100% sagittal T2, 98% HR T2 ax obl, 98% diffusion (52% ax obl alignment), 16% fat sat T2, 14% T2 volume, 2% post contrast. 22 provided adequate technical information, with HR T2 voxel <1.2mm$^3$ 77% and <1.3mm$^3$ 86%. 80% use a template. >90% include all recommended reporting elements, except distance from puborectalis. 82% use mrTRG to report post-treatment scans.

**Conclusion:** There has been a significant improvement in ANZ MRI technique and reporting since 2011.

**14:30 - 16:00** **Room J2**

*Scientific Session SS 10*

### Liver: steatosis, MASLD, fibrosis

### SS 10.1

**Ultrasound-based steatosis grading system using 2D-attenuation imaging: An individual patient data meta-analysis with external validation**

C. Hobeika[1], M. Ronot[1], D. Valla[1], J.-M. Correas[2], V. Vilgrain[1], M. Dioguardi Burgio[1]; [1]Clichy/FR, [2]Paris/FR

**Purpose:** This one-stage individual patient data (IPD) meta-analysis aimed to create an attenuation imaging (ATI)-based steatosis grading system.

**Material and Methods:** A systematic review (EMBASE+MEDLINE, 2018–2022) identified studies, including patients with histologically or MRI-proton density fat fraction (MRI-PDFF)-verified ATI values for grading steatosis (S0 to S3). One-stage IPD meta-analyses were conducted using generalised mixed models with a random study-specific intercept. Created ATI-based steatosis grading system (aS0 to aS3) was externally validated on a prospective cohort of patients with type 2 diabetes and non-alcoholic fatty liver disease (NAFLD) (n=174, histologically and MRI-PDFF-verified steatosis).

**Results:** Eleven enrolled studies included 1374 patients, classified into S0, S1, S2, and S3 in 45.4%, 35.0%, 9.3%, and 10.3% of the cases. ATI was correlated with histologically (r=0.60; 95%CI: 0.52, 0.67; p<0.001) and MRI-PDFF (r=0.70; 95%CI: 0.66, 0.73; p<0.001) quantified steatosis, while uncorrelated with liver stiffness (r=0.03; 95%CI: −0.04, 0.11, p=0.343). Steatosis grade (coefficient: 0.27; 95%CI: 0.07, 0.47; p=0.008) was the only independent factor associated with ATI, while age, sex, body mass index (BMI), chronic hepatitis, and alcohol consumption were not. ATI marginal means within S0, S1, S2, and S3 subpopulations were 0.59 (95%CI: 0.56, 0.61), 0.69 (95%CI: 0.65, 0.72), 0.77 (95%CI: 0.73, 0.81), and 0.84 (95%CI: 0.80, 0.89) dB/cm/MHz; all contrasts between grades were significant (p<0.0001). Three ATI thresholds were calibrated to create a new ATI-based steatosis grading system (aS0 to aS3, cutoffs: 0.66, 0.73, and 0.81dB/cm/MHz). Its external validation showed Obuchowski measures [to interpret as area under the curve (AUC) values] of 0.84±0.01 and 0.82±0.02 with histologically and MRI-PDFF-based references.

**Conclusion:** ATI is a reliable non-invasive marker of steatosis. This validated ATI-based steatosis grading system could be valuable in assessing NAFLD patients.

### SS 10.2

### Performance of ultrasound liver fat quantification for the assessment of hepatic steatosis using MRI fat fraction as reference standard

F. Matteini, R. Cannella, M. Mazzola, D. Gagliano, T. Bartolotta*; Palermo/IT*

**Purpose:** To evaluate the performance and the reproducibility of US-based liver fat quantification (LFQ) for the assessment of hepatic steatosis in patients with MRI fat fraction.
**Material and Methods:** This prospective study included adult patients who underwent quantification of hepatic steatosis with US and MRI. US examination was acquired with LFQ measurements, hepatorenal index (HRI), and 2D shear-wave elastography (SWE) based on the median values of 10 consecutive measurements by two operators. The reference standard was the fat fraction quantification obtained with mDixonQuant sequence on a 3T MR scanner. Area under the receiver operating characteristic (ROC) curves (AUCs) were calculated to determine the diagnostic performance with optimal cutoffs based on the Youden index. The intraclass correlation coefficient (ICC) was used to assess the inter- and intra-observer reproducibilities.
**Results:** Ninety-three patients (47 females, median age 65.0 [IQR 54.5, 73.0] years) were prospectively enrolled. On MRI, 28/93 (30.1%), 3/93 (3.2%) and 5/93 (5.4%) patients had grade 1, 2 and 3 steatosis, respectively. Median LFQ was 0.58 dB/cm/MHz (IQR: 0.01 dB/cm/MHz; IQR/median ratio: 2.0%). The AUCs for steatosis grade ≥1 were 0.898 (cutoff>0.60, sensitivity of 80.6%, specificity of 89.5%) for LFQ and 0.712 (cutoff>1.29, sensitivity of 67.7%, specificity of 76.4%; p=0.005) for HRI. The AUCs for steatosis grade ≥2 were 0.989 (cutoff>0.67, sensitivity of 100%, specificity of 95.3%) for LFQ and 0.848 (cutoff>1.55, sensitivity of 100%, specificity of 75.9%; p=0.005) for HRI. The inter- and intra-observer reproducibilities were good for LFQ with and ICC of 0.85 (95%CI: 0.77–0.89) and 0.80 (95%CI: 0.69–0.86), respectively.
**Conclusion:** US-based LFQ provides a good-to-excellent performance for the diagnosis of hepatic steatosis and high reproducibility.

### SS 10.3

### 3D quantitative analysis of liver steatosis using virtual volumetric mass fat fraction on non-contrast CT

H.-J. Chung, J.-M. Kim, S.J. Park*; Seoul/KR*

**Purpose:** The objective of this study is to evaluate the assessment of liver steatosis using virtual volumetric mass fat fraction (virtual-VMFF) on non-contrast CT images.
**Material and Methods:** The proposed virtual-VMFF is defined as the ratio of estimated fat mass to expected liver mass. The estimated fat mass is calculated using a density of 0.911 for liver voxels with attenuation below zero, and 0.955 for those between zero and mean splenic attenuation. The expected liver mass is derived from a density of 1.079 applied to the liver's volume. The dataset, comprising data from 145 adult patients, was utilized to compare the proposed method with existing methods, which are liver attenuation<40 HU and liver/spleen attenuation ratio<1.0. The study utilized commercial body composition analysis software for all analyses (DeepCatch v1.X, MEDICAL IP Co., Ltd.).
**Results:** The correlation between the proposed virtual-VMFF and the average liver attenuation and liver–spleen attenuation ratio in the first dataset was evaluated using the R-squared value, which showed strong correlations of 0.96 and 0.85, respectively. The proposed virtual-VMFF was 4.39% for an average liver attenuation value of 40 HU and 3.60% for a liver–spleen attenuation ratio of 1.0.
**Conclusion:** In this study, we proposed the virtual-VMFF as a method for quantifying the mass of abnormal areas in the liver. Our findings demonstrated that virtual-VMFF correlates well with existing methods, showcasing its potential as a promising tool in the quantification of liver fat using CT images.

### SS 10.4

### Advanced CT-derived imaging markers for predicting cardiometabolic health risks: Insights from seven years of cohort data analysis

S.J. Park[1], J.-M. Kim[1], H.-J. Chung[1], Y. Chang[1], R. Kwon[1], J. Kang[1], Y. Kim[1], J.H. Choi[1], H.-S. Jung[1], G.-Y. Lim[1], J. Ahn[1], S. Wild[2], C.D. Byrne[3], S. Yoon[1], S. Ryu[1]*; [1]Seoul/KR, [2]Edinburgh/UK, [3]Southampton/UK*

**Purpose:** This study evaluates the efficacy of automated CT-derived imaging markers in predicting various cardiometabolic health risks.
**Material and Methods:** In a cohort of 32,166 Korean adults (mean age 44.6 years) undergoing positron emission tomography (PET)–CT scans for health checkups, we employed DeepCatch (v1.x, Medical IP, South Korea) to analyze fully automated CT-based image markers. These included visceral/subcutaneous fat, muscle area, bone density, liver fat fraction, and aortic calcification. Body composition areas were normalized to height squared in meters. The assessment of predictive performance was conducted using the area under the receiver operating characteristic curves (AUROCs) and Harrell's C-index for survival analysis, focusing on various cardiometabolic health risks.
**Results:** Our findings showed that liver fat fraction's AUROC for diagnosing fatty liver was 0.809 in men and 0.791 in women. Abdominal aortic calcification effectively predicted a high coronary artery calcium score, with AUROCs of 0.837 in men and 0.946 in women. In addition, the AUROCs for detecting class II sarcopenia and low bone density [T-scores <–2.5, based on dual-energy X-ray absorptiometry (DXA)] were notably high, with 0.900 in men and 0.882 in women for sarcopenia, and 0.905 in men and 0.918 in women for bone density.
**Conclusion:** The study highlights the significant role of automated CT-derived imaging markers in the early detection and management of cardiometabolic risks. Markers such as liver fat fraction, abdominal aortic calcification, and muscle area are effective in predicting various cardiometabolic conditions. These findings endorse the use of CT-derived markers in routine health screenings, offering a potential pathway to improved patient outcomes through early intervention and targeted care for cardiometabolic health risks.

### SS 10.5

### Assessment of steatosis and fibrosis in individuals with risk factors for non-alcoholic fatty liver disease: Consensus between quantitative US and multiparametric MRI

F. Pucciarelli, I. Nacci, B. Masci, M. Zerunian, D. De Santis, D. Caruso, A. Laghi*; Rome/IT*

**Purpose:** To evaluate US attenuation coefficient (AC) for measuring liver fat deposition and two-dimensional shear wave elastography (2D-SWE) for quantifying liver fibrosis, using MRI-proton density fat fraction (MRI-PDFF) and MR elastography (MRE) as references, in patients at risk for non-alcoholic fatty liver disease (NAFLD).
**Material and Methods:** In this study, patients at risk for NAFLD (diabetes or metabolic syndrome) were included, excluding those with secondary fat deposition causes. Participants underwent same-day liver quantitative US (QUS) and MRI, along with tests within 30 days. Liver groups (normal, steatosis, and fibrosis) were identified using MRI-PDFF and MRE. Diagnostic performance of AC and 2D-SWE for detecting liver fat and stiffness was assessed by area under the curve (AUC). Correlations between QUS biomarkers and MRI were analyzed using Spearman coefficient.
**Results:** 54 participants were included: 21 (38.9%) had MRI-PDFF≥5%, and 10 (18.,5%) had MRE ≥2,9kPa. AUC of AC for determining ≥ mild steatotic livers was 0.80 (95% confidence interval [CI]: 0,67-0,94). AUC of 2D-SWE for determining ≥ F1 liver fibrosis was 0.59 (95% CI: 0,40–0,79). AC had a sensitivity of 71,4% and a specificity of 87,9%, and 2D-SWE had a sensitivity of 60% and a specificity of 52,3%. AC did not correlate well with MRI-PDFF in assessing hepatic steatosis (r=0,43), and 2D-SWE did not correlate to MRE in evaluating liver fibrosis (r=0,39).
**Conclusion:** Our preliminary results show that QUS biomarkers are more accurate for screening mild hepatic steatosis than fibrosis in patients with risk factors for NAFLD, but do not accurately discriminate the different degrees of steatosis and fibrosis.

### SS 10.6

### Normative values for liver ultrasound shear wave elastography, shear wave dispersion and attenuation imaging in a pediatric cohort during free breathing

D. Sarovic, I. Cetinic, N. Ekvall, K. Lagerstrand, C. de Lange, H. Hebelka; *Gothenburg/SE*

**Purpose:** To report on normative values of liver US shear wave elastography (SWE), shear wave dispersion (SWD) and attenuation imaging (ATI), reflecting hepatic elasticity, viscosity, and steatosis, respectively, in children.
**Material and Methods:** Seventy-one children, with no known liver disease, referred for an US-guided kidney biopsy were consecutively enrolled. Individuals with body mass index (BMI)>25 and measures with interquartile range/median>0.30 were excluded. SWE, SWD and ATI were measured with multimodal sampling during free breathing, fasting >4h and under general anesthesia (n=60) and/or while awake (n=42). We investigated the correlation of SWE, SWD and ATI to anesthesia, sex, age, and BMI.
**Results:** The mean age and BMI were 10.8 years (range 2.3–17.8) and 18.4 range (12.8–25.0), respectively; 49% were boys. Averages and standard deviations for the three markers were SWE 4.86±1.05 kPa, SWD 11.92±1.50 m/s/kHz, and ATI 0.56±0.09 dB/cm/MHz. There was no difference between sexes (p>0.15), effect of anesthesia (p>0.32), and between age groups (0–5, 5–12, 12–18 years; p>0.15). A moderate correlation was found between BMI and SWD (R=−0.43, p=0.006), while no other significant correlations were identified between US-based biomarkers and BMI or age (R<0.31, >0.08).
**Conclusion:** Normative values for SWE, SWD and ATI during free breathing in a pediatric cohort are presented. Other than a weak negative correlation between BMI and SWD, the lack of significant associations implies that these normative values can be applied across ages and sexes. Future studies are strongly encouraged to determine normative values for these US biomarkers in a large-scale population-based cohort with healthy children.

### SS 10.7

### Multiparametric MRI as a diagnostic tool for metabolic dysfunction-associated steatotic liver disease

N.P. Wassenaar[1], K.C. van Son[1], M.A. Troelstra[1], S. Driessen[1], A. Mak[1], E. Shumbayawonda[2], M. Nieuwdorp[1], M. Doukas[3], J. Verheij[1], A.J. Nederveen[1], O.J. Gurney-Champion[1], A.G. Holleboom[1]; *[1]Amsterdam/NL, [2]Oxford/UK, [3]Rotterdam/NL*

**Purpose:** Metabolic dysfunction-associated steatotic liver disease (MASLD) ranges from isolated steatosis to metabolic dysfunction-associated steatohepatitis (MASH), leading to fibrosis and cirrhosis. The reference standard for diagnosis and monitoring is a liver biopsy. However, its invasiveness and potential of sampling error limit its usability, indicating a need of non-invasive alternatives. Hence, we propose multiparametric MRI as an alternative.
**Material and Methods:** Patients [body mass index (BMI)≥25 kg/m$^2$, without excessive alcohol use] with US- and/or vibration-controlled transient elastography-confirmed hepatic steatosis, elevated aspartate transaminase (AST) and/or alanine transaminase (ALT) levels were included. MR elastography (MRE), intravoxel incoherent motion (IVIM), proton density fat fraction (PDFF), and corrected T1 (cT1) and T2* scans were obtained. Blood samples were collected and an US-guided liver biopsy was performed. Liver samples were steatosis, activity, and fibrosis (SAF)-scored in tandem. Patients were classified as having isolated steatosis or MASH. Spearman's rank correlation coefficient was computed between MRI parameters, blood markers, and individual components of the SAF-scores. Single- and multivariate logistic regression was performed on the six variables with the highest correlation coefficients to determine their ability to diagnose MASH.
**Results:** 26 patients with isolated steatosis and 49 with MASH were included. PDFF, MRE stiffness, IVIM diffusion, IVIM perfusion fraction, cT1 and AST gave the highest correlation coefficients for individual components of the SAF-scores. cT1 had the highest area under the receiver operating characteristic (AUROC) (0.79) for diagnosing MASH. Combining cT1, AST and MRE stiffness results in the highest diagnostic performance (log-odds=16.267-0.014*cT1-0.051*AST-1.518*MREstiffness) with AUROC of 0.86 and cross-validated sensitivity and specificity of 88% and 78%, respectively.
**Conclusion:** cT1 outperforms other MRI parameters in diagnosing MASH, and its performance increased when combined with AST and MRE stiffness.

### SS 10.8

### Quantification of gadoxetic acid-enhanced MRI enables the detection of metabolic dysfunction-associated steatohepatitis

S. Alsaqal[1], E. Johansson[2], P. Hockings[2], H. Ahlström[1], F. Rorsman[1], J. Vessby[1], C. Ebeling Barbier[1]; *[1]Uppsala/SE, [2]Mölndal/SE*

**Purpose:** There is a need for non-invasive biomarkers to differentiate between metabolic dysfunction-associated steatohepatitis (MASH) and metabolic dysfunction-associated steatotic liver (MASL) within the spectrum of metabolic dysfunction-associated steatotic liver disease (MASLD), also known as non-alcoholic fatty liver disease. Gadoxetic acid-enhanced MRI (GA-MRI) has the ability to quantify hepatocellular function and potentially identifying dysfunction. The purpose of this study was to evaluate the ability of various parameters obtained using GA-MRI to differentiate between MASH and MASL and between fibrosis stages and to assess the repeatability.
**Material and Methods:** Sixty-eight participants with biopsy-proven MASLD (40 men and 28 women; mean 54.5 years; 53 with MASH and 15 with MASL) were prospectively included. Contrast enhancement index (CEI), relative enhancement (RE), and Δrelative enhancement (ΔRE) were calculated using signal intensity data from GA-MRI sequences before and 20 minutes after the intravenous injection of GA. Thirty participants underwent a second MRI within 2–4 weeks to assess the repeatability.
**Results:** CEI, RE, and ΔRE could differentiate MASH from metabolic dysfunction-associated fatty liver (MAFL) with an area under the receiver operating characteristic (AUROC) of 0.68, 0.73, and 0.78, respectively. These parameters were also correlated to the histopathological activity grade (0–4) with Spearman's correlation coefficient of −0.30, −0.34, and −0.39, respectively, and to the histopathological fibrosis stage (0–4) with Spearman's correlation coefficient of −0.30, −0.29, and −0.28, respectively. The ICC for repeatability of these parameters ranged between 0.83 and 0.88.
**Conclusion:** MASH can be differentiated from MAFL, and fibrosis stages can be detected using highly repeatable parameters obtained using a routine clinical protocol of GA-MRI.

### SS 10.9

### Evaluation of liver fibrosis with shear wave elastography

M. Farasat, A. Kale, G. Pekindil, S. Tarhan, S. Ayhan; *Manisa/TR*

**Purpose:** Comparing point shear wave elastography (pSWE) and auto-pSWE values in the liver with the pathological fibrosis stage.
**Material and Methods:** Patients who applied for liver biopsy between October 2022 and September 2023 were included in the study. Before the biopsy, pSWE (10 measurements) and auto-pSWE (15 areas in a single measurement) measurements were made in kPa. Ishak scoring was used in pathological staging. Elastography values were compared with the Ishak stage.
**Results:** 35 patients, 20 men and 15 women, with an average age of 45 (20–69) years were included in the study. Elastography median value of all 35 patients was calculated as 4.3kPa (2.7–26.5) in pSWE and 5.3kPa (2.8–30) in auto-pSWE. pSWE and auto-pSWE values were correlated (r=0.927, p<0.001). Fibrosis stage was correlated with pSWE (r=0.680, p< 0.001) and auto-pSWE (r=0.691, p<0.001). In pSWE, the cutoff value, sensitivity, and specificity [area under the curve (AUC)] were 5.2, 76.9%, and 86.4% (0.874) for stage 0<; 5.2, 83.3%, and 87% (0.926) for stage 1<; 6.4, 72.7%, and 95.8% (0.919) for stage 2<; 8.1, 83.3%, and 93.1% (0.948) for stage 3<; and 10.2, 75%, and 96.8% (AUC 0.911) for stage 4<, which were calculated, respectively (p< 0.001). In auto-pSWE, the cutoff value, sensitivity, and specificity were 5.4, 76.9%, and 86.4% (0.885) for stage 0<; 5.4, 83.4%, and 86.9% (0.906) for stage 1<; 6.5, 81.8%, and 91.7% (0.883) for stage 2<; 8.6, 83.3%, and 89.7% (0.954) for stage 3<; and 9.7, 75%, and 96.8% (0.960) for stage 4<, respectively (p< 0.001).
**Conclusion:** pSWE and auto-pSWE values are correlated with each other and are useful in predicting the stage of pathological fibrosis non-invasively. Auto-pSWE provides ease of application in routine examination as it can take 15 measurements at a time.

### *SS 10.10*

### Assessment of hepatic and myocardial fibrosis in patients with sickle cell anemia using MRI

S. Basaran, K. Esen, A. Akdeniz, H.H. Yüksek, Y. Balcı; *Mersin/TR*

**Purpose:** This study aims to detect hepatic and myocardial fibrosis in sickle cell disease (SCD) using mapping techniques.

**Material and Methods:** 32 patients (average age: 37 years, no known comorbidities) and 12 healthy volunteers were prospectively evaluated. Native T1–T2–T2* maps were generated for both patient and control groups. In addition, post-contrast T1 map images were obtained for the purpose of calculating the extracellular volume (ECV) value.

**Results:** In the T2* mapping examination, while cardiac iron accumulation was not observed, iron deposition in the liver was detected in 16 patients. When patients with iron accumulation were excluded, the average liver T1 relaxation time was measured as 626.56±61.45 and the ECV value as 40.4±7.24; these values were found to be statistically significantly higher compared to the control group ($p=0.000$). Furthermore, in the patient group, the average myocardial T1 time was measured as 1030.91 ms and the ECV value as 31.73±3.1, also statistically significantly higher than the control group ($p=0.002$).

**Conclusion:** Our study revealed an increase in liver and myocardial native T1 and ECV values in patients with SCD compared to the control group. The elevation in native T1, indicating an increase in conditions such as edema and fibrosis, along with the correlated rise in ECV values, a reliable marker of collagen content in the tissue, signifies the development of liver and myocardial fibrosis in individuals with SCD. We believe that mapping methods capable of simultaneously demonstrating iron accumulation through T2* mapping and fibrosis through T1 and ECV measurements can be employed in standard screening and follow-up, potentially reducing the need for biopsy.

09:00 - 10:30 Room J1

*Scientific Session SS 11*
### Oncology

### SS 11.1
### Whole-body diffusion-weighted MRI in the diagnostic workup of patients with adenocarcinoma of unknown primary: A prospective pilot study

J. Willemse, M. Lahaye, P. Snaebjornsson, S. Marchetti, M. Vollebergh, L. van Golen, W. Vogel, R. Beets-Tan, D. Lambregts; *Amsterdam/NL*

**Purpose:** This prospective diagnostic pilot study aimed to investigate the potential added benefit of whole-body MRI (WB-MRI) with diffusion-weighted imaging (DWI) in the diagnostic workup of adenocarcinoma of unknown primary (ACUP) patients.
**Material and Methods:** From January 2022 to December 2023, the option of WB-MRI was added as an adjunct diagnostic tool to the routine clinical workup, including CT, fluorodeoxyglucose positron emission tomography (FDG-PET)/CT, lab tests and biopsies for ACUP patients in our institution. The choice of whether to perform an MRI was discussed by a multidisciplinary team and guided by all available clinical information (e.g. if the primary tumour could likely be suspected to be located within the abdomen/pelvis). We analyzed the impact of WB-MRI in terms of primary tumour identification and detection of additional metastatic sites.
**Results:** WB-MRI was performed on 29 ACUP patients, revealing potential primary tumour locations in 24% (7/29) of the cases not diagnosed by previous CT and/or FDG-PET/CT. These locations included bile ducts (n=2), ovary (n=1), appendix (n=1), duodenum (n=1), pancreas (n=1), and breast (n=1). In 6 of these 7 cases, the WB-MRI diagnosis aligned with and supported the final diagnosis, established by integrating clinicopathological data with whole-genome sequencing. In addition, WB-MRI identified extra metastatic sites in 24% (7/29) of the patients, including peritoneal (n=3), bone (n=1), kidney (n=1), testicular (n=1), and pancreatic (n=1) metastases.
**Conclusion:** This study demonstrates the potential added value of WB-MRI in the search for the underlying primary tumour in the complex diagnostic workup of patients with disseminated adenocarcinoma of unknown primary.

### SS 11.2
### Searching for the primary tumour in patients with cancer of unknown primary using fluorodeoxyglucose positron emission tomography/CT: A systematic review and individual patient data meta-analysis

J. Willemse[1], D. Lambregts[1], P. Snaebjornsson[1], S. Marchetti[1], M. Vollebergh[1], L. van Golen[1], T. Sivakumaran[2], R. Beets-Tan[1], M. Lahaye[1]; [1]*Amsterdam/NL*, [2]*Melbourne/AU*

**Purpose:** Patients with cancer of unknown primary (CUP) present with disseminated cancer, where, despite standard diagnostic evaluations, the primary tumour cannot be determined. This systematic review and individual patient data (IPD) meta-analysis aims to analyze the performance of fluorodeoxyglucose positron emission tomography (FDG-PET)/CT in detecting primary tumours and assess whether the most dominant metastatic site influences this performance.
**Material and Methods:** A systematic literature search (January 2005–November 2022) focused on FDG-PET/CT's diagnostic performance for primary tumour detection in CUP patients was performed. Individual patient data were retrieved from the original articles or via the corresponding authors and patients were grouped by dominant metastatic site. Diagnostic performance of FDG-PET/CT for primary tumour detection was analyzed in each group.
**Results:** Involving 1759 patients from 31 studies, the largest subgroup had dominant bone metastases (n=592), followed by liver (n=364), lymph nodes (n=315), brain (n=314), peritoneum (n=70), lung (n=55), and soft tissue (n=18) metastases, with a small group of other/undefined metastases (n=31). Primary tumour detection rates for FDG-PET/CT were 75% (for patients with predominant brain metastases), 56% (liver-dominant), 52% (lung-dominant), 48% (bone-dominant), 40% (soft tissue-dominant), 38% (peritoneal-dominant), and 37% (lymph node-dominant). Primary tumours included 527 lung, 294 GI, 136 urogenital, and 120 miscellaneous tumours, and remained unidentified in 578 (33%) of patients.
**Conclusion:** FDG-PET/CT has variable detection rates to identify the underlying primary tumour in CUP, depending on the dominant pattern of a metastatic disease with better performance for patients presenting predominantly with brain, liver, lung, or bone metastases.

### SS 11.3
### Comparing the predictive precision of MRI radiologic and laparoscopic peritoneal carcinomatosis index assessment for surgical prognosis

S.K. Kale, S.P. Somashekhar, A. Fernandes, L. Sathyanarayan; *Bangalore/IN*

**Purpose:** Peritoneal carcinomatosis, originating primarily from colorectal, gastric, and gynecological cancers, poses diagnostic challenges due to its diverse etiology, including benign conditions and inflammatory diseases. Imaging modalities play a pivotal role, with MRI currently being employed. MRI, albeit sensitive, necessitates scrutiny. This study aims to meticulously assess the predictive accuracy of preoperative MRI in determining the peritoneal cancer index (PCI) compared to intraoperative PCI tabulation.
**Material and Methods:** In a prospective study of 100 consecutive patients with primary peritoneal carcinomatosis eligible for cytoreductive surgery, preoperative MRI was followed by staging laparoscopy. Retrospective analysis of MRI data involved PCI determination, subsequently compared with intraoperatively tabulated PCI. Patients were stratified into small (PCI 0–9), moderate (PCI 10–20), and large volume (PCI > 20) categories. Anatomical site scores from MRI were juxtaposed with surgical findings.
**Results:** MRI accurately categorized tumor volume in 90% of the patients, demonstrating particular efficacy in moderate and large volume tumors. Median PCI at surgery was 26, aligning closely with the MRI-derived median of 24. Notably, MRI exhibited per-site sensitivity of 95%, specificity of 70%, and an overall accuracy of 88%.
**Conclusion:** Preoperative MRI, especially proficient in moderate and high PCI cases, adeptly predicts PCI, facilitating meticulous planning for cytoreductive surgery and hyperthermic intraperitoneal chemotherapy (HIPEC). This nuanced assessment underscores MRI's pivotal role in refining surgical strategies for optimal patient outcomes.

### SS 11.4
### Utility of clinical and MRI imaging parameters for prediction and monitoring of response to capecitabine and temozolomide in patients with liver metastases of neuroendocrine tumors

M.K. Ingenerf, C. Auernhammer, M. Winkelmann, S. Mansournia, J. Ricke, A.-K. Lohse, F. Berger, C. Schmid-Tannwald; *Munich/DE*

**Purpose:** To investigate how clinical and multiparametric MRI parameters predict and monitor (capecitabine and temozolomide) CAPTEM therapy response in neuroendocrine tumor (NET) patients.
**Material and Methods:** This retrospective study (n=44) evaluated CAPTEM therapy response in neuroendocrine liver metastases (NELM) patients. Among 33 monitored individuals, pre- and post-treatment MRI data [size, apparent diffusion coefficient (ADC) values, and signal intensities] and clinical parameters [chromogranin A (CgA) and Ki-67%] were analyzed, using progression-free survival (PFS) as the reference. Responders (R) were defined as those with PFS > 6 months.
**Results:** The majority of patients (75%) were male, having G2 tumors (76%) from the pancreas (84%). Median PFS was 5.7 months, while overall survival reached 25 months. Non-responders (NR) had higher Ki-67 in primary tumors (16.5 vs. 10%, p=0.01) and increased hepatic burden (20% vs. 5%, p=0.007). Post-treatment, NR showed elevated CgA, while R displayed a mild decrease. Significant ADC differences were observed, with NR having decreased ADCmin (-23%) and liver-adjusted ADCmean/ADCmean-liver (-16%), whereas R experienced increases in ADCmin (50%) and ADCmean/ADCmean-liver (30%). Receiver operating characteristic (ROC) analysis identified the highest area under the curve (AUC) (0.76) for Δ ADC mean/ADCmean-liver, with a cutoff of < 6.9 (76% sensitivity, 75% specificity). Combining Δ Size-NELM and Δ ADCmin achieved the best balance (88% sensitivity, 60% specificity), surpassing Δ Size-NELM alone (69% sensitivity, 65% specificity). Kaplan–Meier analysis indicated significantly longer PFS for Δ ADCmean/ADCmean-liver < 6.9 (p=0.024) and Δ Size NELM > 0% + Δ ADCmin < −2.9% (p=0.021).
**Conclusion:** Survival analysis highlights the need for tailored response criteria, demanding a comprehensive assessment of CgA, ADC values, and tumor size to effectively monitor CAPTEM response in hepatic metastasized NETs.

### SS 11.5

### Early prediction of the efficacy of $^{177}$Lu-DOTATATE in patients with metastatic neuroendocrine tumours

A. Bando-Delaunay, O. de Rycke, P. Leclerc, O. Hentic, A. Allouch, J. Cros, V. Rebours, P. Ruszniewski, R. Lebtahi, L. de Mestier, M. Ronot*; Clichy/FR*

**Purpose:** Peptide receptor radionuclide therapy (PRRT) has a major place in the treatment of metastatic neuroendocrine tumours (NETs). Its efficacy is generally evaluated after four cycles (C), with no early predictor identified to date. During each PRRT cycle, $^{177}$Lu-related gamma emissions can be measured by post-PRRT scintigraphy. We explored whether the variation in tumor uptake on post-PRRT scintigraphy performed at C1 and C2 could predict the efficacy of PRRT.
**Material and Methods:** We studied all patients who received at least two cycles of PRRT (2013–2019) for metastatic NET. We calculated the C2/C1 ratio of liver metastases uptake (geometric mean of anterior and posterior uptakes) on post-PRRT scintigraphy. We explored the association between the C2/C1 ratio and the response evaluation criteria in solid tumors (RECIST)-defined response after C4 and progression-free survival (PFS).
**Results:** We included 80 patients (85 treatments) with small-bowel (57%) or pancreatic (26%) and G1 (31%) or G2 (65%) NET. The C2/C1 ratio was lower in responders (median 0.67 vs 0.94, p<0.0001). A ratio <0.85 best predicted tumour response [area under the curve (AUC) 0.81, p<0.001], which remained significant in multivariable analysis (OR 6.9, p=0.008). Median PFS was 21 months in the whole cohort, 26 months for a ratio <0.85 and 20 months for a ratio ≥0.85 (p=0.84). In multivariable analyses, a reduction in uptake between C2 and C1 (ratio<1) was significantly associated with a reduction in the risk of tumour progression (p=0.02).
**Conclusion:** The evolution of NET uptake between C1 and C2 is a simple tool for early prediction of PRRT efficacy. This biomarker, if confirmed prospectively, could help identify early the patients who may benefit from the treatment optimisation or change.

### SS 11.6

### Quantitative somatostatin receptor positron emission tomography/CT: A potential tool for predicting everolimus response in NET patients

M.K. Ingenerf, M. Winkelmann, J. Ruebenthaler, A.-K. Lohse, S. Mansournia, H. Karim, J. Ricke, C. Auernhammer, C. Schmid-Tannwald*; Munich/DE*

**Purpose:** This study aimed to assess $^{68}$Ga-DOTA-TATE (-TOC) positron emission tomography (PET)/CT quantitative parameters in monitoring and predicting everolimus response in neuroendocrine tumor (NET) patients with hepatic metastases (NELM).
**Material and Methods:** A retrospective analysis included 29 patients with 62 target lesions undergoing everolimus treatment and pre-therapy, and follow-up $^{68}$Ga-DOTA-TATE (-TOC) PET/CT scans. Response evaluation utilized progression-free survival (PFS) categorized as responders (R; PFS > 6 months) and non-responders (NR; PFS ≤ 6 months). Lesion size and density, along with standardised uptake value (SUV) in target lesions, liver, and spleen, were assessed. Tumor-to-spleen (T/S) and tumor-to-liver (T/L) ratios were calculated.
**Results:** PET/CT scans were acquired 19 days (IQ 69 days) pre-treatment and 127 days (IQR 74 days) post-starting everolimus. The overall median PFS was 264 days (95% CI: 134–394 days). R exhibited significant decreases in Tmax/Lmax and Tmean/Lmax ratios compared to NR (p=0.01). In univariate Cox regression, Tmean/Lmax ratio was the sole prognostic parameter associated with PFS (HR 0.5, 95% CI 0.28–0.92, p=0.03). Percentage changes in T/L and T/S ratios were significant predictors of PFS, with the highest area under the curve (AUC) for the percentage change of Tmean/Lmax (AUC=0.73). An optimal threshold of < 2.5% identified patients with longer PFS (p=0.003). No other imaging or clinical parameters were predictive of PFS.
**Conclusion:** This study highlights the potential of quantitative somatostatin receptor positron emission tomography (SSTR-PET)/CT in predicting and monitoring everolimus response in NET patients. Liver metastasis-to-liver parenchyma ratios outperformed size-based criteria, and Tmean/Lmax ratio may serve as a prognostic marker for PFS, warranting larger cohort investigation.

### SS 11.7

### Validation of the standardisation framework somatostatin receptor positron emission tomography reporting and data system 1.0 for neuroendocrine tumours using the novel somatostatin receptor-targeting peptide [18F]SiTATE

R.K. Ebner*; Munich/DE*

**Purpose:** Somatostatin receptor positron emission tomography (SSTR-PET)/CT using [68Ga]-labelled tracers is a widely used imaging modality for neuroendocrine tumours (NET). Recently, [18F]SiTATE has shown great potential due to its favourable clinical characteristics. We aimed to evaluate the reproducibility of SSTR reporting and data system (SSTR-RADS) 1.0 for structured interpretation and treatment planning of NETs using [18F]SiTATE.
**Material and Methods:** Four readers assessed [18F]SiTATE-PET/CT of 95 patients according to the SSTR-RADS 1.0 criteria at two different time points. Each reader selected and evaluated up to 5 target lesions per scan. Overall scan score and the decision on peptide receptor radionuclide therapy (PRRT) were considered. Inter- and intrareader agreement was determined using the intraclass correlation coefficient (ICC).
**Results:** Interreader agreement for identical target lesions (ICC≥85%), overall scan score (ICC≥90%) and decision to recommend PRRT (ICC≥85%) showed excellent agreement. However, significant differences were observed in recommending PRRT within ERs (p =0.020) and IRs (p =0.004). Compartment-based analysis demonstrated good to excellent interreader agreement for most organs (ICC≥74%), except for lymph nodes (ICC≥52%).
**Conclusion:** SSTR-RADS 1.0 represents an accurate and reproducible framework system for stratifying [18F]SiTATE-PET/CTs as an alternative for [68Ga]-labelled PET/CTs in NET-imaging. However, excellent interreader agreement on the overall scan score and the decision for PRRT was observed, and there were variations in PRRT recommendations, highlighting the complexity of such decisions, suggesting the need for a multidisciplinary input. Compartment-based assessments demonstrated excellent interreader agreement for the liver, soft tissue, and skeleton, with varying agreement for lymph nodes, emphasising the importance of functional imaging for small lesions.

### SS 11.8

### Radiomics and differential diagnosis of pancreatic tumors

E. Bardhi, F. Spoto, M. Todesco, B. Mascarin, F. Baratta, R. De Robertis, M. D'Onofrio*; Verona/IT*

**Purpose:** The aim of the study was to evaluate whether radiomics, through the analysis of the features obtained from the texture analysis of different pancreatic tumors, was able to predict the histotype; in particular, ductal adenocarcinomas and neuroendocrine tumors were compared.
**Material and Methods:** Contrast-enhanced CT scans of 193 patients were retrospectively reviewed and a total of 97 adenocarcinomatous lesions and 96 neuroendocrine lesions were analyzed. Furthermore, anamnestic data (smoking habits, diabetes, and jaundice) and clinical data (CA19-9, gastrin, serotonin, insulin, C-peptide, and alpha-feto protein) were evaluated. 107 features were extracted for the arterial and venous phases, and receiver operating characteristic (ROC) curves were constructed for the parameters that proved to have the highest area under the curve (AUC) for two groups, the first including all the lesions and the second including only the lesions smaller than 5 cm.
**Results:** The following differences in features were found to be statistically significant (p < 0.05). Not discriminating the dimensions: for the arterial phase, 16 first-order and 38 second-order features; for the venous phase, 10 first-order and 20 second-order features. Considering instead the lesions with axes less than 5 cm: for the arterial phase, 16 first-order and 52 second-order features; for the venous phase, 11 first-order and 36 second-order features.
**Conclusion:** Texture analysis of pancreatic pathology has demonstrated good predictability in defining the neuroendocrine histotype and is worthy of further investigation.

### SS 11.9

### Impact of a one-day multidisciplinary hepatobiliary consultation on diagnosis and time-to-treatment in a tertiary referral center

J. Grégory[1], G. Porrello[2], R. Sartoris[1], M. Dioguardi Burgio[1], J. Campinchi[1], A. Beaufrère[1], V. Paradis[1], M. Ronot[1], V. Vilgrain[1]; [1]Clichy/FR, [2]Palermo/IT

**Purpose:** To assess the impact of a rapid multidisciplinary diagnostic clinic for liver tumours (HOPE) on diagnosis and treatment delays.

**Material and Methods:** This is a retrospective study examining data collected prospectively from patients referred to the HOPE centre for suspected liver lesions (2017–2020). The primary endpoint was time-to-diagnosis (delay between initial pre-HOPE imaging and final diagnosis). Secondary endpoints included the number of corrected diagnoses, accurate diagnoses made by HOPE, and time-to-treatment (from HOPE to treatment).

**Results:** Four hundred and twenty-one patients were included, 233 without diagnostic hypothesis. The median (Q1–Q3) delay between initial imaging and the final diagnosis was 55 (28–119) days. The median delay between initial imaging and the HOPE consultation was 46 (23–108) days. Of 188 patients with a diagnostic hypothesis, 49 (26%) had their diagnosis changed. Additional tests were performed on the day of consultation for 347 patients (82%), including 111 USs, 186 MRIs, and 56 CT. The diagnosis proposed by HOPE was "certain" (n=281), "probable but requiring pathological" (n=106), "probable but requiring follow-up" (n=29), and "uncertain" (n=5). For 144 patients, a biopsy was performed. The median delay between HOPE and pathology results was 22 days (10–24). The final diagnosis was malignant for 107 patients and benign for 307. These final diagnoses included 41 different types, the most frequent being focal nodular hyperplasia (FNH) (121), hemangioma (90), HCC (48), cholangiocarcinoma (25), and metastases (22). One hundred and twenty-one patients underwent treatment after a median 49 (34–66) days.

**Conclusion:** A rapid multidisciplinary diagnostic clinic for liver tumours allows for precise diagnosis, with short time-to-diagnosis and treatment.

### SS 11.10

### Socioeconomic disadvantage is associated with higher risk body composition measures and mortality risk in a large heterogeneous adult population

M. Lee[1], B.E. Rush[1], J.W. Garrett[1], R.M. Summers[2], P.J. Pickhardt[1]; [1]Madison, WI/US, [2]Bethesda, MD/US

**Purpose:** To evaluate the relationship between socioeconomic disadvantage and CT-based body composition (BC) measures associated with cardiometabolic and mortality risk using fully automated artificial intelligence (AI) tools.

**Material and Methods:** In this retrospective cohort study, automated AI BC tools quantifying muscle (area and attenuation [HU]), abdominal fat (visceral [VAT], visceral-to-subcutaneous ratio [VSR]), abdominal aortic calcium, and bone mineral density (BMD) were applied to abdominal CT examinations in adults undergoing abdominal CT at a single academic institution. Patients were partitioned into 5 socioeconomic groups based on area deprivation index (ADI) rank at the census block group level (national rank, 1–100): 1 being the lowest and 100 being the highest level of "disadvantage." One-way analysis of variance compared means across groups. Survival analysis was performed, and Kaplan–Meier curves were generated.

**Results:** 92,970 adult patients (mean age, 52 years; 49,011:43,959 F:M) underwent abdominal CT from January 2001 to December 2021. Sex-specific differences were observed for all BC measures ($p < .05$). The most disadvantaged group had higher risk BC measures. Mean differences between the most and least disadvantaged groups (ADI > 80) were: muscle area=4 $cm^2$, muscle HU=−6 HU, aortic calcium (Agatston)=303, VAT=22 $cm^2$, VSR=0.1, BMD=-8HU (all $p < .05$). 5-year survival differences were observed across ADI quintiles with greatest curve separation between the most and least disadvantaged groups ($p < .05$). Higher ADI was associated with increased mortality risk—quintile 5 (HR 1.3) vs. quintile 2 (HR 0.97) ($p < .05$).

**Conclusion:** Socioeconomic disadvantage is associated with higher risk body composition measures and mortality risk in a large heterogeneous adult patient population.

**09:00 - 10:30** **Room J2**

*Scientific Session SS 12*

### Chronic liver disease and cirrhosis

### SS 12.1

### MRI-based quantitative test for physical frailty - Interim results from the Swedish prospective multi-center liver cirrhosis cohort study ACCESS-ESLD

M.F. Forsgren[1], W. Balkhed[1], P. Nasr[1], D. Sjögren[2], A. Cederborg[3], M. Holmberg[1], J. Linge[1], M. Rejler[4], H. Stjernman[4], S. Kechagias[1], N. Dahlström[1], O. Dahlqvist Leinhard[1], M. Ekstedt[1]; [1]Linköping/SE, [2]Eksjö/SE, [3]Gothenburg/SE, [4]Jönköping/SE

**Purpose:** Frailty and sarcopenia are associated with liver cirrhosis outcomes. Frailty may be assessed with liver frailty index (LFI). Sarcopenia is typically assessed by imaging-based muscle measurements, e.g. L3 skeletal muscle index (L3-SMI). An MRI-based assessment (muscle assessment score [MAsS]) combining sex-adjusted thigh muscle fat infiltration (MFI) and volume z-score (MVZ) has been developed to describe muscle health. The aim was to assess how MAsS and L3-SMI predicts frailty in patients with liver cirrhosis.

**Material and Methods:** Patients were prospectively enrolled at three Swedish hospitals. Clinical assessment and 8-min WB-MRI were performed on the same day. Muscles were measured using AMRA® Researcher. LFI was graded as 'frail', 'prefrail', 'less robust', and 'robust'. ANOVA (with Bonferroni post-test) and Spearman correlation (with LFI as continuous variable) were used.

**Results:** The first 76 patients (46 males; 29.5±6.3 kg/$m^2$; 66±10 y.o.; mainly steatotic liver disease) were included. ANOVA showed differences between frailty groups for MFI (p=0.004); MFI was higher in frail versus robust (+3.70pp**) and less robust (+2.98pp*). Muscle volumes were not significant (MVZ, p=0.12; L3-SMI p=0.64). MFI correlated with LFI (ρ=0.43, p=0.001). Muscle volumes were not significant (MVZ, ρ=−0.23, p=0.10; L3-SMI, ρ=−0.04, p=0.79).

**Conclusion:** MFI correlated with LFI and was higher in frail patients, 50% higher than normal. L3-SMI was not related to frailty. This indicates that MAsS may be used as a quantitative MRI-based test for frailty in patients with liver cirrhosis.

### SS 12.2

### Noninvasive assessment of liver segmental volumes and its relationship with 5-year prognostication

D. Catucci, J. Hrycyk, N.F. Lange, V.C. Obmann, A. Berzigotti, L. Ebner, J.T. Heverhagen, A. Christe, A.T. Huber; Bern/CH

**Purpose:** This study aimed to analyze the predictive value of liver segmental volume and attenuation ratio (LSVR/LSVAR), and caudate to right lobe ratio (CRL-R) for chronic liver disease (CLD) on routine abdominal CT scans and to predict the 5-year probability of transplant-free survival and first hepatic decompensation.

**Material and Methods:** This retrospective study included 108 patients without CLD (noCLD-group; n=108), as well as 98 patients with biopsy-proven CLD. All patients underwent abdominal CT scans between 03/2015 and 08/2017. Patients with CLD were divided into three groups: early CLD (F0–F2; eCLD; n=40), advanced CLD (F3–F4; aCLD; n=20), and aCLD with clinically significant portal hypertension according to the BAVENO VII consensus (aCLDPH; n=38). CRL-R, LSVR, and LSVAR were compared between the groups using Kruskal–Wallis test and receiver operating characteristic (ROC) analysis to determine cutoff values. The 5-year transplant-free survival and first hepatic decompensation were assessed by Kaplan–Meier curve analysis.

**Results:** CRL-R, LSVR and LSVAR differed significantly between all the groups (p<0.001). A CRL-R cutoff value of >0.93 predicted aCLD with a sensitivity of 69% and a specificity of 78%, while LSVR >0.37 had a sensitivity of 68% and a specificity of 80%. Patients with both CRL-R >0.99 and LSVR >0.37 had the lowest probability of 5-year transplant-free survival (46%) and the lowest probability of a decompensation-free 5-year course (75%), which was significantly different (p<0.001) from patients with both CRL-R <0.99 and LSVR <0.37 (86%; 98%).

**Conclusion:** LSVR/LSVAR and CRL-R on routine abdominal CT scans showed a high predictive value for CLD and 5-year outcome prognostication.

### SS 12.3

### Comparison of MRI liver function imaging scores in determining the severity of chronic liver disease with clinical scoring systems of model for end-stage liver disease, Child–Turcotte–Pugh score, and albumin-bilirubin index

E. Aydin[1], O. Sahin[1], H. Sahin[1], G. Akpinar[1], S. Sahin[2]; [1]Izmir/TR, [2]Elazıg/TR

**Purpose:** To compare the model for end-stage liver disease (MELD) score, Child–Turcotte–Pugh score (CTPS), and albumin-bilirubin index (ALBI) with the liver function imaging score (LFIS) derived from contrast-enhanced MRI using gadoxetic acid in terms of the severity of liver disease in patients with chronic liver disease (CLD) and/or liver cirrhosis (LC), with a control group.

**Material and Methods:** Based on clinical and laboratory findings, diseases that deteriorate liver function, such as CLD and LC, are categorized using the CTPS, ALBI, and MELD scoring systems. MRILFIS was defined in 2020. According to this scoring, the liver contrast enhancement, portal vein signal, and bile duct excretion are scored between 0 and 2 to determine the overall score in the hepatobiliary phase T1A sequence. Student t test and Mann–Whitney U test were used for statistical analysis. Spearman correlation coefficient was used for correlation analysis and p< 0.05 was considered significant.

**Results:** In the study, the patient group included 38 cases (28 women, 10 men), and the control group included 47 cases (16 female, 31 male). While the average age of the patient group was 62.42±14.32 years, the average age of the control group was 57.81±15.05 years. While the average liver volume was 1474.05±380.97ml in the patient group, in the control group, it was 1672.02±424.83ml (p=0.028). LFIS was found to be significantly different between the patient and control groups (p=0.035). LFIS showed a negative correlation with CTPS and MELD; it was found that there was no statistically significant correlation with ALBI (<0.035, <0.001, 0.404, respectively). When the total score was evaluated by two observers, interobserver agreement was excellent (K=0.826, p<0.001).

**Conclusion:** LFIS was correlated with CTPS and MELD, but was not statistically correlated with ALBI score and liver volume. LFIS can be used safely to evaluate liver function regardless of experience.

### SS 12.4

### Comparison of the efficacy of the gadoxetic acid MRI-derived relative enhancement index and functional liver imaging score in predicting liver function: Validation with albumin-bilirubin grade

U. Eryuruk, S. Aslan, M. N. Tasdemir, H. I. Karasu; *Giresun/TR*

**Purpose:** To compare performance of the relative enhancement index (REI) derived from gadoxetic acid (GA)-enhanced MRI with that of the functional liver imaging score (FLIS) in estimating liver function among patients with chronic liver disease (CLD) or liver cirrhosis (LC) by validating them with the albumin–bilirubin (ALBI) score.

**Material and Methods:** We retrospectively examined 443 patients who were diagnosed with LC or CLD and underwent GA-enhanced MRI. The enhancement ratio (ER) was calculated using the formula ER = [hepatobiliary phase liver signal intensity (SI HBP20) − precontrast liver signal intensity (SI Pre)]/SI Pre. The REI was calculated using the formula REI = liver volume × ER. FLIS is scored from 0 to 6 as recently defined. Receiver operating characteristic (ROC) curve analysis was performed to determine the optimal cutoff values of REI and FLIS in differentiating between ALBI scores. Spearman's rank correlation was used to evaluate correlations.

**Results:** The ROC curve analysis showed that to predict ALBI grade 1, the optimal cutoff of REI was 890, and to predict ALBI, grade 3 was 501. REI showed the best performance in predicting ALBI grade 1 and grade 3, with 95.49%–99.32% accuracy, 96.92%–100% sensitivity, and 84.91%–99.29% specificity, respectively. REI showed a better correlation with the ALBI grade than FLIS (r: − 0.928 versus − 0.882).

**Conclusion:** REI showed a very strong correlation with the ALBI grades. It outperformed FLIS in predicting the ALBI grades, indicating its potential as a radiologic tool comparable to or better than FLIS in predicting liver function.

### SS 12.5

### Quantitative and semiquantitative parameters of gadoxetic acid-enhanced MRI to predict acute-on-chronic liver failure in advanced chronic liver disease

A. Ba-Ssalamah, S. Poetter-Lang, N. Bastati-Huber, S. Pochepnia, M. Schernthaner, L. Balcar, T. Reiberger, M. Mandorfer, L. Beer; *Vienna/AT*

**Purpose:** To investigate the ability of functional liver imaging score (FLIS), as well as its quantitative imaging components, including the relative liver enhancement (RLE), relative enhancement ratio of the biliary system (REB) and liver-to-portal vein contrast ratio (LPC), to predict acute on chronic liver failure (ACLF) in hospitalized and non-hospitalized advanced chronic liver disease (ACLD) patients.

**Material and Methods:** We included 322 ACLD patients who had gadoxetic acid-enhanced liver MRI. The FLIS was calculated by summing the qualitative points (0–2): hepatic enhancement; biliary excretion; and portal vein signal intensity. The RLE, REB and LPC were computed. Patients were stratified into three clinical groups: non-advanced CLD, compensated-advanced CLD (cACLD), and decompensated-advanced CLD (dACLD). We further sub-stratified patients according to whether their MRI was an elective outpatient exam or performed during a liver-related hospital admission. The predictive value of FLIS, as well as RLE, REB and LPC for ACLF development were investigated using log-rank test and Cox regression analysis.

**Results:** The FLIS was an independent risk factor for ACLF development in non-hospitalized ACLD patients ([a]HR: 2.86, P=0.025) and in hospitalized dACLD patients (aHR: 3.9; 95%CI: 1.08–14.08; P=0.04). Similarly, the FLIS was an independent risk factor for transplant-free mortality in hospitalized dACLD patients (aHR: 2.49; P=0.02) and also showed a trend in non-hospitalized ACLD patients (aHR: 2.01; 95%CI: 0.96–4.22, P=0.07). RLE, REB and LPC could distinguish between cACLD and dACLD patients (P<0.001) but failed to predict ACLF.

**Conclusion:** FLIS is a simple imaging biomarker for predicting acute-on-chronic liver failure and liver-related mortality in advanced chronic liver disease patients and outperformed the quantitative parameters.

### SS 12.6

### Determination of the relationship between liver enhancement ratio and liver function tests in gadoxetic acid-enhanced MRI in patients with chronic liver disease and cirrhosis

B. Akdal Dolek, H. Tekdemir, S. Sahin, R. S. Okten; *Ankara/TR*

**Purpose:** To evaluate the liver enhancement ratio (LER) on gadoxetic acid-enhanced MRI in patients with liver cirrhosis and its relationship with albumin–bilirubin (ALBI) grade and Child–Pugh and model for end-stage liver disease (MELD) scores.

**Material and Methods:** We retrospectively analyzed 92 patients between January 2022 and December 2023 who underwent gadoxetic acid-enhanced MRI. In all patients, liver signal intensity (SI pre) in precontrast fat-suppressed T1 weight series and liver signal intensity (SI HBP20) in hepatobiliary phase images were measured by manually placing five regions of interest (ROI) at the same locations in the right and left lobes. LER was calculated with the formula LER = (SI HBP 20 – SI pre) / SI pre.

**Results:** In the study, 36 cirrhotic and 56 healthy participants were evaluated. The LER was statistically significantly lower in the cirrhosis patient group compared to the control group (p<0.001). As a result of the receiver operating characteristic (ROC) analysis, the LER cutoff value between the two groups was 1.14 [area under the curve (AUC): 0.84]. A significant statistical difference was found between the LER in Child A–Child B and Child A–Child C groups (p: 0.46, p: 0.27). In addition, a statistically significant negative correlation was found between LER and MELD score in the cirrhosis patient group (p: 0.02). When the cirrhosis patient group was divided into three groups according to ALBI score, a significant statistical difference was detected between the LER in the groups (p<0.001).

**Conclusion:** LER showed statistically significant differences between liver function parameter groups. Hence, the diagnosis of chronic liver disease can be predicted with the quantitative data obtained from gadoxetic acid-enhanced MRI examination.

Scientific Sessions

Authors' Index

### SS 12.7

### Diagnostic accuracy of acoustic radiation force impulse in identifying graft fibrosis in patient post-liver transplantation: Systematic review and meta-analysis

A. Aljumaa, R. Elmokattaf, J. Aljumaa, J. Hejazi; *Riyadh/SA*

**Purpose:** To synthesize the currently available evidence on the diagnostic accuracy of acoustic radiation force impulse (ARFI) in identifying graft fibrosis in liver recipients.

**Material and Methods:** We searched three databases (PubMed, Embase, and Web of Science) for all studies assessing the accuracy of ARFI in identifying graft fibrosis in liver recipients. Two reviewers independently screened all the identified papers. Only cohort, cross-sectional, and clinical trial studies were eligible for inclusion. The Quality Assessment of Diagnostic Accuracy Studies (QUADAS)-2 tool was used for the quality assessment of the included studies. The area under the receiver operating characteristics (AUROC) were meta-analyzed using the random-effect inverse-variance method.

**Results:** 8 studies evaluating 636 liver recipients were included in the analysis. The imaging protocols of the studies were heterogeneous. ARFI has shown a good diagnostic performance in identifying graft fibrosis when compared to the gold standard of liver biopsy, with a pooled AUROC of 0.80 (95% CI, 0.76–0.85), pooled sensitivity of 80% (95% CI, 0.71–0.87), and pooled specificity of 77% (95% CI, 073–0.82).

**Conclusion:** ARFI has demonstrated favorable diagnostic performance and can be utilized as a non-invasive method to detect graft fibrosis in liver recipients. By employing this diagnostic instrument, one can circumvent the adverse effects that are usually associated with liver biopsies.

### SS 12.8

### Body fat composition and liver iron concentration: Unveiling the intricate correlation in hemochromatosis through advanced MRI techniques

M. Pušeljić, N. Ahmadova, N. Watzinger, F. Hohenberg, A.-K. Kaufmann-Bühler, M. Fuchsjäger, E. Talakić; *Graz/AT*

**Purpose:** To evaluate the correlation between body fat composition, liver iron concentration (LIC) and standard laboratory parameters in patients with hemochromatosis.

**Material and Methods:** This is a retrospective analysis of MRI scans of patients who underwent LIC quantification using a 3T device from 2005 to 2023. This analysis utilized Siemens Healthineers LiverLab, assessing proton density fat fraction and R2* relaxation times across the entire liver volume and a region of interest (ROI) in the right lobe. In addition, a single-voxel-based spectroscopic method was used for R2 relaxation times. Iron dry weight (IDW) was estimated using the equation: 0.314xR2*–0.96. The percentages of total fat (TF), subcutaneous fat (SF), intermuscular fat (IF) and visceral fat (VF), as well as the ratios IF-to-VF and IF-to-SC, were calculated on a single MRI slice at the L2 level using the open-source software ImageJ. Blood samples for iron, ferritin, transferrin, and transferrin saturation (SatTr) were collected ≤1 month prior to the scan.

**Results:** The study included 51 patients with a mean age 54.3±19.6 years. A genetic cause was identified in 11 patients (21.6%). The percentage of IF showed a positive correlation with R2* (whole liver p=0.18, ROI p=0.27) and IDW (whole liver p=0.18, ROI p=0.17). Positive correlations were also observed for the IF-to-VF ratio with R2* (whole liver p=0.11, ROI p=0.04) and IDW (whole liver p=0.11, ROI p=0.04). Transferrin and SatTr showed positive correlations with TF (p=0.03–0.18), SF (p=0.04–0.31), and VF (p=0.15–0.22). No differences were observed between hereditary and acquired hemochromatosis.

**Conclusion:** We revealed a significant link between LIC and body fat composition, opening the door to innovative fat-targeted therapeutic approaches.

### SS 12.9

### Liver imaging characteristics in patients with alpha-1 antitrypsin deficiency

M. Milazzo[1], P. Roger[2], R. Sartoris[2], V. Bunel-Gourdy[3], H. Mal[3], P.-E. Rautou[2], A. Payancé[2], M. Ronot[2]; *[1]Palermo/IT, [2]Clichy/FR, [3]Paris/FR*

**Purpose:** To describe the liver imaging characteristics of patients with alpha-1 antitrypsin (AT) deficiency.

**Material and Methods:** This a retrospective analysis of patients with AT deficiency (1998–2023). Available CT, MR, and US examinations were reviewed by two radiologists to describe liver morphological, parenchymal and vascular features. A liver stiffness ≥12 kPa on vibration-controlled transient elastography (VCTE) suggested advanced chronic liver disease (cACLD). Patients with homozygous and heterogeneous Pi*Z mutations were compared with Chi-2 and Mann–Whitney U tests.

**Results:** Eighty-eight patients were included (median age 51 (44–59) years, 54 men (61%), 33 (38%) with AT substitution). CT, MRI, US, 2D shear-wave elastography (2D-SWE), and VCTE were available in 84 (95%), 60 (68%), 86 (98%), 77 (89%), and 86 (98%) patients. Lung anomalies were more frequent in patients with homozygous (88%) than heterogeneous (58%) Pi*Z mutations. Six patients (7%) had cACLD on VCTE, without association with the genetic status or lung anomalies. On imaging, any liver morphological anomalies were observed in 55% of the patients, mostly atrophy of the posterior sector (18%), segment 4 atrophy (32%), and segment I hypertrophy (40%). However, most patients had smooth liver contours (83%) and normal portal vessels (95%). Portosystemic shunts were depicted in 21% and splenomegaly in 23%. No difference in imaging characteristics were observed between the patients with homozygous or heterogeneous Pi*Z mutations.

**Conclusion:** Advanced chronic liver disease was rare in patients with AT deficiency based on VCTE. However, morphological anomalies and features of portal hypertension were observed in 20–40%. They were mild and did not seem to be associated with the genetic status.

### SS 12.10

### Fontan-associated liver disease: Liver stiffness and MR T2 mapping related to 4D flow of Fontan circulation

C. de Lange, H. Hebelka, B.-M. Ekman Joelsson, J. Sunnegårdh, S. Gustafsson, M. Johansson Synnergren, P. Svensson, H. Wåhlander, K. Lagerstrand, F. Dangardt; *Gothenburg/SE*

**Purpose:** Patients with Fontan circulation have increased central venous pressure and lymphatic obstruction, leading to Fontan-associated liver disease. The aim was to investigate if MR with four-dimensional (4D) flow, elastography (MRE), MR relaxometry (T1 and T2 mapping) and US shear-wave elastography (SWE) and shear-wave dispersion (SWD), i.e. viscosity, can assess liver fibrosis and congestion.

**Material and Methods:** Eighteen pediatric patients with Fontan circulation, admitted for annual cardiac follow-up, were prospectively included. MR including the liver, cardiac function, 4D flow, relaxometry and MRE, and hepatic SWE and SWD were performed, and findings were correlated.

**Results:** Sixteen patients, median age 13 years (range 3–18), where 11 were boys, underwent both 3T MR and US examination. Liver stiffness SWE 17.2±8.8 kPa and viscosity SWD 16.1±3.7 (m/s)/kHz were increased, as well as MRE 4.5±0.9 kPa and MR relaxation times, and T1 911±98 ms and T2 48.4±7.6 ms, as compared to previously reported adult and pediatric normal values. MR liver T2 relaxation time was found to correlate with flow in the Fontan tunnel 2.1±1.1L/min, pulmonary arteries 2.7±0.7L/min, aorta 3.8±0.3L/min and to age (R=0.6–0.7, p=0.004–0.03). SWE correlated to SWD and MRE (R=0.6–0.7, p< 0.008), but none of them correlated to Fontan circuit flows, relaxometry or cardiac function (R=0.06–0.4, p=0.4–1.0).

**Conclusion:** This small cohort study revealed increased liver stiffness, viscosity and relaxometry values in children with Fontan circulation. The T2 relaxation time, a marker of tissue oedema, correlated to flow in the Fontan circuit, while markers of elasticity and viscosity (oedema) did not. MR T2 relaxation time seems to be a promising indicator of hepatic congestion.

**11:00 - 12:30** **Room J1**

*Scientific Session SS 13*

### Advances in luminal imaging

### SS 13.1

### What is the optimal strategy for initial staging of esophageal cancer? Comparison of standard staging procedures (EUS, CT and fluorodeoxyglucose positron emission tomography/CT) and MRI

N. Guignard, V. Levy, M. Jreige, L. Haefliger, A. Wagner, C. Du Pasquier, S. Mantziari, M. Schafer, N. Vietti-Violi, C. Dromain; *Lausanne/CH*

**Purpose:** To compare diagnostic performance of MRI, CT, endoscopic US (EUS), positron emission tomography (PET)/CT and different combinations for initial staging of esophageal cancer.
**Material and Methods:** In this prospective study, we included 60 patients between 10.2017 and 12.2021 (M/F 50/10, mean age 66 ± 9 y.o.) with newly diagnosed esophageal cancer. Each patient underwent a 3T MRI in addition to the standard staging procedures (EUS, CT and fluorodeoxyglucose positron emission tomography (FDG-PET)/CT) for initial staging. Two independent readers (except for EUS) were asked to determine T-stage using MRI, CT and EUS, N-stage using MRI, CT, PET/CT and EUS, and M-stage using MRI, CT and PET/CT. Consensus was obtained in case of discordance using a third reader. The reference standard was the histopathology of the surgical specimen or TNM-staging established during tumor board meeting. Diagnostic performance of procedures was analyzed separately and in combination.
**Results:** For T-stage, area under the curve (AUC) for classifying tumors (T1–T3) versus (T4) was 0.83 for MRI, 0.80 for EUS and 0.74 for CT. For N-stage, AUC for classifying N0 versus N+ was 0.78 for MRI, 0.78 for EUS, 0.75 for CT and 0.84 for PET/CT. For M-stage, AUC for classifying M0 versus M+ was 1 for MRI and PET/CT and 0.7 for CT. AUC to differentiate T1–2N0M0 from T3–4N1–3M0–1 was 0.92 for MRI+PET/CT and 0.78 for EUS+CT+PET/CT (p-value 0.357).
**Conclusion:** MRI is highly accurate for the initial TNM staging enabling the optimal assessment of adjacent organ invasion. PET/CT and MRI are optimal combination to separate curative versus palliative patients.

### SS 13.2

### Predicting anastomotic leakage following esophageal cancer resection with the use of multimodal machine learning

M. Klontzas[1], M. Ri[2], E. Koltsakis[2], E. Stenqvist[2], G. Kalarakis[2], E. Boström[2], A. Kechagias[3], I. Rouvelas[2], A. Tzortzakakis[2]; *[1]Heraklion/GR, [2]Stockholm/SE, [3]Hämeenlinna/FI*

**Purpose:** Surgery in combination with chemo/radiotherapy is the standard treatment for locally advanced esophageal cancer. Even after the introduction of minimal invasive techniques, esophagectomy carries significant morbidity and mortality. One of the most common and feared complications of esophagectomy is anastomotic leakage (AL). The aim of our work was to develop a multimodal machine learning model combining CT-derived and clinical data, for the prediction of AL following esophagectomy for esophageal cancer.
**Material and Methods:** 471 patients were prospectively included (Jan 2010–Dec 2022). Preoperative CT was used to evaluate coeliac trunk stenosis and vessel calcification. Variables including demographics, disease stage, operation details, tumour histology, postoperative C-reactive protein (CRP) and stage were combined with CT data to build a model for AL prediction. Data were split into 80:20% for training:testing, and a XGBoost model was developed with 10-fold cross-validation and early stopping. Receiver operating characteristic (ROC) curves and respective areas under the curve (AUC), sensitivity, specificity, positive predictive value (PPV), negative predictive value (NPV) and F1-scores were calculated.
**Results:** 117 patients (24.8%) exhibited post-operative AL. The XGboost model achieved an AUC of 79.2% (95% CI 69%–89.4%) with specificity of 77.46%, sensitivity of 65.22%, PPV of 48.39%, NPV of 87.3% and F1-score of 56%. SHapley Additive exPlanation (SHAP) analysis showed the effect of individual variables on the result of the model. Decision curve analysis showed that the model was particularly beneficial for threshold probabilities between 15% and 48%.
**Conclusion:** In conclusion, a clinically relevant multimodal model can predict AL, being especially valuable in cases with otherwise low clinical AL probability.

### SS 13.3

### Prevalence of CT-detected extramural vascular invasion in gastric adenocarcinoma and its correlation with other known prognostic factors

A. Chandramohan, H.V. Reddy, D. Masih, A. Singh, I. Samarasam; *Vellore/IN*

**Purpose:** To study the prevalence of extramural vascular invasion (ct-EMVI) in gastric adenocarcinoma (GA) and its association with other known prognostic factors.
**Material and Methods:** This is an IRB-approved retrospective study of patients with GA who underwent staging CT between January 2021 and December 2022. Two experienced radiologists reviewed the staging CT for ct-EMVI and its grade, tumor location, thickness, perigastric nodes, and metastases. Grade 3 and 4 EMVI on CT were reported as ct-EMVI positive. The restaging CT was reviewed for y-ct-EMVI and y-ct-TNM stages for those who underwent neoadjuvant chemotherapy. Peritoneal fluid cytology, staging laparoscopy, and surgical histopathology findings were documented. We studied the association between ct-EMVI and other imaging findings on staging and restaging CT and surgical histopathology findings.
**Results:** 191 patients (140 males, 51 females) with a mean age of 53 ± 9 (range 23 to 93) years were included. 82.2% had poorly differentiated GA. The majority (95.9%) had T3 (n=34) and T4 (n=118) disease on baseline CT. The prevalence of ct-EMVI on staging CT was 65% (n=124) with 34% and 86% of T3 and T4 stage GA being ct-EMVI positive, respectively. There was a significant association between ct-EMVI and ct-T, N, and M stages, tumor thickness and extent, surrounding organ infiltration, peritoneal metastases, and response to neoadjuvant chemotherapy ($p<0.05$).
**Conclusion:** ct-EMVI is common (65%) among patients with advanced GA and was significantly associated with TNM stage, peritoneal metastases, and response to neoadjuvant chemotherapy.

### SS 13.4

### Association of chronic statin use, myopenia, myosteatosis and major morbidity in surgical patients with upper GI cancer

P. Franco, C. Maino, T. Giandola, C. Talei Franzesi, D. Gandola, M. Cereda, L. Gianotti, D. Ippolito; *Monza/IT*

**Purpose:** Derangements of body composition affect surgical outcomes. Chronic statin use may induce muscle wasting and impair muscle tissue quality. The aim of this study was to evaluate the association of chronic statin use, skeletal muscle area (SMA), myosteatosis and major postoperative morbidity.
**Material and Methods:** Between 2011 and 2021, patients undergoing pancreatoduodenectomy or total gastrectomy for cancer, and using statins since at least 1 year, were retrospective studied. SMA and myosteatosis were measured at CT scan. The cutoffs for SMA and myosteatosis were determined using receiver operating characteristic (ROC) curve and considering severe complications as the binary outcome. The presence of myopenia was defined when SMA was lower than the cutoff. A multivariable logistic regression was applied to assess the association between several factors and severe complications.
**Results:** After a matching procedure (1:1) for key baseline risk factors [American Society of Anaesthesiologists (ASA), age, Charlson comorbidity index, tumor site, and intraoperative blood loss], a final sample of 104 patients, of which 52 treated and 52 not treated with statins, was obtained. The median age was 75 years, with an ASA score ≥ 3 in 63% of the cases. SMA (OR 5.119, 95% CI 1.053–24.865) and myosteatosis (OR 4.234, 95% CI 1.511–11.866) below the cutoff values were significantly associated with major morbidity. Statin use was predictive of major complication only in patients with preoperative myopenia (OR 5.449, 95% CI 1.054–28.158).
**Conclusion:** Myopenia and myosteatosis were independently associated with an increased risk of severe complications. Statin use was associated with a higher risk of having major morbidity only in the subgroup of patients with myopenia.

### *SS 13.5*
### CTC reader index: Interpretation evaluation tool for performance in clinical practice
C. Muthoo, H. Goh, M. Tse, C. Roe, P. Melling, H. Lambie, D. Tolan*; Leeds/UK*

**Purpose:** British Society of Gastrointestinal Radiology (BSGAR) standards of practice and minimum dataset indicate a minimum 13% polyp identification rate (PIR) for polyps ≥ 6 mm and a positive predictive value (PPV) of >80% for CTC. We propose a CTC reader index (CTC-RI) as a novel reporter performance quantifier. CTC-RI produces a unifying value to communicate the "True Positive Polyp Identification Rate." A single estimate of "True Positive" outcomes from CTC may facilitate practical comparison between radiologists and institutions.
**Material and Methods:** 1132 CTCs performed in July–December of 2022 were retrospectively analysed against the BSGAR CTC reporting standards and published minimum data set. Data collected included: patient demographic, C-codes, E-codes and endoscopy conversion rate and findings. PIR, PPV and CTC-RI were calculated for 14 readers (13 radiologists, 1 radiographer) using the formula CTC-RI = PIR x PPV.
**Results:** The BSGAR standards derived minimum CTC-RI = 10.4 (13 x 0.8) and aspirational CTC-RI = 14.4 (16 x 0.90). 13/14 readers met or exceeded the targets set by BSGAR for PIR (range 13%–34%). However, 4/14 (29%) were below the minimum for PPV (mean =0.88, range 0.67–1). When ranking reader performance by PID or PPV alone, ranked position in the cohort varied substantially between the variables. The aspirational derived standard was exceeded by all the readers, mean CTC-RI = 21 (range 16–34), but substantial variation exists between the highest and lowest performing readers.
**Conclusion:** CTC-RI offers a simple method to compare reader performance. CTC-RI can form a basis for professional development and training in high performing services where variation exists between the readers.

### *SS 13.6*
### British Society of Gastrointestinal Radiology CTC standards in symptomatic patients: Achievable or exceedable?
C. Muthoo[1], H. Goh[1], M. Tse[2], C. Roe[1], P. Melling[1], H. Lambie[1], D. Tolan[1]*; [1]Leeds/UK, [2]Edinburgh/UK*

**Purpose:** CTC is well tolerated and applied to UK colorectal cancer diagnosis pathways. However, variability exists in imaging techniques and protocols across the UK. British Society of Gastrointestinal Radiology (BSGAR) published standards of practice and a minimum dataset to help standardisation, including minimum targets (BMT) and aspirational targets (BAT). We evaluated current CTC service performance in a large UK Teaching hospital serving a population of 800,000.
**Material and Methods:** CTCs performed in July–December 2022 were retrospectively analysed using the BSGAR CTC reporting standards and published minimum data set. CTC proforma reports and relevant endoscopy and pathology records were extracted from an electronic health record system (PPM+, Leeds) including patient demographic, summary C-codes, E-codes and conversion rates for subsequent endoscopic investigations.
**Results:** 1,132 CTCs were analysed by 14 reporters. 100% of the reporters were on track to exceed the BMT of 100 CTC reports per annum (5 exceeding BAT of 175). 23% (265/1132) were graded as C2 or above (excluding C3cs—indeterminate strictures) for demonstrating a polyp ≥ 6mm (BAT > 16%). Regardless of C-code, 167/1132 (15%) had subsequent endoscopy (target < 25%). 114/167 endoscopies were performed for polyps ≥6mm with a PPV of 88% (100/114, BMT > 80%). However, 151 scans reporting a C-code of ≥ C2 (excluding C3c) had no follow-up endoscopy.
**Conclusion:** BSGAR CTC standards are achievable and act as an excellent benchmark for audit in clinical practice. However, further evaluation is required to understand the reasons positive colonic findings at CTC do not progress to colonoscopy.

### *SS 13.7*
### Diagnostic performance of CT for locally advanced colon cancer and the role of extramural venous invasion and tumour deposits: A re-evaluation study
J. Shkurti[1], M. Pijl[2], R. Beets-Tan[1], J. Nederend[3]*; [1]Amsterdam/NL, [2]Arnhem/NL, [3]Eindhoven/NL*

**Purpose:** To assess the improvement of diagnostic accuracy of CT for colon cancer staging through re-evaluation, emphasizing novel imaging biomarkers such as extramural venous invasion (EMVI) and tumour deposits.
**Material and Methods:** Retrospective analysis was conducted on CT scans of colon cancer patients treated between 2011 and 2020 at two major hospitals in the Netherlands. Two radiologists independently re-evaluated the scans using a structured scoring template. Combined sensitivity and specificity analyses with 'OR rule' and 'AND rule' (e.g. Weinstein et al., 2005) were applied for dual observer evaluation, focusing on pathological stages (p)T3–4, lymph node metastasis (pN+), EMVI, and tumour deposits.
**Results:** Of 1189 patients, initial CT evaluations showed sensitivity and specificity (95% CI) of 0.75 (0.72–0.78) and 0.58 (0.50–0.66) for predicting pT3–4, and 0.40 (0.31–0.50) and 0.74 (0.67–0.81) for pN+, respectively. Upon re-evaluation, the combined sensitivity and specificity with 'OR rule' were 0.94 and 0.33 for pT3–4, and 0.89 and 0.34 for pN+; and with 'AND rule' 0.55 and 0.82 for pT3–4, and 0.43 and 0.98 for pN+. EMVI was identified in 42% (497/1189) of the cases by at least one observer and in 15% (182/1189) by both. Tumour deposits were reported in 14% (166/1189) of the cases by at least one observer and in 2% (28/1189) by both.
**Conclusion:** Double reading refines diagnostic accuracy by enhancing sensitivity with the 'OR rule' and improving specificity with the 'AND rule'. Implementing a scoring template can potentially improve accuracy, especially for EMVI and tumour deposits. Nonetheless, the low inter-observer agreement in detecting these biomarkers indicates a need for improved standardization.

### *SS 13.8*
### Radiomics in colon cancer: How to identify high-risk patients?
M. Polici, M. Zerunian, D. Valanzuolo, D. Pugliese, D. De Santis, D. Caruso, A. Laghi*; Rome/IT*

**Purpose:** The study was aimed to develop a radiomic model able to identify high-risk colon cancer by analyzing pre-operative CT scans.
**Material and Methods:** The study population included 300 patients with non-metastatic colon cancer, retrospectively enrolled from January 2015 to June 2020. Population was divided into two groups, progressive and non-progressive. All baseline CT scans were used for radiological staging and 3D cancer segmentation, the latter performed by two expert radiologists using open-source software (3DSlicer v4.10.2). Among the 107 radiomic features extracted, stable features were selected to evaluate the inter-class correlation (ICC) (cutoff ICC > 0.8). Stable features were compared between the two groups (T-test or Mann–Whitney), and the significant features were selected for univariate and multivariate logistic regression to build predictive models (radiomic, clinical and combined).
**Results:** In total, 253/300 were progressive and 47/300 non-progressive. 33 radiomic features were stable (0.80≤ICC<0.92) and 23 features significantly different between the two groups (p<0.05). From the univariate analysis, 5 radiomic features resulted to be correlated with the progression. Among clinical and radiological data, sex, cT, cN, and extramural venous invasion (EMVI) status yielded a significant correlation with the progression (OR≠1 and P<0.05). The combined model (radiomic plus clinico-radiological parameters) reached the best results with an area under the curve (AUC)=0.75 (P=0.012), followed by the clinical model with AUC=0.69 (P=0.009), while the radiomic model alone had no significant results (P>0.05).
**Conclusion:** In conclusion, the combined model outperformed both clinical and radiomic models, reinforcing the idea that quantitative imaging could be integrated with the conventional clinico-radiological evaluations to support the oncologists in identifying the patients with high-risk of progressive disease.

### *SS 13.10*

**Evidence for a combined T3–4 CT staging group as a biomarker for using neoadjuvant chemotherapy in locally advanced colon cancer: A FOxTROT trial analysis**

J.R. Platt, F. Elliott, J. Seligmann, N. West, D. Tolan*; Leeds/UK*

**Purpose:** To assess CT staging accuracy in the multi-centre FOxTROT trial, where neoadjuvant chemotherapy (NAC) reduced recurrence risk over upfront surgery in locally advanced (cT3–4 cN0–2) but resectable colon cancer.

**Material and Methods:** In this pre-planned, prospective analysis, the accuracy of CT for predicting T stage, N status (N0 vs. N+) and extramural venous invasion (EMVI) status was assessed by comparing CT and pathological staging in the straight-to-surgery group. The impact of mismatch repair (MMR) status and tumour side were explored.

**Results:** Of 354 participants, T stage agreement was 63.0%, with 78.9% and 41.1% of T3 and T4 tumours correctly predicted, respectively. The positive predictive value (PPV) of CT for identifying T3–4 tumours was 94.5% and for T3 (>5 mm)–4 tumours was 64.4%. The overall agreement, sensitivity, specificity, PPV and negative predictive value for predicting N status were 54.1%, 81.1%, 26.0%, 53.2% and 57.1%, respectively. For EMVI, these values were 54.9%, 71.0%, 41.2%, 50.7% and 62.5%, respectively. A lower PPV for predicting N status was seen in MMR-deficient, versus -proficient, tumours (46.3% vs. 56.4%). Lower T stage agreement and specificity for predicting N status were seen in right-sided, versus left-sided, tumours (59.5% vs. 67.1% and 17.6% vs. 34.1%, respectively). However, these subgroup differences were not statistically significant.

**Conclusion:** In FOxTROT, CT accurately identified T3–4 tumours, with very few T1–2 tumours incorrectly over-staged. We propose that a combined T3–4 CT staging group is an effective biomarker for using NAC to treat locally advanced colon cancer in routine practice. The impact of MMR status and tumour side warrant prospective evaluation.

**11:00 - 12:30** **Room J2**

*Scientific Session SS 14*

### Acute abdomen

### *SS 14.1*

**CT description of abdominal complications due to fishbone ingestion**

A. Ben Zitoun, A. Aboikoni, M. Zappa, L. Garzelli*; Cayenne/GF*

**Purpose:** Inadvertent ingestion of fishbone can lead to bowel perforation. Numerous but only case reports have been published, and therefore, we aimed to describe the localization and type of complication in a dedicated cohort.

**Material and Methods:** From 2009 to 2023, we query our local informatics department (Cayenne Hospital, French Guiana) to search through electronic patient records (ICD-10 code T18: Foreign body in alimentary tract), and we reviewed each case to identify abdominal complication caused by fishbone ingestion. Patients with complication occurring above the stomach and without a available CT were excluded. Clinical, biological, imaging and treatment-related data were retrospectively collected. The overall raw incidence was estimated.

**Results:** Forty-two patients (mean age: 59.5 years (±15.6); female 35.7%) were included for analysis between May 2009 and June 2023. Abdominal pain was present in 41/42 patients (98%) and inflammatory biological syndrome was found in 34/42 patients (81%). On CT, perforation of the bowel wall was observed in 41/42 patients (98%), intra-abdominal collection in 17/42 patients (40%) and pneumoperitoneum in 2/45 patients (5%). Most common sites of complication were the ileum 11/42 (26%), stomach 8/42 (19%) and the right colon 5/42 (11%). Regarding treatment, surgery was performed in 23/42 patients (55%), conservative treatment by antibiotics was administrated in 14/42 (33%) and endoscopic removal was possible in 4/42 (10%). The estimated incidence was 2.9 per 100.000 person-years.

**Conclusion:** We reported the largest analysis of bowel perforation secondary to fishbone ingestion providing an exhaustive spectrum of localization and type.

### *SS 14.2*

**CT evaluation of GI system perforations: A retrospective comparative analysis between retroperitoneal and intraperitoneal perforation sites**

U.E. Karabulut, H. Toprak, Y. Polat, Z. Donmez, A. Akcay, M. Gultekin, T. Yılmaz*; Istanbul/TR*

**Purpose:** The objective of this study was to retrospectively evaluate the distinctive MDCT findings of intraperitoneal and retro/extraperitoneal perforations by comparing the locations of free air in the abdomen and other predictive imaging findings. The aim was to contribute to the preoperative localization of surgically confirmed GI tract perforations. Correct identification of the etiology and precise anatomical location of the perforation hold paramount significance for ensuring optimal therapeutic interventions and surgical planning.

**Material and Methods:** 226 patients with acute abdominal pain who visited the emergency department and underwent contrast-enhanced CT scans between January 2016 and November 2023 were included in the study. There were 146 male and 80 female patients with a mean age of 53.1 years. The site of perforation was based on surgery in all the cases. Two radiologists assessed the presence of specific air distributions and the strong predictors of GI system (GIS) perforation in a consensus blind to operative findings.

**Results:** 192 intraperitoneal and 34 retro/extraperitoneal perforation cases were included in the study. Among specific air distributions, subphrenic free air was statistically significant in differentiating intraperitoneal GI tract perforation ($p<0.05$), whereas free air in the minor pelvis, right lower quadrant free air, left lower quadrant free air, and retroperitoneal free air were statistically significant in differentiation of retro/extraperitoneal GI tract perforation ($p<0.05$). Among strong imaging predictors, only ascites were statistically significant in the differentiation of intraperitoneal perforations from retro/extraperitoneal perforations ($p<0.05$).

**Conclusion:** MDCT findings may serve as predictive indicators for the precise localization of GIS perforations which is crucial for appropriate management and surgical planning.

Scientific Sessions

Authors' Index

### SS 14.3

### The accuracy of color Doppler US indices for diagnosis of acute cholecystitis

F. Salahshour, M. Behzadi, A. Abkhoo, N. Mohammadzadeh, J. Zebardast, A. Ghasemlouei, N. Ayoobi Yazdi; *Tehran/IR*

**Purpose:** US diagnosis of acute cholecystitis may be challenging with a significant impact on patient management. We aimed to evaluate the value of Doppler investigation of the hepatic and cystic arteries to diagnose acute cholecystitis.
**Material and Methods:** Gray-scale and Doppler US of 78 patients scheduled for cholecystectomy was made within two days of surgery. The gray-scale variables and Doppler indices including peak systolic velocity (PSV) and resistive index (RI) of hepatic and cystic arteries were evaluated and correlated with histopathology findings as the gold standard.
**Results:** The study sample constitutes 25 acute and 53 chronic cholecystitis based on histopathology reports. Among Doppler US indices, cystic artery PSV with the cutoff value of 28.5 cm/s (sensitivity: 84%, specificity: 73.5%, area under the curve (AUC): 0.758, p<0.001; CI 0.729–0.937) and the hepatic artery PSV at a cutoff of 60 cm/s (sensitivity: 80%, specificity: 64.2%, AUC: 0.833, p<0.001; CI: 0.640–0.786) had high accuracy in diagnosing acute cholecystitis. RI of the cystic artery (AUC: 0.305) or hepatic artery (AUC: 0.328) did not show acceptable accuracy. It is worth noting that adding hepatic artery PSV with a cutoff value of 58 cm/s to the gray-scale variables will increase the sensitivity from 50.2% to 98% and specificity from 98% to 100% (p<0.001), resulting in a significant rise in the accuracy of diagnosing acute cholecystitis.
**Conclusion:** PSV of cystic artery or PSV of hepatic artery showed statistically significant accuracy in the diagnosis of cholecystitis and has the potential to improve the precision of acute cholecystitis.

### SS 14.4

### Qualitative and quantitative value of virtual monochromatic versus polychromatic conventional CT images for detecting acute bowel ischemia

K. Rothenbühler, A. Sayadi, R. Troxler, C. Sempoux, D. Rotzinger, S. Schmidt; *Lausanne/CH*

**Purpose:** To compare 40keV virtual monochromatic images (VMIs) with conventionally acquired, polychromatic CT images to detect acute bowel ischemia (ABI).
**Material and Methods:** We retrospectively included 47 patients with pathologically proven ABI, consecutively admitted in emergency between January 2018 and April 2021. They underwent contrast-enhanced abdominal CT followed by surgery within <48h. Eighteen CT examinations were acquired in dual-energy mode (VMI) and 29 in conventional polychromatic mode (CONV). After dividing the small and large bowels into 10 segments, 3 radiologists read CONV and VMI (virtual unenhanced and 40keV VMIs in venous phase) examinations separately and blinded to the exact localization of ABI. Second, two radiologists quantitatively analysed bowel wall density and venous enhancement on VMI and CONV measuring mean attenuation and standard variation of each bowel segment separately. Interobserver agreement and differences in the detection of ABI between the two patients' groups were determined, considering p <0.05 as significant.
**Results:** Qualitative analysis showed good sensitivity (72–86%) and specificity (82–89%) for detecting ABI per bowel segment for all readers on CONV and VMI without significant differences between both the techniques and important or near-perfect inter-observer agreement calculated per bowel segment (kappa 0.61–0.83). Nearly, all quantitative results were statistically significant comparing ill versus healthy segments, both on (virtually) non-enhanced and enhanced acquisitions, without significant difference between VMI and CONV.
**Conclusion:** Our comparison of VMI with CONV images for detecting ABI revealed good diagnostic value and substantial or near-perfect inter-reader agreement among all radiologists for both techniques, without significant qualitative or quantitative differences.

### SS 14.5

### Acute arterial mesenteric ischaemia: Comparison of occlusion and stenosis of the superior mesenteric artery

R. Dufay[1], L. Garzelli[1], A. Tual[1], I. Ben Abdallah[2], O. Corcos[1], A. Nuzzo[1], V. Vilgrain[1], M. Ronot[1]; *[1]Clichy/FR, [2]Paris/FR*

**Purpose:** Stenotic arterial acute mesenteric ischaemia (AMI) has not been properly described. Our aim was to determine whether stenosis of the superior mesenteric artery (SMA) is associated with a different presentation and outcome than complete occlusion of the SMA in arterial AMI.
**Material and Methods:** Clinical characteristics at admission, CT, treatments, and outcomes were compared between the patients with complete (occlusive) or incomplete (stenotic) SMA obstruction after adjusting for aetiology (embolic or atherosclerotic). Primary outcome was 30-day mortality, and secondary outcome was 6-month GI disability-free survival (no short bowel syndrome or parenteral nutritional support or permanent stoma).
**Results:** One hundred and fifty-one patients (65 women, mean age 69 years) were included, 62 (41%) with stenotic AMI and 89 (59%) with occlusive AMI. Sixty-four (42%) and 87 (68%) had, respectively, an embolic or an atherosclerotic aetiology. After adjusting for aetiology, the presence of chronic kidney failure (p=.03) and normal bowel enhancement on CT (p<.01) were associated with stenotic AMI. Stenotic AMI patients were more frequently treated by endovascular revascularization (p<.01) and stenting (p<.01), and occlusive AMI patients by open revascularization (p<.01). The 30-day mortality rate was 20/151 (13%) with no difference between stenotic (7/62, 11%) and occlusive (13/89, 15%; p=.89) AMIs. However, occlusive AMI patients had a lower rate of 6-month GI disability-free survival (66% (41/62) stenotic AMI versus 44% (39/89) for occlusive AMI; p=.01).
**Conclusion:** SMA stenosis can cause AMI with a 30-day mortality rate that is similar to that in occlusive forms. However, complete occlusion of the SMA is associated with poorer GI outcomes.

### SS 14.6

### Predicting the need for resection and the occurrence of irreversible transmural intestinal necrosis in mesenteric venous thrombosis: Prospective study from an intestinal stroke center

F. Matteini[1], A.-P. Vergeau[2], R. Cannella[1], A. Nuzzo[2], O. Corcos[2], D. Cazals-Hatem[2], V. Vilgrain[3], G. Brancatelli[1], M. Ronot[3]; *[1]Palermo/IT, [2]Paris/FR, [3]Clichy/FR*

**Purpose:** The prognosis of acute mesenteric ischemia (AMI) caused by mesenteric venous thrombosis (MVT) remains undetermined. Predicting the need for resection and the occurrence of irreversible transmural intestinal necrosis (ITIN) is challenging. The aim of this study was to identify factors associated with the need for bowel resection rate and with ITIN in patients with MVT.
**Material and Methods:** This is a single-center prospective cohort study including consecutive patients with MVT admitted to the intestinal vascular emergency unit (January 2016 to January 2023). Admission and pre-operative CT were reviewed by two radiologists. The co-primary outcome was the need for resection and the occurrence of ITIN. ITIN was pathologically confirmed after resection. Patients who recovered without intestinal resection were considered free from ITIN. Independent risk factors for resection or ITIN were identified by multivariable binary logistic regression analysis.
**Results:** 94 patients were included (68% male, median age 58.0 years). The median follow-up was 21 months. Intestinal resection and ITIN concerned 34% and 26% of patients, respectively. Factors at admission associated with the need for resection were abdominal guarding (odds ratio [OR] 3.6, [95% CI 1.0–12.1], $P$=0.043), morphine-requiring pain (OR 6.0 [1.6–22.3], $P$<0.008), and bowel loop dilation (OR 6.8 [1.3–35.1], $P$=0.022). Considering preoperative CT, ITIN was associated with lactate >2mM (OR 14.4 [1.7–121.8], $P$=0.014), and bowel wall hyperattenuation on precontrast images (OR 15 [2.2–102.8], $P$=0.006).
**Conclusion:** Several predictive factors of resection and ITIN can be identified in patients with MVT. These factors may help inform treatment decision making and improve patients' outcome.

### SS 14.7

### Incidence of acute mesenteric ischaemia in French Guiana: A reflection of the situation in South America

L. Garzelli[1], G. Kenou[2], L. Tanga[3], K. Drak Alsibai[1], M. Zappa[1], M. Nacher[1]; [1]Cayenne/GF, [2]Saint-Laurent-du-Maroni/GF, [3]Kourou/GF

**Purpose:** Epidemiologic studies regarding acute mesenteric ischaemia (AMI) in South America are missing. We aimed to evaluate the incidence of AMI in a well-defined region-wide population-based study in French Guiana.
**Material and Methods:** This is a retrospective multicentre study that included all AMI patients admitted to one of the three hospitals in French Guiana (Cayenne, Kourou, West-Guiana) between January 2013 and December 2022. Patients were identified from electronic patient records (ICD-10 code K55.0: Vascular disorders of intestine), and radiological and pathology reports (necrotic bowel sample). All identified patients CT scans were then carefully reviewed to confirm or exclude the diagnosis. Inclusion criteria were having arterial occlusive, venous, or non-occlusive AMI. Exclusion criteria were iatrogenic or traumatic causes, intestinal ischaemia following bowel obstruction and ischaemic colitis. The raw incidence and the age-adjusted incidence were calculated, and the 30-day mortality was measured.
**Results:** From 274 patients yielded, 22 patients were true AMI cases and were included in the present analysis (sex ratio=1; mean age of 64 years). The population living in French Guiana averaged 278000 patients during the study period. The overall crude incidence rate was 0.79 per 100.000 person-years (95% CI 0.52–1.2) and the age-standardized rate (European standard population) was 1.92 per 100.000 person-years (1.14–2.97). AMIs were arterial occlusive in 13/22 (59%) patients, venous in 7/22 (32%) and non-occlusive in 2/22 (9%) patients. The 30-day mortality was 11/22 (50%).
**Conclusion:** The incidence of AMI in French Guiana is lower than reported in Northern Europe. Whether our estimates are representative of other South American countries requires further research.

### SS 14.8

### Inter-reader reliability for the assessment of portal vein thrombosis

V. Plaforet[1], L. Moga[1], L. Elkrief[2], P.-E. Rautou[1], M. Ronot[1]; [1]Clichy/FR, [2]Tours/FR

**Purpose:** To assess the inter-reader reliability for the assessment of portal vein thrombosis (PVT).
**Material and Methods:** Two abdominal radiologists independently reviewed 44 CTs from two centers (34 patients with cirrhosis and 10 with porto-sinusoidal vascular disorder). CTs were selected to represent a spectrum of PVT scenarios. Measurements were performed on a plane strictly perpendicular to the main portal vein (MPV) and included the largest diameter of the MPV (mm), the largest thickness of the thrombus (mm), the smallest remnant lumen (RL) (mm), the surface of the MPV ($mm^2$), the thrombus surface ($mm^2$) and the lumen surface ($mm^2$). The remnant lumen as percentage of the MPV diameter (%RLdiam) or surface (%RLsurf) was computed and considered as the primary criteria. Inter-reader reliability was assessed using the Cohen kappa, intraclass correlation coefficient (ICC) and Bland–Altman plots.
**Results:** ICCs were 0.93 (95% CI 0.87–0.96) and 0.83 (95% CI 0.69–0.91) for the %RLdiam and %RLsurf, respectively. On the Bland–Altman plots, the %RLdiam showed no bias, and the limits of agreement (LoA) were +/– 22%. The %RLsurf has wider LoA (– 32% to +40%) with a bias of 4%. We further categorized the %RLdiam into 3 categories (<50%, >50% and 100%) or 4 categories (<25%, 25–50%, 50–75% and >75%). The inter-reader reliability was higher with 3 categories (kappa 0.65+/– 0.10; 35/44 (80%) agreement) than 4 (kappa 0.58+/– 0.10; 32/44 (73%) agreement).
**Conclusion:** The % of the remnant PV lumen diameter should be used as criteria to assess the extent of PVT, which can be further described using 3 categories. A significant increase (decrease) corresponds to ≥25% (≤25%) change.

### SS 14.9

### CT for evaluation of abdominal wall hernias: What is the value of the Valsalva maneuver?

S. Stocker, A. T. Hoppe, M. Lange, A. Tognella, M. Bueter, K. Lehmann, H. Alkadhi, D. Stocker; Zurich/CH

**Purpose:** To investigate the differences in the detection rate, visibility, and size of abdominal wall hernias in CT with and without Valsalva maneuver.
**Material and Methods:** This single-center retrospective study included consecutive patients who underwent abdominal CTs with Valsalva maneuver between January 2018 and January 2022. Inclusion criteria were availability of an additional non-Valsalva CT within 6 months. A combined reference standard including clinical and surgical findings was used. Two independent, blinded radiologists measured the hernia sac size and rated hernia visibility on CTs with and without Valsalva. Differences were tested with a Wilcoxon signed rank test and McNemar's test.
**Results:** The final population included 95 patients (16 women; mean age 46 ± 11.6 years) with 205 hernias. Median hernia sac size on Valsalva CT was 31 mm compared with 24 mm on non-Valsalva CT (p<0.001). In 73% and 82% of the cases, the hernias were better visible on CT with Valsalva as compared to that without. 14% and 17% of hernias were only visible on the Valsalva CT. Hernia visibility on non-Valsalva CT varied according to the subtype, with only 0% and 3% of the umbilical hernias not being visible compared with 43% of the femoral hernias that were not visible without Valsalva.
**Conclusion:** Abdominal CT with Valsalva has a higher detection rate for abdominal wall hernias than CT without Valsalva. The hernias are better visible, larger, and some hernias are only visible on the Valsalva CT. Therefore, this method should be preferred for the evaluation of abdominal wall hernias.

### A

Abkhoo A.: SS 14.3
Aboikoni A.: SS 14.1
Absil J.: SS 8.5
Ackland S.: SS 9.10
Adair A.: SS 6.9
Aghayan D.: SS 8.1
Aguilera L.: SS 8.9
Ahlström H.: SS 10.8
Ahmadova N.: SS 12.8
Ahn J.: SS 10.4
Akcay A.: SS 14.2
Akdal Dolek B.: **SS 12.6**
Akdeniz A.: SS 10.10
Akpinar G.: SS 12.3
Alderson S.: SS 9.9
Alitti C.: SS 2.6
Aljumaa A.: **SS 12.7**
Aljumaa J.: SS 12.7
Alkadhi H.: SS 14.9
Allaire M.: SS 4.5
Allouch A.: SS 11.5
Alsaqal S.: **SS 10.8**
Amaddeo G.: SS 4.5
Ambros R.: SS 7.1
Andrašina T.: SS 1.9
Arico F.: SS 3.6
Aslan S.: SS 12.4
Assuncao A.: SS 9.7
Aubé C.: SS 8.3
Auernhammer C.: SS 11.4, SS 11.6
Augustine A.: **SS 3.3**
Axelsson J.: SS 3.2
Aydin E.: SS 12.3
Ayhan S.: SS 10.9
Ayoobi Yazdi N.: SS 6.10, SS 14.3
Azmoudeh-Ardalan F.: SS 6.10

### B

Balcar L.: SS 12.5
Balcı Y.: SS 10.10
Bali M.A.: SS 8.5
Balkhed W.: SS 12.1
Ball C.: SS 3.8
Bando-Delaunay A.: SS 11.5
Baratta F.: SS 1.5, SS 7.7, SS 11.8
Bard A.: SS 5.5, SS 5.9
Bardhi E.: SS 1.7, SS 2.7, SS 2.8, **SS 7.7**, **SS 11.8**
Bariani M.: SS 8.10
Barrow B.: SS 5.5
Bárta R.: **SS 1.9**
Bartolotta T.: SS 10.2
Basaran S.: **SS 10.10**
Ba-Ssalamah A.: SS 7.1, **SS 12.5**
Bastati-Huber N.: SS 7.1, SS 12.5
Båtsman M.: SS 3.2
Beable R.: SS 3.8
Beaufrère A.: SS 4.7, SS 11.9
Beek K.: **SS 5.8**
Beer L.: SS 12.5
Beets G.: SS 3.6
Beets-Tan R.: SS 3.6, SS 11.1, SS 11.2, SS 13.7
Behzadi M.: SS 14.3
Bellamy C.: SS 6.9
Ben Abdallah I.: SS 14.5
Ben Zitoun A.: SS 14.1
Ben-Ami Shor D.: SS 1.3
Benke M.: SS 1.2
Benkert T.: SS 8.5
Bennett D.: SS 5.5
Bereska J.: **SS 1.8**, SS 8.1
Bereska L.: SS 1.8, SS 8.1
Berger F.: SS 11.4
Berzigotti A.: SS 12.2
Besselink M.: SS 1.8, SS 8.1
Besson F.: SS 4.5
Bhagwanani A.: SS 5.1, SS 5.2, SS 5.5
Bhatnagar G.: SS 5.1, **SS 5.2**, **SS 5.3**, **SS 5.9**
Bini G.: SS 6.4
Bitetto D.: SS 2.4
Blaise L.: SS 2.6
Blanc J.-F.: SS 2.6
Blandino A.: **SS 6.4**
Blasi A.: SS 2.1
Blomqvist L.: SS 3.2, SS 3.10
Bloom S.: SS 5.1
Bogaards J.: SS 7.4
Bonvalet F.: SS 8.9
Boros C.: SS 8.9
Bos D.: SS 7.6, SS 8.2
Bossuyt P.: SS 1.1
Boström E.: SS 13.2
Bouattour M.: SS 4.5
Boubaya M.: SS 4.5
Boutron I.: SS 2.3
Boxhoorn R.: SS 7.6
Brancatelli G.: SS 6.4, SS 14.6
Brännström F.: SS 3.2
Brodersen J.: SS 5.6
Bronswijk M.: SS 1.1
Budai B.: SS 1.2
Buelu S.: **SS 7.2**
Bueter M.: SS 14.9
Bunel-Gourdy V.: SS 12.9
Buskens C.: SS 5.8
Byrne C.D.: SS 10.4
Byun J.H.: SS 1.10

### C

Campinchi J.: SS 11.9
Cannella R.: SS 1.4, SS 4.7, SS 6.4, SS 10.2, SS 14.6
Capanu M.: SS 9.6
Carbone M.: SS 7.3
Carter L.: SS 5.10
Caruso D.: SS 10.5, SS 13.8
Castagnoli F.: SS 3.5
Catucci D.: **SS 12.2**
Cazals-Hatem D.: SS 14.6
Cederborg A.: SS 12.1
Cereda M.: SS 13.4
Cetinic I.: **SS 10.6**
Chai N.: SS 9.10
Chakraborty J.: SS 9.7
Chalaye J.: SS 4.5
Chandramohan A.: SS 3.3, **SS 13.3**
Chang W.: SS 3.1
Chang Y.: SS 10.4
Chaniotaki N.: SS 5.4
Charles-Edwards G.: SS 3.5
Chavoshi M.: SS 5.7
Che F.: **SS 6.7**
Chen J.: SS 4.7
Chen W.: SS 4.7
Chen Y.: SS 4.7, SS 6.2
Chernomorets A.: SS 1.3
Choi J.: SS 6.1
Choi J.H.: SS 10.4
Choi S.H.: SS 6.1
Choi S.J.: SS 1.10, **SS 6.1**
Choi W.-M.: SS 6.1
Christe A.: SS 12.2
Chung H.-J.: SS 4.2, **SS 10.3**, SS 10.4
Chung S.: SS 6.1
Cingarlini S.: SS 2.7
Clarke C.: SS 5.1, SS 5.9
Coilly A.: SS 4.5
Constantin C.: SS 1.4
Conwell D.L.: SS 8.7
Coquelet N.: SS 8.5
Corcos O.: SS 14.5, SS 14.6
Corr A.: SS 5.1
Correas J.-M.: SS 10.1
Costentin C.: SS 4.5
Cowen J.: SS 3.8
Créquit P.: SS 2.3
Crimì F.: SS 3.4, SS 3.7
Cristoferi L.: SS 7.3
Cros J.: SS 8.9, SS 11.5
Curran S.: SS 7.2

### D

D'Alessandro C.: SS 3.4, SS 3.7
D'Haens G.: SS 5.8
D'Onofrio M.: SS 1.5, SS 1.7, SS 2.7, SS 2.8, SS 7.7, SS 8.8, SS 11.8
Daggett S.M.: SS 7.8
Dahlqvist Leinhard O.: SS 8.4, SS 12.1
Dahlström N.: SS 12.1
Dal Magro C.: SS 3.4, SS 3.7
Dangardt F.: SS 12.10
Davendralingam N.: **SS 4.3**
Davì M.: SS 2.7
Davis T.: SS 7.4
De Bernardi E.: SS 7.3
de Boer Y.: SS 7.4
de Jonge C.: SS 5.8
de Lange C.: SS 10.6, **SS 12.10**
de Mestier L.: SS 8.9, SS 11.5
De Robertis R.: SS 1.5, SS 1.7, **SS 2.7**, SS 2.8, SS 7.7, SS 8.8, SS 11.8
de Rycke O.: SS 11.5
De Santis D.: SS 10.5, SS 13.8
de Voogd F.: SS 5.8
de Vries A.: SS 7.4
De Vuysere S.: SS 1.1
Demory A.: SS 2.6
Denolin V.: SS 8.5
Di Giuseppe R.: SS 2.9
Dinsey M.: SS 3.8
Dioguardi Burgio M.: SS 4.5, SS 4.7, **SS 10.1**, SS 11.9
Dohan A.: SS 4.5
Donmez Z.: SS 14.2
Doukas M.: SS 8.2, SS 10.7
Drago S.: SS 9.1
Drak Alsibai K.: SS 14.7
Driessen S.: SS 10.7
Dromain C.: SS 13.1
Du Pasquier C.: SS 13.1
Dudás I.: **SS 1.2**
Dufay R.: SS 14.5
Dwarkasing R.: SS 7.4, SS 7.6

### E

Ebeling Barbier C.: SS 10.8
Ebner L.: SS 12.2
Ebner R.K.: **SS 11.7**
Edwin B.: SS 8.1
Eid M.: SS 1.9
Ekman Joelsson B.-M.: SS 12.10
Ekstedt M.: SS 12.1
Ekvall N.: SS 10.6
El Homsi M.: **SS 9.6**, SS 9.8
Elbanna K.: SS 4.8
Elkrief L.: SS 14.8
Elliott F.: SS 13.10
Elmokattaf R.: SS 12.7
Eryuruk U.: **SS 12.4**
Esen K.: SS 10.10
Eze V.N.: SS 5.1

### F

Farasat M.: **SS 10.9**
Feng S.-T.: **SS 7.9**
Fernandes A.: SS 11.3
Fernandes De Paula Tito Jorge M.C.: SS 9.6, SS 9.8
Ferrara V.: SS 2.4
Ferreira C.: SS 7.4
Firat C.: SS 9.7
Fitzke H.: SS 5.5
Fogel E.: SS 8.7
Forsgren M.F.: **SS 8.4**, **SS 12.1**
Fortuna L.: **SS 8.10**
Fournier I.: SS 1.4
Franco P.: SS 7.3, SS 9.1, **SS 13.4**
Franklin J.: SS 2.4, SS 5.1
Franssen S.: SS 7.10
Fretland Å.: SS 8.1
Fuchsjäger M.: SS 12.8
Fuqua L.J.: SS 9.6

### G

Gagliano D.: SS 10.2
Gandola D.: SS 13.4
Ganne-Carrié N.: SS 2.6, SS 4.5
Garcia-Aguilar J.: SS 9.7
García-Criado Á.: SS 2.1
Garofano A.: SS 8.8
Garrett J.W.: SS 11.10
Garzelli L.: **SS 2.9**, **SS 14.1**, **SS 14.5**, **SS 14.7**
Gavves E. SS 1.8, SS 8.1
Gecse K.: SS 5.8
Gershon A.: SS 4.8
Ghasemlouei A.: SS 14.3
Ghavidel F.: SS 6.10
Giandola T.: SS 9.1, SS 13.4
Gianotti L.: SS 13.4
Gleeson D.: SS 7.2, SS 7.5
Goedegebuure F.: **SS 3.6**
Goh H.: SS 13.5, SS 13.6
Goldberg N.: SS 2.2
Gollub M.J.: SS 9.7
Gong X.: **SS 9.5**
Gordon Smith J.: SS 6.9
Gormly K.: **SS 9.10**
Gourtsoyianni S.: SS 5.4
Gowland P.: SS 5.9
Grando V.: SS 2.6
Greenhalgh R.: SS 5.1
Grégory J.: **SS 2.3**, **SS 11.9**
Groot Koerkamp B.: SS 7.10
Guignard N.: **SS 13.1**
Guiu B.: SS 4.5
Gullo G.: **SS 2.4**
Gultekin M.: SS 14.2
Gupta A.: SS 5.1
Gurney-Champion O.J.: SS 10.7
Gustafsson S.: SS 12.10

### H

Haefliger L.: SS 13.1
Halligan S.: SS 5.1
Hameed M.: SS 5.1, SS 5.5
Hamlin J.: SS 5.1
Hansel B.: SS 2.9
Hart A.: SS 5.1
Hebelka H.: SS 10.6, SS 12.10
Heiselman J.: SS 9.7
Hejazi J.: SS 12.7
Helbren E.: SS 5.1
Hentic O.: SS 11.5
Heverhagen J.T.: SS 12.2
Higginson A.: SS 5.1
Hilbert T.: SS 8.6
Hiroz P.: SS 1.4
Hoad C.: SS 5.9
Hobeika C.: SS 10.1
Hockings P.: SS 10.8
Hogg W.: SS 5.1
Hohenberg F.: SS 12.8
Hollande C.: SS 4.5
Holleboom A.G.: SS 10.7
Holmberg M.: SS 12.1
Holmes J.: SS 5.5
Hopkinson G.: SS 3.5
Hoppe A.T.: SS 14.9
Horsthuis K.: SS 5.8, SS 7.4
Horvat N.: **SS 9.7**, SS 9.8
Hrycyk J.: SS 12.2
Huang Q.: SS 3.9
Huber A.T.: SS 12.2
Huddy J.: SS 5.10
Huiskens J.: SS 8.1
Hur B.Y.: SS 3.1
Hwang S.: SS 1.6

### I

Ingenerf M.K.: **SS 11.4**, **SS 11.6**
Ippolito D.: SS 7.3, SS 9.1, SS 13.4

### J

Jafarian A.: SS 6.10
Jameson M.: SS 9.10
Jamil F.: SS 6.10
Jang H.-J.: SS 4.8
Jang J.K.: **SS 1.10**
Janssen B.: SS 8.1
Jennings K.: SS 8.7
Jensen M.D.: SS 5.6
Jenssen H.: SS 8.1
Jia X.: **SS 6.6**
Jiang H.: **SS 4.7**, SS 6.2, SS 6.5, SS 6.6
Jinnah N.: SS 5.9
Johansson E.: SS 10.8
Johansson Synnergren M.: SS 12.10
Johnston E.: SS 2.2
Jreige M.: SS 13.1
Juel M.A.: SS 5.6
Jung H.-S.: SS 10.4

### K

Kalarakis G.: SS 13.2
Kale A.: SS 10.9
Kale S.K.: **SS 11.3**
Kalogeropoulou C.: SS 5.4
Kamal H.: SS 8.4
Kamaledeen S.: **SS 3.8**
Kanamathareddy H.: SS 3.3
Kang J.: SS 10.4
Kaposi P.: SS 1.2
Karabulut U.E.: **SS 14.2**
Karachaliou M.: SS 5.4
Karasu H.I.: SS 12.4
Karatzas A.: SS 5.4
Karim H.: SS 11.6
Kartalis N.: SS 7.1
Katz Y.: SS 1.3
Kaufmann-Bühler A.-K.: SS 12.8
Kazemier G.: SS 8.1
Kechagias A.: SS 13.2
Kechagias S.: SS 12.1
Kemmerich G.: SS 8.1
Kenou G.: SS 14.7
Kessner R.: **SS 1.3**
Khalili K.: **SS 4.8**
Khorasanizadeh F.: SS 6.4
Kim H.-D.: SS 6.1
Kim J.H.: SS 1.6
Kim J.H.: SS 3.1
Kim J.-M.: **SS 4.2**, SS 10.3, SS 10.4
Kim K.: SS 6.1
Kim S.H.: **SS 3.1**
Kim T.H.: SS 9.6, SS 9.7
Kim Y.: SS 10.4
Kirkman D.: SS 8.4
Kittinger J.: SS 7.1
Kjeldsen J.: SS 5.6
Klontzas M.: SS 13.2
Knudsen T.: SS 5.6
Koh D.-M.: SS 3.5
Kolahdoozan S.: SS 5.7
Kolrud K.: SS 8.1
Koltsakis E.: **SS 13.2**
Kop M.: SS 1.8
Kristic A.: **SS 7.1**
Kuijpers M.: SS 8.2
Kulali F.: **SS 4.9**
Kumar S.: **SS 5.1**, **SS 5.5**
Kwon R.: SS 10.4

### L

Lagerstrand K.: SS 10.6, SS 12.10
Laghi A.: SS 10.5, SS 13.8
Lahaye M.: SS 3.6, SS 11.1, SS 11.2
Lakhani A.: SS 3.3
Lambie H.: SS 13.5, SS 13.6
Lambregts D.: SS 3.6, SS 11.1, SS 11.2
Landoni L.: SS 2.7
Lange M.: SS 14.9
Lange N.F.: SS 12.2
Lazar S.: SS 1.3
Lebtahi R.: SS 4.5, SS 11.5
Leclerc P.: SS 11.5
Lee D.: SS 4.2
Lee D.H.: SS 4.2
Lee J.-H.: SS 4.2
Lee L.: SS 5.1
Lee M.: SS 11.10
Lee M.Y.: **SS 4.1**
Lee S.: SS 8.4
Lee S.: SS 4.1
Lee S.S.: SS 6.1
Lefere M.: **SS 1.1**
Lehmann K.: SS 14.9
Lequoy M.: SS 4.5
Levy P.: SS 8.9
Levy V.: SS 4.5
Levy V.: SS 13.1
Lewin M.: SS 4.5
Li L.: SS 8.7
Licata C.: SS 8.8
Lim G.-Y.: SS 10.4
Lindsay L.: SS 5.10
Linge J.: SS 12.1
Liu X.: SS 4.8
Ljuslinder I.: SS 3.2
Lohse A.-K.: SS 11.4, SS 11.6
Lovász B.: SS 1.2
Lubner M.G.: **SS 7.8**
Lubner S.J.: SS 7.8
Luciani A.: SS 4.5

### M

Maas M.: SS 3.6
Maino C.: **SS 7.3**, SS 9.1, SS 13.4
Maire F.: SS 8.9
Mak A.: SS 10.7
Maksud P.: SS 4.5
Mal H.: SS 12.9
Malalagama G.: SS 9.10
Malekzadeh S.: **SS 1.4**
Mallett S.: SS 5.1, SS 5.5
Mandorfer M.: SS 12.5
Mansournia S.: SS 11.4, SS 11.6
Mansueto G.: SS 8.10
Mantziari S.: SS 13.1
Mao L.: SS 7.8
Marchetti S.: SS 11.1, SS 11.2
Mariani I.: **SS 9.1**
Mariano Goulart D.: SS 4.5
Marquering H.: SS 1.8, SS 8.1
Martínez Rivero S.: SS 1.5
Mascarin B.: **SS 1.5**, SS 7.7, SS 11.8
Masci B.: SS 10.5
Masih D.: SS 13.3
Matteini F.: SS 6.4, **SS 10.2**, **SS 14.6**
Maurovich-Horvat P.: SS 1.2
Mazzola M.: SS 10.2
Melling P.: SS 13.5, SS 13.6
Ménez O.: **SS 8.5**
Meng X.: **SS 3.9**
Menu Y.: SS 4.5
Menys A.: SS 5.2, SS 5.3, SS 5.5, SS 5.9
Merle P.: SS 2.6
Messner A.: SS 7.1
Metens T.: SS 8.5
METRIC-EF Study Investigators: SS 5.1
Micelli A.: SS 3.4, SS 3.7
Middelburg T.: **SS 7.4**
Milazzo M.: **SS 12.9**
Moga L.: SS 14.8
Mohammadzadeh N.: SS 14.3
Mokhles S.: **SS 8.2**
Mol B.: SS 7.4
Moloney B.: SS 4.8
Montravers F.: SS 4.5
Mookhoek A.: SS 5.8
Moos S.: SS 8.1
Moran G.W.: SS 5.9
Moreno-Rojas M.J.: **SS 2.1**
Morra M.: SS 1.5
Mottet C.: SS 1.4
Mougin O.: SS 5.9

Muharam H.: SS 8.2
Mukherjee A.: SS 5.9
Mule S.: SS 4.5
Muthoo C.: **SS 9.9**, **SS 13.5**, **SS 13.6**

### N

N'Kontchou G.: SS 2.6
Nacci I.: SS 10.5
Nacher M.: SS 14.7
Nahon P.: SS 2.6
Naim I.: SS 5.2, SS 5.3, **SS 5.9**, SS 5.10
Nanda B.P.: SS 4.8
Nasr P.: SS 12.1
Nassiri-Toosi M.: SS 6.10
Nault J.-C.: SS 2.6, **SS 4.5**
Nederend J.: SS 13.7
Nederveen A.J.: SS 7.4, SS 10.7
Neefjes-Borst E.: SS 5.8
Nieuwdorp M.: SS 10.7
Nio Y.: SS 1.8
Nota I.: SS 8.1
Nowak K.: SS 4.8
Nuzzo A.: SS 14.5, SS 14.6

### O

Obmann V.C.: SS 12.2
Okten R.S.: SS 12.6
Omboni F.: SS 8.10

### P

Pageaux G.-P.: SS 4.5
Paisant A.: SS 8.3
Palic S.: SS 1.8
Palmqvist R.: SS 3.2
Paradis V.: SS 11.9
Park J.: **SS 1.6**
Park S.J.: SS 4.2, SS 10.3, **SS 10.4**
Parry T.: SS 5.1, SS 5.5
Pasquazzo F.: SS 1.7, SS 2.8
Patel J.: SS 5.1
Patel V.: SS 8.4
Payancé A.: SS 12.9
Pekindil G.: SS 10.9
Pelanis E.: SS 8.1
Pérez-Serrano C.: SS 2.1
Petit A.: SS 2.6
Petkovska I.: SS 9.6
Pickhardt P.J.: SS 7.8, SS 11.10
Pieterman K.: SS 8.1
Pijl M.: SS 13.7
Plaforet V.: **SS 14.8**
Platt J.R.: **SS 13.10**
Plumb A.: SS 5.1
Pochepnia S.: SS 7.1, SS 12.5
Poetter-Lang S.: SS 7.1, SS 12.5
Pol S.: SS 4.5
Polanski S.: SS 7.8
Polat Y.: SS 14.2
Polici M.: **SS 13.8**
Pollok R.: SS 5.1
Ponsioen C.: SS 7.4
Pop G.: SS 4.5
Poroes F.: **SS 8.6**
Porrello G.: SS 11.9
Pucciarelli F.: **SS 10.5**
Pugliese D.: SS 13.8
Punt C.: SS 8.1
Pušeljić M.: **SS 12.8**
Puyraimond-Zemmour J.: SS 2.9

### Q

Qin Y.: SS 4.7
Quaia E.: SS 3.4, SS 3.7
Quirke P.: SS 9.9

### R

Radmard A.: **SS 5.7**
Radosevic A.: SS 2.1
Rafaelsen S.: **SS 5.6**
Rahman S.: SS 5.2, SS 5.5
Raine T.: SS 5.1
Rao N.: SS 5.5
Rautou P.-E.: SS 12.9, SS 14.8
Rea B.: SS 7.2, **SS 7.5**
Rebours V.: SS 8.9, SS 11.5
Reddy H.V.: SS 13.3
Regnault H.: SS 4.5
Reiberger T.: SS 12.5
Rejler M.: SS 12.1
Ri M.: SS 13.2
Ricke J.: SS 11.4, SS 11.6
Riklund K.: SS 3.2
Rivas E.: SS 2.1
Roche G.: SS 8.4
Rode A.: SS 2.6
Rodriguez L.: SS 9.6
Roe C.: SS 13.5, SS 13.6
Roger P.: SS 12.9
Rohan T.: SS 1.9
Roman M.: SS 1.5
Ronot M.: SS 2.3, SS 2.9, SS 4.5, SS 4.7, SS 6.5, **SS 8.9**, SS 10.1, **SS 11.5**, SS 11.9, SS 12.9, SS 14.5, SS 14.6, SS 14.8
Rorsman F.: SS 10.8
Rossington H.: SS 9.9
Rothenbühler K.: **SS 14.4**
Rotzinger D.: SS 14.4
Rouvelas I.: SS 13.2
Roux J.: SS 4.5
Ruebenthaler J.: SS 11.6
Ruiz-Osuna S.: SS 2.1
Rush B.E.: SS 11.10
Ruszniewski P.: SS 11.5
Rutegård J.: SS 3.2
Rutegård M.: SS 3.2
Rutegård M.: **SS 3.2**
Ryoo B.-Y.: SS 6.1
Ryu R.R.: SS 1.6
Ryu S.: SS 10.4

### S

Safaei M.: SS 6.10
Sahin H.: **SS 12.3**
Sahin O.: SS 12.3
Sahin S.: SS 12.6
Sahin S.: SS 12.3
Salahshour F.: **SS 6.10**, **SS 14.3**
Samarasam I.: SS 13.3
Santini P.: SS 3.4, SS 3.7
Santos J.M.: SS 9.7, **SS 9.8**
Sappenfield R.: SS 7.8
Sarovic D.: SS 10.6
Sartoris R.: SS 4.7, SS 11.9, SS 12.9
Sathyanarayan L.: SS 11.3
Sayadi A.: SS 14.4
Sbeghen P.: SS 8.10
Schafer M.: SS 13.1
Schernthaner M.: SS 12.5
Schmidt Kobbe S.: SS 8.6, SS 14.4
Schmid-Tannwald C.: SS 11.4, SS 11.6
Schütz F.: SS 8.6
Scurr E.: SS 3.5
Segbers M.: SS 7.10
Seligmann J.: SS 13.10
Sempoux C.: SS 14.4
Sengel C.: SS 4.5
Seror O.: SS 2.6, SS 4.5
Sheng L.: **SS 6.5**
Shepherd T.: SS 5.2
Shi Y.: SS 4.7
Shia J.: SS 9.6. SS 9.7
Shkurti J.: **SS 13.7**
Shumbayawonda E.: SS 10.7
Shur J.: SS 2.2, **SS 3.5**
Sidali S.: SS 4.5
Siddiqui M.S.: SS 8.4
Singh A.: SS 13.3
Sivakumaran T.: SS 11.2
Sjögren D.: SS 12.1
Snaebjornsson P.: SS 11.1, SS 11.2
Somashekhar S.P.: SS 11.3

Song B.: SS 4.6, SS 4.7, SS 6.2, SS 6.5, SS 6.6, SS 6.7, SS 9.5
Soussan M.: SS 4.5
Sponza M.: SS 2.4
Spoto F.: **SS 1.7**, SS 2.8, SS 7.7, **SS 8.8**, SS 11.8
Šprláková-Puková A.: SS 1.9
Stenqvist E.: SS 13.2
Stephenson J.: SS 5.1
Stjernman H.: SS 12.1
Stocker D.: SS 14.9
Stocker S.: **SS 14.9**
Stoker J.: SS 1.8, SS 5.8, SS 7.4, SS 8.1
Stratulat I.: **SS 3.10**
Struik F.: SS 1.8
Sukhanenko M.: SS 7.5
Summers R.M.: SS 11.10
Sunnegårdh J.: SS 12.10
Sussan G.: **SS 3.4**, **SS 3.7**
Sutter O.: **SS 2.6**
Svensson P.: SS 12.10
Swijnenburg R.-J.: SS 8.1
Syversveen T.: SS 8.1
Szijártó A.: SS 1.2
Szűcs Á.: SS 1.2

### T

Taama I.: SS 8.10
Taher M.: SS 6.10
Taherzadeh M.: SS 6.10
Talakić E.: SS 12.8
Talei Franzesi C.: SS 13.4
Tanga L.: SS 14.7
Tarhan S.: SS 10.9
Tasdemir M.N.: SS 12.4
Taylor S.A.: SS 5.1, SS 5.5
Tekdemir H.: SS 12.6
Terkivatan T.: SS 8.2
Thomeer M.: SS 7.10, SS 8.2
Tielbeek J.: SS 5.8
Tirkes T.: **SS 8.7**
Todesco M.: SS 1.7, SS 2.7, **SS 2.8**, SS 7.7, SS 11.8
Tognella A.: SS 14.9
Tolan D.: SS 5.1, SS 9.9, SS 13.5, SS 13.6, SS 13.10
Toprak H.: SS 14.2
Travis S.: SS 5.1
Triantos C.: SS 5.4
Trillaud H.: SS 2.6
Troelstra M.A.: SS 10.7
Troxler R.: SS 14.4
Tse M.: SS 13.5, SS 13.6
Tual A.: SS 14.5
Tzadok R.: SS 1.3
Tzortzakakis A.: SS 13.2

### U

Uldry E.: SS 8.6

### V

Valanzuolo D.: SS 13.8
Valdman A.: SS 3.10
Valla D.: SS 10.1
van den Bergh J.: SS 8.1
van der Meer A.: SS 7.4
van der Meulen D.: SS 8.1
van Golen L.: SS 11.1, SS 11.2
Van Os Z.: SS 8.2
van Rijn K.: SS 5.8
van Son K.C.: SS 10.7
van Waesberghe J.-H.: SS 8.1
Vassallo J.: **SS 6.9**
Veenstra M.M.: **SS 7.10**, SS 8.2
Vega R.: SS 5.1
Vegt E.: SS 7.10
Venkatesh S.: SS 7.1
Verburg F.: SS 7.10
Vergeau A.-P.: SS 14.6
Verheij J.: SS 10.7
Vernuccio F.: SS 3.4, SS 3.7
Verpalen I.: **SS 8.1**
Vessby J.: SS 10.8
Vietti Violi N.: SS 8.6, SS 13.1
Vilgrain V.: SS 2.3, SS 2.9, **SS 8.3**, SS 10.1, SS 11.9, SS 14.5, SS 14.6
Vit A.: SS 2.4
Vogel W.: SS 11.1
Vollebergh M.: SS 11.1, SS 11.2

### W

Wagenaar L.: SS 8.1
Wagner A.: SS 13.1
Wagner M.: SS 4.5
Wåhlander H.: SS 12.10
Wang Y.: SS 4.7, SS 6.5, SS 6.6
Wartski M.: SS 4.5
Wassenaar N.P.: **SS 10.7**
Watzinger N.: SS 12.8
Weiland E.: SS 8.5
Weng J.: SS 5.1
Wesdorp N.: SS 8.1
West N.: SS 13.10
Weston W.: **SS 2.2**
Whitcher B.: SS 3.5
White C.: SS 9.6
White O.: SS 2.2, SS 3.5
Widmer L.: SS 1.4
Wild S.: SS 10.4
Willemse J.: **SS 11.1**, SS 11.2
Willemssen F.: **SS 7.6**
Williams K.: SS 5.10
Winfield J.: SS 3.5
Winkelmann M.: SS 11.4, SS 11.6
Worsley C.: SS 6.9
Wright C.: SS 7.5
Wu D.: SS 6.9
Wu W.: SS 3.2
Wu Y.: **SS 4.6**
Wu Z.: SS 4.7

### X

Xie P.: SS 3.9

### Y

Yadav D.: SS 8.7
Yang C.: **SS 6.2**
Yang H.K.: **SS 4.1**
Yashar H.: SS 1.3
Ye Z.: SS 4.6
Yılmaz T.: SS 14.2
Yoo C.: SS 6.1
Yoo J.: SS 3.1
Yoon S.: SS 10.4
Young L.: SS 7.4
Yüksek H.H.: SS 10.10

### Z

Zamani S.: SS 5.7
Zambon Bertoja J.: SS 3.4, SS 3.7
Zamboni G.A.: SS 8.10
Zappa M.: SS 14.1, SS 14.7
Zealley I.: SS 5.1
Zebardast J.: SS 14.3
Zeeuw M.: SS 8.1
Zerunian M.: SS 10.5, SS 13.8
Zhang X.: SS 9.5
Zheng L.: SS 3.9
Zheng T.: SS 6.5
Zhou X.: SS 7.9
Ziol M.: SS 2.6
